# Cultivar- and phase-specific effects of light on kernel number and compensation via kernel weight in wheat

**DOI:** 10.1093/plphys/kiag277

**Published:** 2026-07-23

**Authors:** Khadija Sabir, Hartmut Stützel, Tsu-Wei Chen

**Affiliations:** Institute of Horticultural Production Systems, Leibniz University Hannover, Hannover, Germany; Institute of Horticultural Production Systems, Leibniz University Hannover, Hannover, Germany; Group of Intensive Plant Food Systems, Albrecht Daniel Thaer Institut of Agricultural and Horticultural Science, Humboldt Universität zu Berlin, Berlin, Germany

## Abstract

Kernel number per spike (KpS) and floret fertility are critical traits for enhancing wheat (*Triticum aestivum*) grain yield under climate variability. The effects of light on KpS and other yield-related traits depend on intensity, duration, developmental phase, and genotype characteristics. Here, we investigated the effects of low light during a pre-anthesis period around 100 °Cd before heading and a post-anthesis period around 240 °Cd after heading. Five light regimes were applied in split-plot experiments under controlled conditions, and 16 genotypes were tested to demonstrate the genetic diversity in response to phase-specific light regimes. The pre-anthesis subphase between yellow anther and tipping (YA-TP) was most crucial for floret fertility and kernel number. Also, the ability to compensate the loss in grain number through increased thousand kernel weight was genotype specific, especially between YA-TP. No effects correlated with the length of any phenological subphase, indicating that the differences in sensitivity are not simply a consequence of genotypic variations in phenological duration. This allowed us to identify insensitive and sensitive genotypes to low light. Furthermore, insensitive genotypes showed increases in kernel number at top spikelet positions in comparison to the sensitive genotypes. Also, both modern and older genotypes included sensitive as well as insensitive genotypes. Our results suggest genotypic differences in environmental sensitivity during the YA-TP subphase that could be further targeted for enhancing yield stability.

## Teaser

Light levels before flowering strongly shape wheat grain number and weight, with some varieties showing resilience, offering targets to breed crops with more stable yields under changing climates.

## Introduction

Enhancing the yield potential and stability of wheat (*Triticum aestivum*) is a priority to ensure global food security (Reynolds et al. 2012). Wheat yield is an extremely complex trait and is determined by its components such as spike number per unit ground area, kernel number per spike (KpS), and thousand kernel weight (TKW) (Fischer 2011). Although breeding efforts have enhanced yields in winter wheat predominantly through an increase in KpS rather than kernel weight (Würschum et al. 2018; Voss-Fels et al. 2019b; Lichthardt et al. 2020; Sakuma and Schnurbusch 2020), KpS remains a key factor limiting wheat yield potential, as over half of the potential kernels may be lost during the pre-anthesis phase owing to floret abortion (Guo et al. 2015, 2017; Sakuma and Schnurbusch 2020). KpS is determined by the number of spikelets per spike and the number of fertile florets produced per spikelet (Langer and Hanif 1973; Kirby 1988; Miralles et al. 1998). During spike development, a finite number of spikelets, typically 15 to 30 in the modern cultivars (Voss-Fels et al. 2019a), are initiated until terminal spikelet formation (Kirby and Appleyard 1981). Each spikelet initiates a variable number of floret primordia, ranging from 8 to 12. However, floret abortion accounts for ∼70% of potential kernel loss in every spikelet development (Guo and Schnurbusch 2015) and typically, only 3 to 5 florets survive and develop fertile embryos, and the rest are aborted (Sadras and Slafer 2012).

Floret fertility is the consequence of physiological progresses related to floret development and abortion (Guo et al. 2018b). The developmental progress of the spike has been divided into 8 pre-anthesis subphases: double ridge (DR), terminal spikelet (TS), white anther (WA), green anther (GA), yellow anther (YA), tipping (TP), heading (HD), and anthesis (AN) (Guo and Schnurbusch 2015). Numerous studies have explored the interactive effects of phenology and environmental conditions on kernel number (for a review, see Fischer et al. 2024). A close association between KpS and subphase durations (eg, YA and TP [YA-TP] and TP and AN [TP-AN]) has been identified by shared quantitative trait loci (QTLs), suggesting their significance and relevance to improve floret fertility and KpS (Guo et al. 2015, 2018a). KpS is influenced by the accumulation and availability of photoassimilates for stem and spike growth during the period between terminal spikelet and anthesis (Guo et al. 2018a; Lichthardt et al. 2020). Therefore, low light from beginning of stem elongation till HD reduced yield and spike dry weight, indicating the importance to maintain source strength during spike development (Fischer 1985; Savin and Slafer 1991; Slafer et al. 1994; Fischer et al. 2024). Reduced leaf photosynthetic rate and damaged leaf photosynthetic apparatus caused by low light during the young microspore stage (10 to 12 days before HD) result in the reduction of KpS (Dong et al. 2019; Yang et al. 2020). Moreover, low light during pre-anthesis also interferes with the development of pollen and ovary, leading to reduced florets fertility and KpS (Yang et al. 2022). However, studies investigating these phenomena frequently employ prolonged shading treatments, such as 28 days at 55% light reduction (Fischer et al. 2024), which do not realistically mimic natural light fluctuations. Therefore, the impact of short-term light variability is not confined to distinct developmental stages. Given the facts that (i) experimental studies investigating the effects of varied light intensity on floret and kernel development are very labor intensive and (ii) there are significant interactions between genotype, environment, and phenology, much of the literature studying light effects are not directly comparable and the conclusion could be genotype specific and less generalizable. To overcome this limitation, a statistical approach was proposed recently showing the stage- and genotype-specific sensitivity of KpS to global radiation between YA and TP (Sabir et al. 2023a). This approach effectively estimated the sensitivities of 220 genotypes to short-term environmental fluctuations across 81 developmental stages and identified genotype-specific sensitivity of KpS to light fluctuations in a pre-anthesis period around 100 °Cd before HD and a post-anthesis period around 240 °Cd after HD (Sabir et al. 2023a). However, experimental validation of this statistical approach is still required. Thus, it can be hypothesized that the effects of light on KpS depend on intensity, duration, growth subphase, and genotype characteristics.

Yield potential is determined during pre-anthesis but finalized during the post-anthesis period, where potential kernels can be further lost due to kernel abortion (Patrick and Colyvas 2014; Reynolds et al. 2022). Thus, a higher number of spikelets per spike and florets per spikelet do not guarantee more KpS as it can be annulled by increased kernel abortion. Also, TKW is realized at post-anthesis phases (Reynolds et al. 2022; Slafer et al. 2023). Light intensity during the post-anthesis period significantly impacts KpS and TKW, with effects depending on the extent of light reduction. For example, severe light reduction (35% of full sunlight from anthesis to maturity) negatively affected TKW and KpS, but slight light reduction (88% of sunlight) enhanced TKW, KpS, and overall grain yield by delaying leaf senescence and increased dry matter accumulation during grain filling (Xu et al. 2016). In the context of source–sink balance, both potential KpS and TKW are sink components after anthesis that are affected by light-derived carbon availability. As a result, a trade-off between TKW and KpS is commonly observed, with individual kernel weight often negatively correlating with KpS (Sakuma and Schnurbusch 2020). This trade-off can also arise from the “compensatory effect,” where a reduction in KpS before or after anthesis is offset by an increase in TKW during grain filling. The extent of this trade-off varies with environmental conditions and differs among genotypes (Quintero et al. 2018). This indicates that the source–sink relationship is modulated by genotype-specific trade-offs or compensatory mechanisms among yield components (Reynolds et al. 2012), which can be influenced by fluctuating environmental conditions. Given the increasing weather variability during summer across Europe (Mäkinen et al. 2018; Bönecke et al. 2020), understanding how these environmental fluctuations affect this balance is crucial.

By comparing kernels between different spikelets and within a spikelet, they are heterogeneous in terms of developmental stage, weight, and cell number (Li et al. 2016). The optimal environmental conditions increase ovary size, especially for ovaries at distal positions of a spikelet, leading to an increased sink strength at distal positions (Guo et al. 2018b). However, the positional effects (spikelet and floret position) that regulate the floret survival or abortion are less studied. Additionally, how these positional effects interact with short-term differences in light regimes during the key subphases of floret fertility is not well understood. It is also unclear whether this interaction is genotype specific.

As KpS and spike fertility are crucial traits for further increasing wheat yield (Pedro et al. 2012; Reynolds et al. 2012; Elía et al. 2016; Mirabella et al. 2016), it is essential to investigate the genotype-specific sensitivity of floret and kernel formation and their interactions with phase-specific, short-term environmental fluctuations during pre- and post-anthesis in a subphase-specific manner. However, experimentally screening a large number of genotypes under subphase-specific environmental manipulations to identify genetic resources less sensitive to these effects on KpS is impractical. The abovementioned statistical approach to identify the impact of short-term environmental fluctuations on kernels per spike (Sabir et al. 2023a) suggested high sensitivity between the YA and TP stages. However, this has not been proven experimentally. In the current study, we aim to: (i) provide experimental evidence that short-term light fluctuations affect KpS in a genotype and subphase specific manner, using 16 genotypes (Table 1); (ii) investigate the effects of these fluctuations on KpS at the spikelet level; (iii) assess the extent to which kernel loss due to light fluctuations can be compensated by increased TKW (compensatory effect); and (iv) demonstrate the genotype and subphase specific nature of this compensatory effect.

**Table 1:** Genotypes studied and their sensitivity to light intensity at 2 identified phenological periods (groups) on the basis of the results of statistical approach of Sabir et al. (2023a).

Genotype	Release year	Country of registration	Maturity	Groups
Intro	2011	Germany, France	Late	LS
Brilliant	2005	Germany	Late	LS
Ibis	1991	Germany	Late	LS
Kobold	1978	Germany	Late	LS
Avalon	1980	Great Britain	Early	S
Nimbus	1975	Germany	Early	S
Vel	—	United States of America	Early	S
Sponsor	1994	France, Ireland	Late	S
Kronjuwel	1980	Germany	Late	S1
Kurt	2013	Germany	Late	S1
Orestis	1988	Germany	Late	S1
Soissons	1987	Belgium, Spain, France, Ireland, Italy, Slovenia	Early	S1
SW Topper	2002	Germany	Late	S2
Kontrast	1990	Germany	Early	S2
Edward	2013	Germany	Late	S2
Alidos	1987	Germany	Late	S2

LS, less sensitive at both pre- and post-anthesis periods; S, sensitive at both pre- and post-anthesis periods; S1, sensitive at only pre-anthesis period; S2, sensitive at only post-anthesis period.

## Results

### Thousand kernel weight, floret number, and kernel number showed subphase-specific response to short-term light reduction

**Figure 1:**
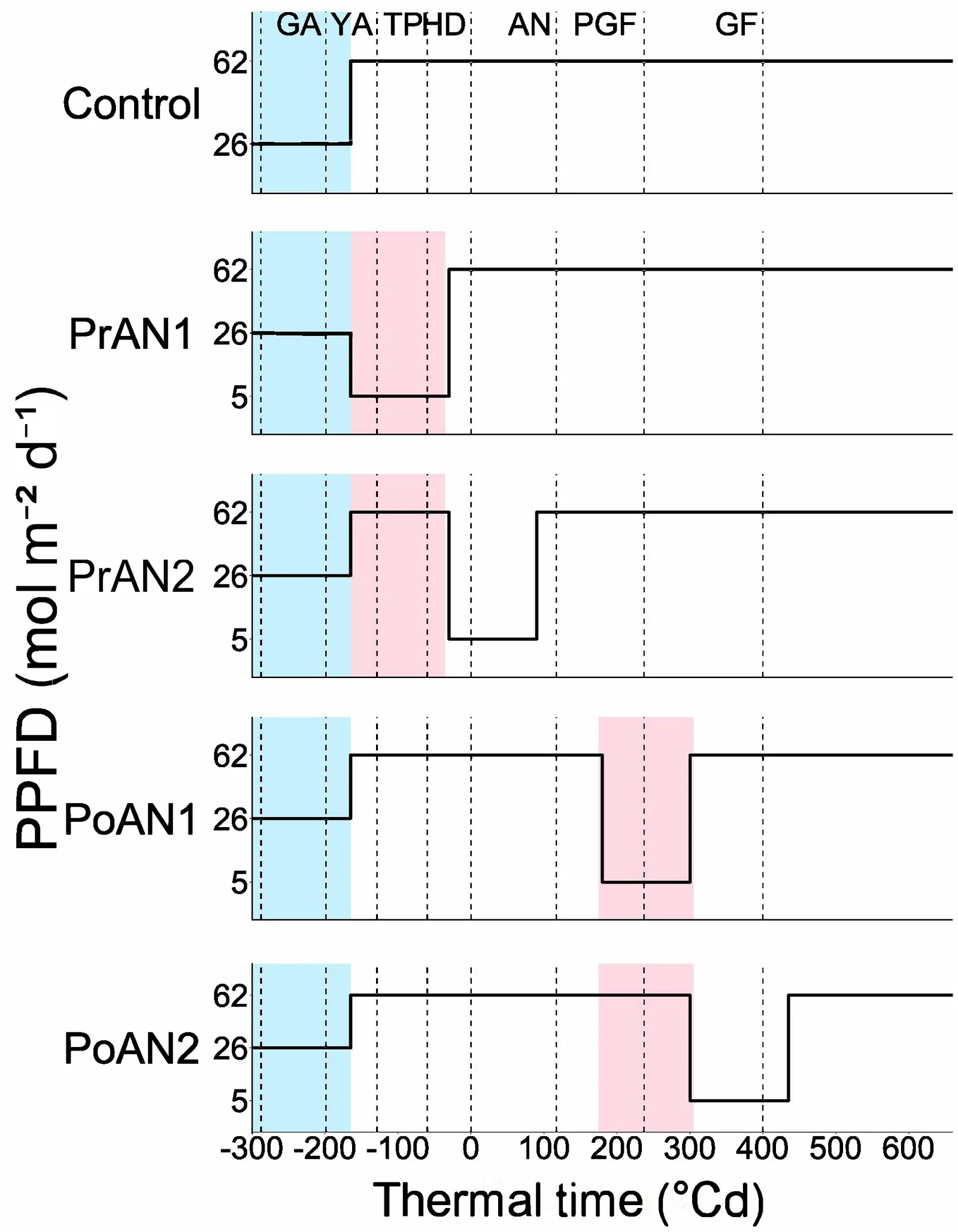
Visualization of light regimes, corresponding developmental subphases, and thermal time (*x*-axis, °Cd) from heading (°Cd = 0). The *y*-axis represents different levels of photosynthetic photon flux density (5, 26, and 62 mol m^−2^ d^−1^) applied during specific developmental subphases across 5 treatments. PrAN1: low light during sensitive pre-anthesis phase 1; PrAN2: low light during insensitive pre-anthesis phase 2; PoAN1: low light during sensitive post-anthesis phase 1; PoAN2: low light during insensitive post-anthesis phase 2. In all treatments, plants were acclimated in a growth chamber under moderate light intensity (26 mol m^−2^ d^−1^) between the green anther and yellow anther stages (−300 to −165 °Cd, blue shaded area) prior to initiating the specific light treatments. In each treatment, low light (5 mol m^−2^ d^−1^) was used in subphases indicated at the top. The identified sensitive periods are highlighted by pink shaded areas. GA, green anther, YA, yellow anther; TP, tipping; HD, heading; AN, anthesis; PGF, pre-grain filling; and GF, grain filling. The thermal time at which the treatment started was cultivar specific (Figure S2) and depended on phenology.

**Figure 2:**
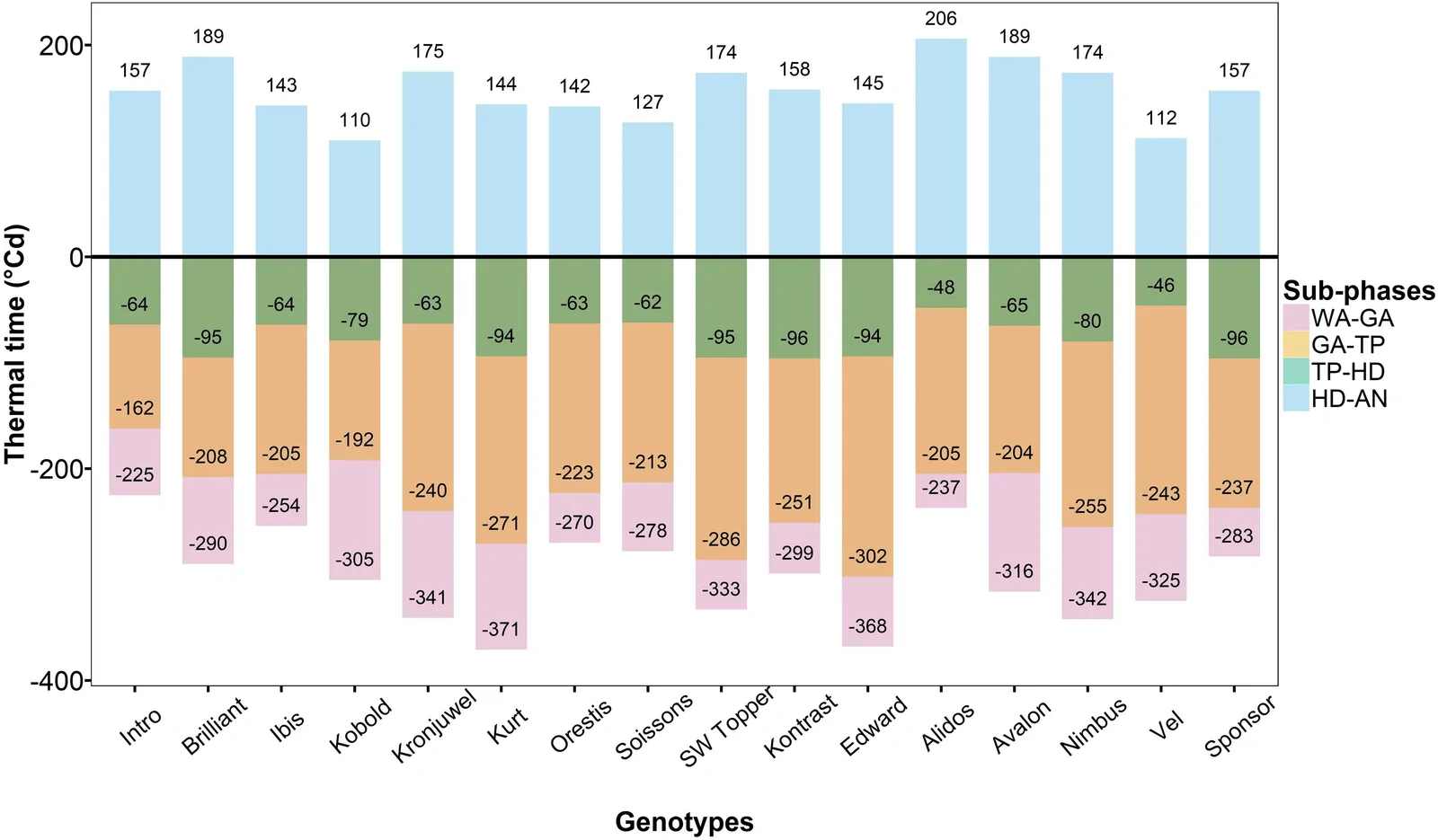
Measured subphase duration of the studied genotypes before and after heading (HD; thermal time = 0 °Cd, solid black line). WA-GA, from white anther to green anther subphase; GA-TP, from green anther to tipping subphase; TP-HD, from tipping to heading subphase; HD-AN, from heading to anthesis subphase. The thermal time at which the treatment started was cultivar specific (Figure S2) and depended on phenology.

To test the subphase and genotype-specific effects of light intensity on the formation of KpS, low light was applied at 4 periods (Fig. 1). Two periods belong to pre-anthesis (PrAN1 and PrAN2) and 2 from post-anthesis (PoAN1 and PoAN2) developmental subphases. Since the lengths of phenological subphases differed between genotypes (Fig. 2), the timing of treatment initiation was genotype specific (Figure S1, Figure S2), while treatment duration remained constant across all genotypes to ensure comparability. Consequently, the proportion of the treatment within each phase varied by genotype, potentially creating an artifact where longer subphases reduced the treatment's relative impact. However, we carefully evaluated this unavoidable experimental dilemma and found no link between the impact of low light and the length of phenological subphases (see later section).

**Figure 3:**
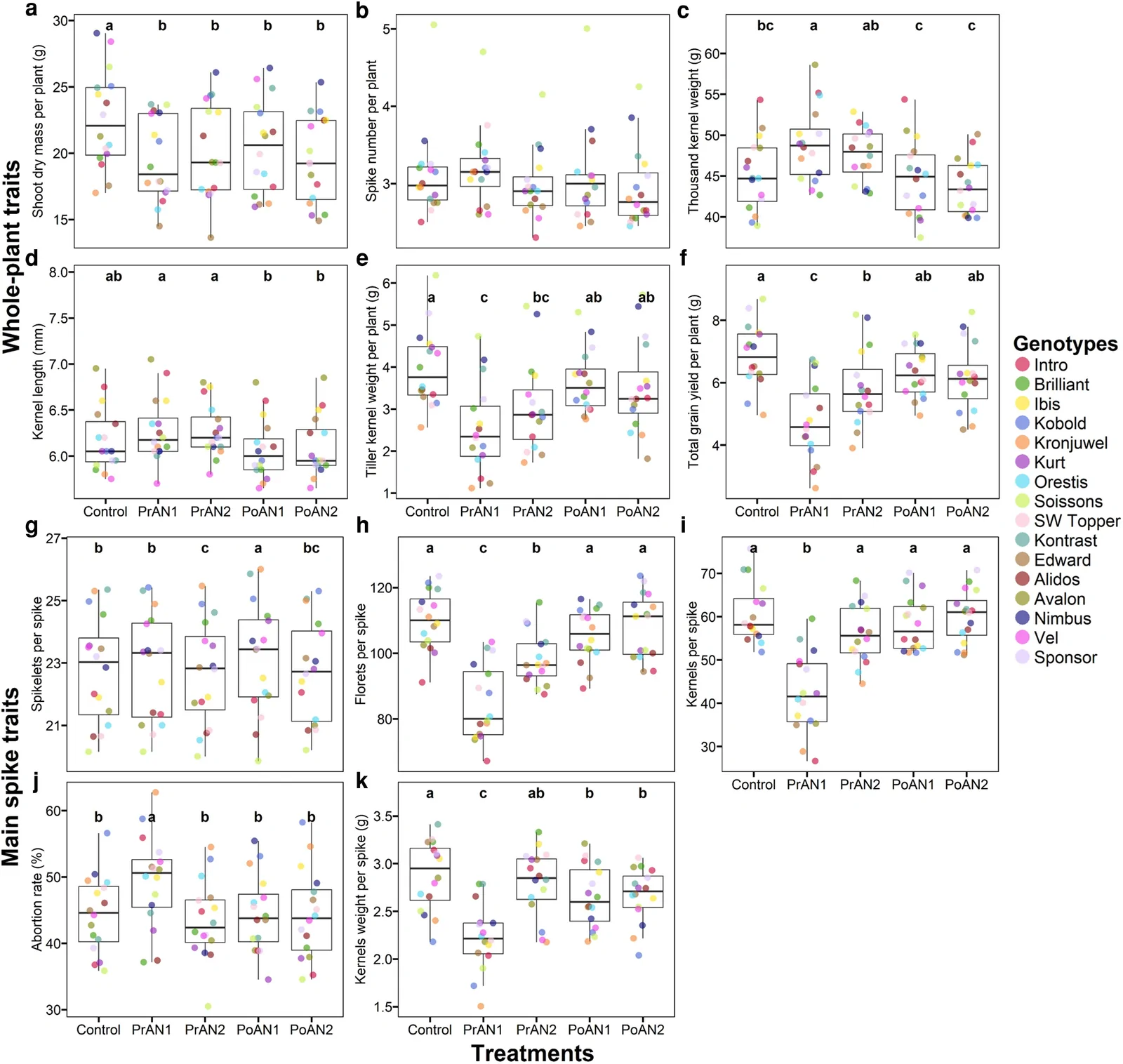
Effects of light treatments on whole-plant and main spike traits. (a) Total shoot dry weight per plant (g); (b) number of spikes per plant, (c) thousand kernel weight (g); (d) kernel length (mm); (e) total kernel weight of all tillers per plant (g); and (f) total grain yield per plant (g); (g) number of spikelets per main spike; (h) number of florets per main spike; (i) number of kernels per main spike; (j) abortion rate per main spike (%); and (k) total kernel weight of main spike (g). Each point represents the mean value for a genotype (colored points), averaged over 2 replications (10 plants per replication). Different letters indicate significant differences between treatments according to the least significant difference (LSD) test at *P* ≤ 0.05. The treatments include: Control, with high light throughout the experimental period; PrAN1, where low light was applied during the sensitive YA-TP period followed by high light during the HD-AN subphase, and vice versa in PrAN2; PoAN1, where low light was applied during the sensitive PGF to early GF subphase followed by high light during GF, and vice versa in PoAN2 (see Fig. 1 for details). Box plot shows median as the center line, with the box spanning from the first quartile (Q1, 25th percentile) to the third quartile (Q3, 75th percentile), representing the middle 50% of the data. The interquartile range (IQR) is the difference between Q3 and Q1.

Compared to control, all light treatments reduced total shoot dry mass (g) by 9.25% to 13.57% (Fig. 3a) and there were subtle differences in number of spikelets on the main spike (Fig. 3g). It is important to emphasize that none of the treatments affected the spike number per plant (Fig. 3b). Therefore, the treatment effects on total grain yield per plant (Fig. 3f) primarily resulted from their impact on KpS and TKW (Fig. 3c, e). In contrast, total floret per spike was only sensitive to pre-anthesis light treatment, resulting in a reduction of 23.01% and 10.78% in floret number under PrAN1 and PrAN2, respectively (Fig. 3h). Interestingly, the reduction of floret number did not affect kernel number on the main spike in PrAN2, but in PrAN1, KpS was reduced by 31.43% (Fig. 3i), owing to the increased abortion rate by 11.42% (Fig. 3j). This indicates phase-specific effects of light on KpS. Different from our expectation, post-anthesis treatments exhibited in general no negative effects on the traits of main spike. Although KpS at the whole-plant or canopy level was not measured due to the time-intensive counting process, the results appear scalable to the whole-plant level. This is supported by the similar pattern of light fluctuation effects on kernel weight at both the whole-plant (Fig. 3e, f) and main spike levels (Fig. 3i, k), as well as the unchanged spike number per plant (Fig. 3b).

Compared to the control, TKW increased by 8.32% in PrAN1 and 5.37% in PrAN2, suggesting a compensatory relationship between TKW and KpS (Fig. 3c). In contrast, TKW was not significantly affected by post-anthesis treatments, showing a slight tendency to decrease (up to 3.31%; Fig. 3c) likely owing to the treatment's impact on kernel length (Fig. 3d). Since the potential of TKW is partly determined by cell division in the endosperm before anthesis and the realization of TKW occurs after anthesis during grain filling (Calderini et al. 2001; Reynolds et al. 2022), our data indicate that the potential of kernel weight was, in general, affected by low light during the YA-TP subphase. Also interesting is that PrAN1 reduced the main spike kernel weight and tiller kernel weight by 22.66% and 36.38% compared to the control, respectively (Fig. 3k, e), showing the phase-specific and combined effects of low light on floret survival (pre-anthesis), kernel survival (post-anthesis), and grain filling at main spike and tillers final kernel weight.

### Genotype-specific sensitivity of kernel number per spike and kernel weight to low light

**Figure 4:**
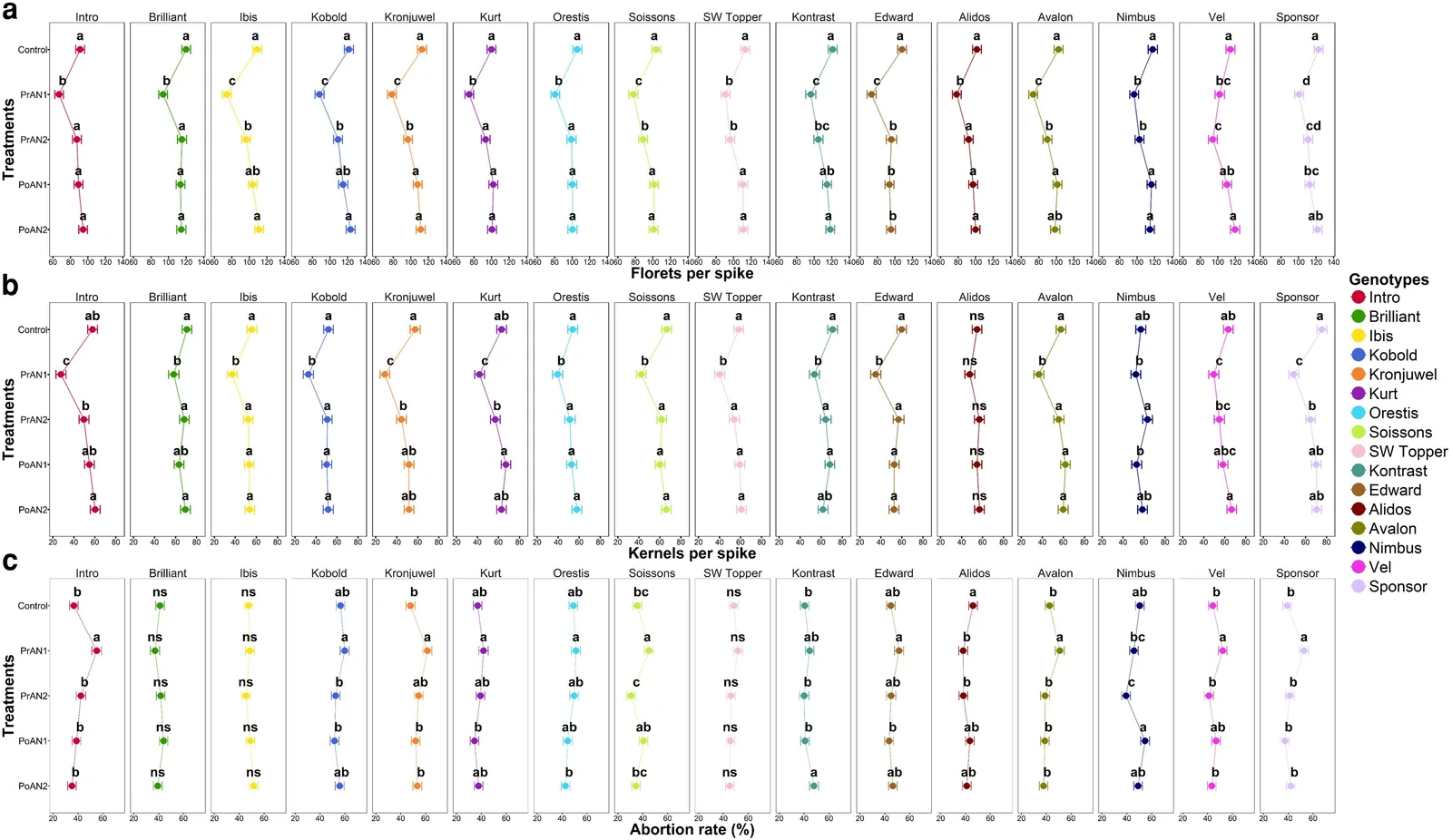
The interaction effects of genotype and light treatments. Effects of treatment on (a) florets per spike, (b) kernels per spike, and (c) abortion rate per spike (%). Each point represents the estimated marginal mean derived from the linear mixed-effects model, with 20 individual plants nested within 2 replicates/chambers. Error bars indicate 95% confidence intervals. Different letters denote statistically significant differences among treatments according to Sidak-adjusted pairwise comparisons (*P* ≤ 0.05). Control, with high light throughout the experimental period; PrAN1, where low light was applied during the sensitive YA-TP period followed by high light during the HD-AN subphase, and vice versa in PrAN2; PoAN1, where low light was applied during the sensitive PGF to early GF subphase followed by high light during GF, and vice versa in PoAN2 (see Fig. 1 for details).

To explore the genotype-specific responses, interactions between genotype and treatment were tested. Among the 11 traits analyzed (Fig. 3), significant interactions were found in florets per spike, KpS, abortion rate (Fig. 4), and main spike kernel weight (Figure S3). All genotypes showed significant decrease of 10.7% to 32.2% in florets per spike under PrAN1, and 10 genotypes exhibited reduction of 9.8% to 17.5% under PrAN2 (Fig. 4a). For KpS, 14 genotypes exhibited a significant decrease of 17.8% to 52.1% under PrAN1, while only 2 genotypes showed a decrease of 14.4% to 22.7% under PrAN2 (Fig. 4b). We emphasize that the effects of PrAN1 on KpS (Fig. 3i, Fig. 4b) were not correlated with the length of any phenological subphases (Fig. 2). Thus, no experimental artifact was observed where longer subphases diminished the treatment's relative impact.

In contrast to pre-anthesis treatments, no significant differences in KpS were observed under post-anthesis treatments PoAN1 and PoAN2 compared to the control across all genotypes (Fig. 4b). Interestingly, 2 genotypes, Alidos and Nimbus, demonstrated insensitivity, showing no significant change in KpS from control under any of the treatments (Fig. 4b). Abortion rate increased by 18.1% to 50.0% under PrAN1 in 6 genotypes (Intro, Kronjuwel, Soissons, Avalon, Vel, and Sponsor; Fig. 4c), but there was no significant increase in abortion rate under PrAN2, PoAN1, and PoAN2 in most genotypes compared to the control (Fig. 4c, with some exceptions, eg Kontrast under PoAN2). Notably, 2 insensitive genotypes (Alidos and Nimbus) showed a decrease in abortion rate by 17.2% and 9.26% under PrAN1, and by 16.9% and 22.1% under PrAN2, respectively (Fig. 4c). Furthermore, reduced main spike kernel weight was observed in 6 genotypes (Intro, Kronjuwel, Soissons, Avalon, Vel, and Sponsor) under PrAN1 (Figure S3), similar to the KpS reduction mentioned above. This shows that increased kernel abortion in PrAN1 in abovementioned 6 genotypes leads to reduced KpS and spike weight as well. However, genotypes with reduced KpS (Intro, Kronjuwel, Vel, and Sponsor) under PrAN2 as compared to control showed compensation. This compensation is manifested as a reduced abortion rate and an increase in spike weight, similar to the control (Fig. 4c and Figure S3). As the treatment-and-genotype-interaction effect on the number of florets was statistically significant, the reduction in KpS in abovementioned genotypes was consistent with a lower number of initiated florets rather than increased floret or kernel abortion.

**Figure 5:**
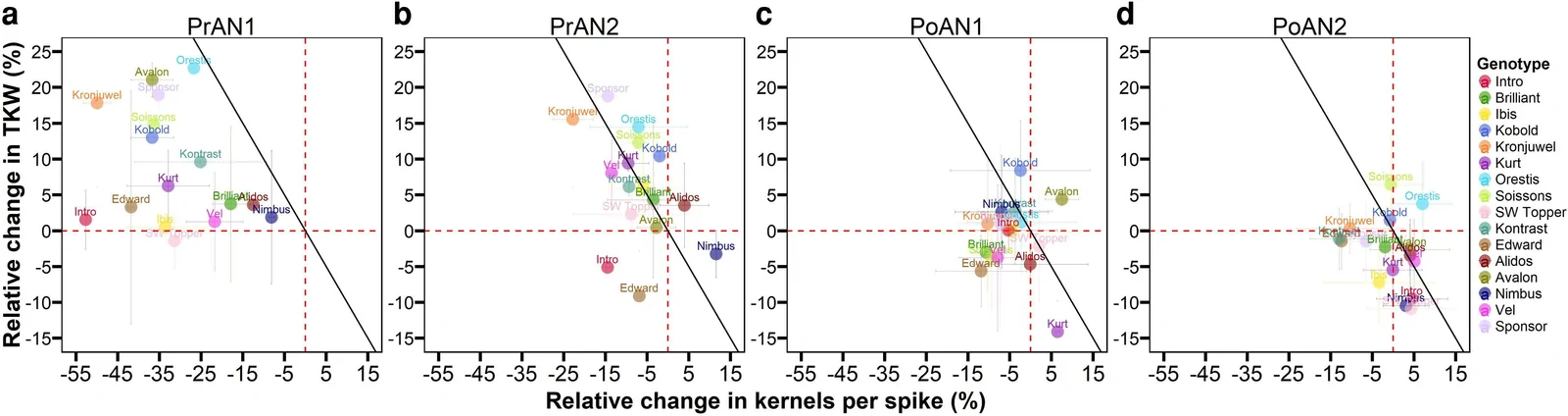
Response of thousand kernel weight per plant (relative change to control, %, *y*-axis) to change in kernels per main spike (relative change to control, %, *x*-axis). Response under pre-anthesis (a) PrAN1 and (b) PrAN2 and post-anthesis (c) PoAN1 and (d) PoAN2 light treatments. Genotypic responses under each treatment are represented by colored points in each sub-plot. A solid black line (slope = −1) indicates 100% compensation between thousand kernel weight per plant and number of kernels per main spike. Each point represents the mean value for a genotype, showing with SDs over 2 replications (10 plants per replication). PrAN1, where low light was applied during the sensitive YA-TP period followed by high light during the HD-AN subphase, and vice versa in PrAN2; PoAN1, where low light was applied during the sensitive PGF to early GF subphase followed by high light during GF, and vice versa in PoAN2 (see Fig. 1 for details).

At main spike level, increase in TKW (Fig. 3c) under PrAN1 was observed, which can be interpreted as a passive consequence of greater carbon availability per kernel. Interestingly, the relative changes in TKW and KpS had no correlation under PrAN1 (*R*^2^ = 0.06, *P* = 0.36; Fig. 5a) and the degree of increased TKW was generally lower than the loss in KpS under PrAN1. Except for Orestis, genotypes could not fully offset the reduction in kernel number with an increase in kernel weight (Fig. 5a). Under PrAN2, in contrast, most genotypes were able to compensate the KpS loss by the proportional increase in TKW (Fig. 5b). The relative changes of TKW under PoAN1 and PoAN2 were less pronounced than that under PrAN1 and PrAN2 but more negative effects on TKW were observed (Fig. 5c, d), probably owing to the treatment-induced reduction in pre-anthesis carbohydrate reserves and later the reduced source availability for grain filling.

**Figure 6:**
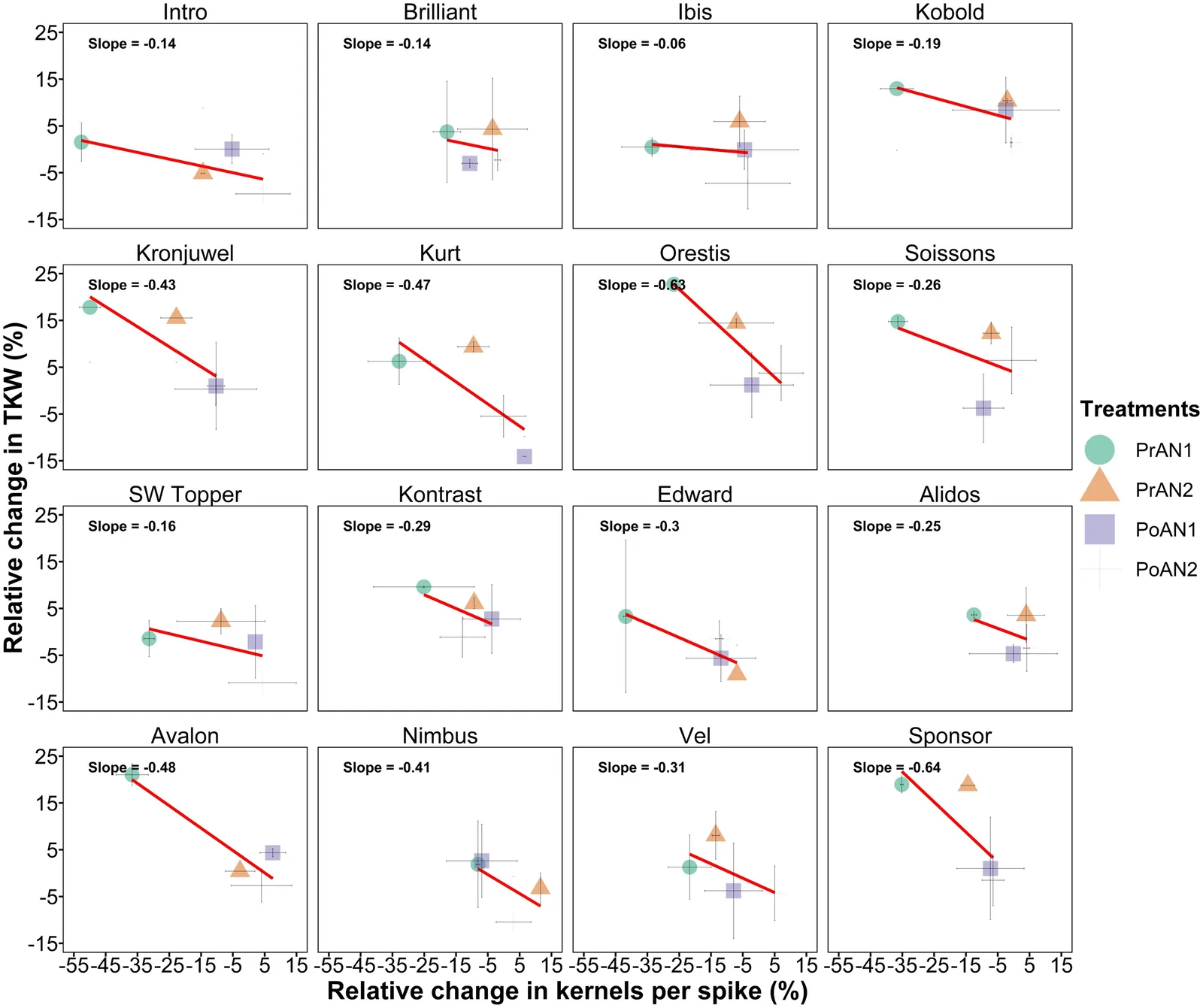
Relationship between kernels per main spike (relative change to control, %, *x*-axis) and thousand kernel weight per plant (relative change to control, %, *y*-axis) within a genotype (16 genotypes in total) to determine the compensatory effects. The colored points represent 4 treatments. Each point represents the mean value for a genotype, showing with SDs over 2 replications (10 plants per replication).

To further characterize the genotype-specific compensability, the relationship between TKW and KpS per genotype was visualized (Fig. 6). Sensitive genotypes (≥35% reduction in KpS) were Intro, Ibis, Kronjuwel, Soissons, Edward, Avalon, and Sponsor; and insensitive genotypes (≤20% reduction in KpS) were Brilliant, Alidos, and Nimbus. The sensitive genotypes were further categorized as compensable (≥15% compensatory increase in TKW, ie Kronjuwel, Soissons, Avalon, Sponsor, genotypes with stiffer negative slope) or not compensable (≤15% increase in TKW, ie Intro, Ibis, Edward). Moreover, the sensitive genotypes (Soissons, Kronjuwel, and Sponsor) showed similar increases in TKW under PrAN2 as under PrAN1 despite varied reduction in KpS under these treatments (Fig. 6).

### Genotype- and phase-specific sensitivity to light can be explained by the floret development and abortion at the spikelet level

**Figure 7:**
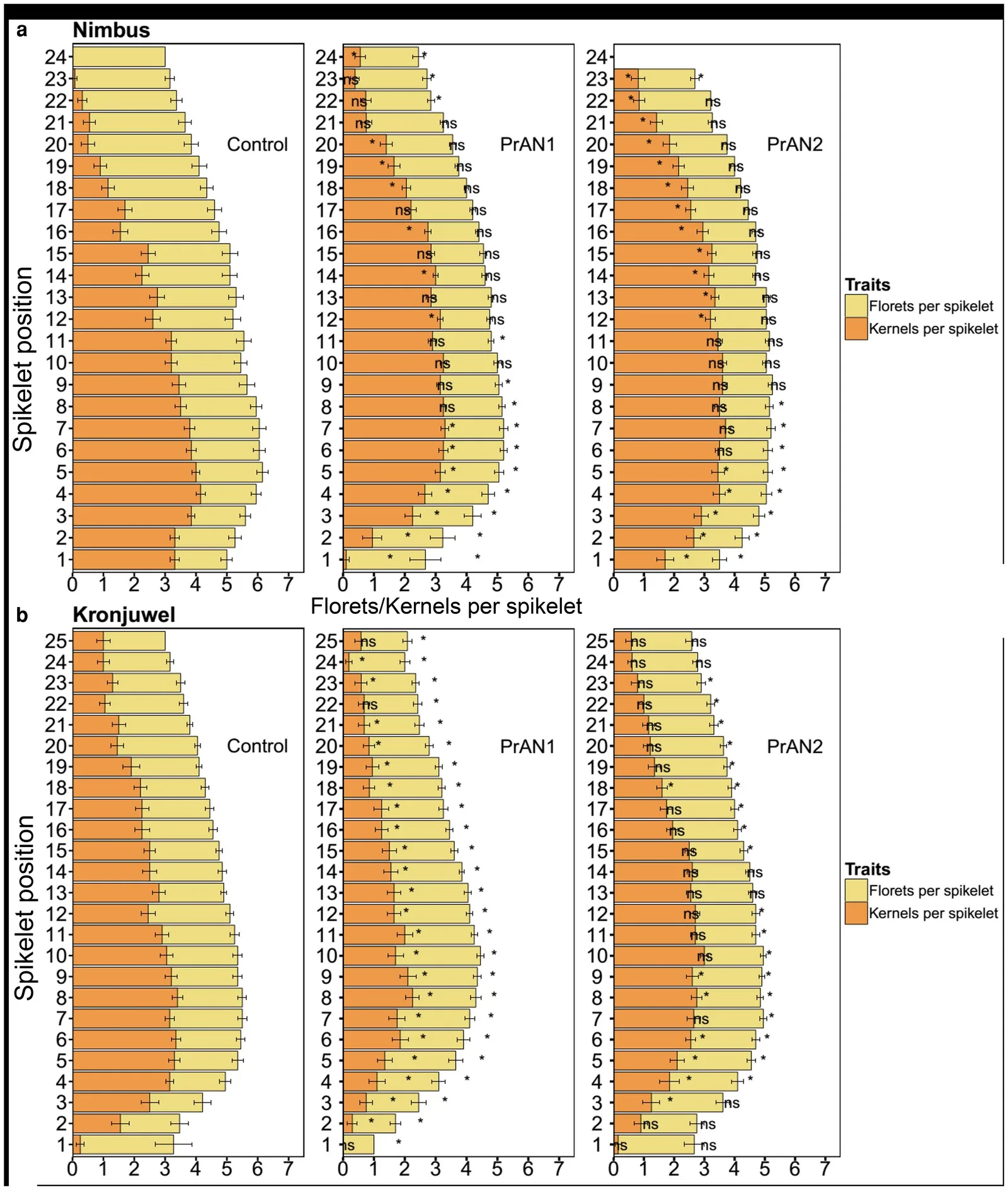
Mapping of total florets and kernels number (*x*-axis) at each spikelet position (*y*-axis) of the main spike. Data of (a) a less sensitive genotype, Nimbus, and (b) a sensitive genotype, Kronjuwel, are shown. For clarity, only data from 3 treatments’ control (left panel), PrAN1, and PrAN2 are shown. Spikelet 1 is the basal position on the spike and spikelet; 24 and 25 are terminal spikelets of Nimbus and Kronjuwel, respectively (*y*-axis). The *x*-axis indicates the total number of florets (yellow bars) and kernels (orange bars) per spikelet. Error bars indicate the standard error of the mean (n = 20). At each spikelet position, asterisks represent significant differences in means between control and PrAN1 or PrAN2 (*P* < 0.05 using *t*-test). Comparison of control with pre-anthesis (PrAN1 and PrAN2) and post-anthesis light treatments (PoAN1, PoAN2) can be found in Figures S4 and S5.

To further understand the interactions between genotypes and phase-specific effect of light reduction, data were analyzed for each spikelet across the main spike. Using 2 genotypes, Nimbus (insensitive) and Kronjuwel, as examples, a comparison of control and pre-anthesis light treatments revealed distinct responses in total florets and total kernels per spikelet across various spikelet positions (Fig. 7). In Nimbus, florets per spikelet showed similar pattern under both pre-anthesis treatments at most of the spikelet positions, with only few spikelets mainly at basal positions showing reduced florets per spikelet compared to control (Fig. 7a). Interestingly, an increase in number of kernels at the top spikelet positions was observed under both treatments (PrAN1 and PrAN2, Fig. 7a). A similar pattern of increased kernel numbers at the top spikelet positions was observed in the other two insensitive genotypes, Alidos and Brilliant (Figure S4). Under both pre-anthesis treatments, these genotypes showed a minimal increase compared to the control (Figure S4), whereas Alidos exhibited a significant increase under PrAN2 (Figure S4). Moreover, the insensitive genotypes exhibited significant differences in florets per spikelet to pre-anthesis treatments, with more reduction under PrAN1 as compared to PrAN2 (Figure S4), except Brilliant which showed no difference in floret per spikelet under PrAN2 relative to the control (Figure S4), indicating genotype-specific robustness to light reduction.

In contrast, most of the spikelets of Kronjuwel, a sensitive genotype, differed significantly from control in both PrAN1 and PrAN2 (Fig. 7b), resulting in 69.44% reduction of florets per spikelet under PrAN1. Similarly, all sensitive genotypes exhibited significant decrease in number of florets per spikelet across entire main spike under PrAN1 (Figure S4). Majority of the sensitive genotypes showed significant reduction in florets at both top and bottom spikelets under PrAN2 compared to control (Figure S4). Furthermore, low light under PrAN1 significantly decreased the kernels per spikelet across whole main spike compared to control for all sensitive genotypes except Avalon and Intro, where reduction was preferred at basal and central spikelets (Figure S4). Under PrAN2, most of the sensitive genotypes also exhibited significantly reduced number of kernels on bottom spikelets in comparison to control (Fig. 7b, Figure S4), showing a prolonged sensitive phase. Moreover, Soissons and Sponsor showed highest reduction in number of kernels at 6 and 8 spikelet positions, respectively, under PoAN1 relative to control (Figure S5), and only one genotype (Kontrast) exhibited a significant decrease in number of kernels at 11 spikelet positions under PoAN2 (Figure S5).

These findings indicate that floret development is most sensitive to low light during the YA and TP subphase (PrAN1) both in insensitive and sensitive genotypes. Moreover, floret reduction does not appear to be spikelet-position specific in contrast to kernel abortion. Because basal positions were preferred for kernel abortion in the majority of genotypes under pre-anthesis light reduction.

## Discussion

In this work, we investigated the phase-specific and genotype-specific sensitivity to short-term light reduction in yield-related traits at plant, spike, and spikelet levels. In total, 16 genotypes, released between 1975 and 2013, were tested to demonstrate the genetic diversity in response to light. Our in-depth analyses provide (i) experimental confirmation of previous statistical approach; (ii) insights into the genotype-specific strategies of yield formation under fluctuating light environments; and (iii) phase-specific responses of floral and kernel development to low light.

### Realization of yield potential requires dynamic source–sink balance

Low light conditions decrease photosynthesis, leading to stunted growth and, potentially, delayed development, as plants produce less photosynthetic apparatus and less energy (Pao et al. 2019b). In the concept of source–sink balance (Gessler and Zweifel 2024), low light reduces carbon source strength and limits the development of sink organ (Fabre et al. 2020; Fang et al. 2024), which was observed under PrAN1 but to less extent under PrAN2 (Fig. 3h). This highlights the phase-specific dynamics of sink-demand before anthesis, as reported by Wang et al. (2026b). Although it is known that environmental conditions (Quintero et al. 2018; Shao et al. 2021) and genotype-specific factors (Fang et al. 2024; Wang et al. 2026a) affect floral and kernel development via their influences on pre-anthesis assimilates (source availability), we emphasize here that the contribution of pre-anthesis assimilates to kernel development is highly phase specific and affects not only the floret number but also the potential of grain filling (Fig. 5). In the stem elongation phase of wheat, sink activity is very strong since the plant needs to maintain the survival of tillers and floret development at the same time. Therefore, unfavorable environments that limit carbon source availability can have a dramatic consequence on the floret primordia (Fischer 1985; Reynolds et al. 2009; Philipp et al. 2018; Zhang et al. 2020), as shown in the reduced floret number (Fig. 4a). With fewer floret number and less kernels to fill, competition for assimilates between kernels was reduced at grain filling stage, allowing more carbon to be allocated to each remaining kernel. This is in accordance with the well-known trade-off between TKW and kernel number at the canopy level (Voss-Fels et al. 2019b; Lichthardt et al. 2020; Wang et al. 2025). We highlight genotype-specific and subphase-specific environmental effects on this trade-off (Fig. 5, Fig. 6) by showing the diverse response of TKW to the reduction in KpS under PrAN1 across the 16 studied genotypes (Fig. 5a). Low light in PrAN1 impacts ovary size and reduces kernel weight potential (Calderini et al. 2001; Hasan et al. 2011; Xie et al. 2015; Reale et al. 2017), which is a critical determinant of final kernel weight as it determines the upper limit for grain growth (Slafer et al. 2023). Most of the studied genotypes did not fully compensate the reduction in kernel number with an increase in kernel weight (Fig. 5a), suggesting an irreversible reduction of sink development in PrAN1. This stresses the fact that kernel weight potential is at least partly established during the stem elongation phase (Calderini et al. 2001). For example, some genotypes (eg Intro, Edward) were not able to compensate their loss in KpS by TKW (Fig. 5a), suggesting significant genotype-specific effects of pre-anthesis source-limitation (low light) on the potential of TKW. Therefore, in sensitive and less compensable genotypes, development of sink strength (defined by the number of kernels and their potential size) emerges as a critical compensation- and yield-limiting factor under PrAN1 during YA-TP subphase (Fig. 6). Although the mechanisms underlying these genotypic differences remain unknown, they may be related to carbohydrate storage and mobilization during this critical period. However, carbohydrate storage and mobilization were not assessed in this study owing to the large number of genotypes (16), treatments (5), and replications (20 plants per genotype per treatment), but it will be interesting in the future to test the hypothesis that insensitive cultivars more efficiently convert stored carbohydrates into sugars during YA-TP, thereby achieving a better dynamic source–sink balance. Considering year of release in Table 1 with the relative change in KpS and TKW (Fig. 5) together, our results suggest that source–sink balance during YA-TP subphase has not yet been selected by breeders and could be targeted for manipulation, for enhancing plant acclimation to environmental fluctuations (ie low light) and to improve grain yields in wheat breeding programs.

Reduced light intensity (source availability) during the post-anthesis period is known to decrease TKW (Dong et al. 2014). While this effect was not observed at the whole-plant level (Fig. 3c), it did impact total kernel weight of main spike (Fig. 3k). This suggests that: (i) the source–sink dynamics of the main spike and tiller spikes may not be synchronized, and (ii) the critical period for source availability and grain filling at the whole-plant level may be shorter than previously reported in the literature.

### Differential resource allocation and remobilization across the spike result in spikelet position specific kernel abortion under low light

Consistent with previous findings, kernel number is particularly sensitive to changes in crop growth and development during the stem elongation phase, more so than in any other phase (Slafer 2003; Fischer 2011; Reynolds et al. 2022). Specifically, reduced light intensity during the YA-TP and HD-AN phases significantly decreased the number of florets per spike (Jia et al. 2021), leading to a marked decline in kernels per spike (Fig. 3h, i). This reduction could be attributed to the autophagy of florets at distal or basal spikelet positions on the main spike or on tillers (Fig. 7), which were less developed or experienced delayed development compared to florets in central spikelets. Why the YA-TP subphase is uniquely vulnerable to light reduction, while other subphases show little or no autophagy, remains unclear. To which degree and at which spikelet positions the development of floret primordia is limited by the pre-anthesis source availability is certainly genotype specific and there are clear interactions between genotype, sensitive phase, and spikelet positions (Fig. 7, Figure S4). In general, central spikelets have the highest kernel number, followed by the apical spikelets and then the basal spikelets (Guo and Schnurbusch 2015; Sun et al. 2024). In insensitive genotypes, the central and basal spikelets showed the strongest degree of floret reduction under PrAN1 (Fig. 7a), indicating preferential allocation of assimilates between spikelets. Indeed, central spikelets demonstrate a higher ability to compete for assimilates for survival compared to basal spikelets (Backhaus et al. 2023; Sun et al. 2024). Under PrAN1 and PrAN2, it is noteworthy that insensitive genotypes exhibit a reduced abortion rate. For instance, in the insensitive genotype Nimbus, the higher abortion rate under control conditions (compared to PrAN1 and PrAN2) suggests that these plants may potentially face a post-anthesis source limitation for grain development. The physiological mechanism behind abortions could involve the accumulation of abscisic acid and jasmonate acid induced by low sucrose content. This accumulation is known to suppress the development of floret primordia into fertile florets in basal spikelets (Sun et al. 2024). In contrast, sensitive genotypes exhibited a more uniform reduction in florets and an increased abortion rate across all spikelets (Fig. 7b), suggesting potential genotypic differences in the mechanisms of sugar distribution within the spike or indicating that the entire spike is a weaker sink. Additionally, the increased abortion rate under PrAN1 and PrAN2 in the sensitive genotype Kronjuwel (Fig. 7b) might result from low-light effects on pollen fertility or other processes related to fertilization and fruiting efficiency. Also, distal florets within a spikelet are inadequately connected to the vascular systems that supply nutrients and water (Flavell 2023). Therefore, they are also prone to abortion. Whether insensitive genotypes suppress or delay floret autophagy under reduced light conditions remains unknown and warrants further study. Floret autophagy is likely triggered by the reduced availability of photoassimilates or failure to convert stored carbohydrates into sugars and the inadequate transport of sugars and other essential nutrients from the leaves and stem into the spike (Ghiglione et al. 2008; Reynolds et al. 2022). Additional experiments assessing the expression of key photosynthesis-related genes and autophagy markers during PrAN1 in sensitive and insensitive would help to confirm the exact mechanism. Altogether, our results indicate the different physiological regulations of carbon assimilates in the processes of floret development and floret abortion.

### Discrepancies between statistical approach and experimental findings

Field crops exposure to fluctuations in environmental variables during critical developmental phases can have significant positive or detrimental effects on final grain yield, especially under the context that climate extremes occur more frequently and repeatedly (Suarez-Gutierrez et al. 2023; Vautard et al. 2023). However, investigating the short-term impacts of environmental variables on the formation of yield components through manipulation of these variables, both under controlled and field conditions, is highly labor intensive (Xu, 2016). This endeavor necessitates (i) employing multiple levels of a specific environmental variable and (ii) assessing the effects of these variables across distinct developmental subphases. Consequently, it is implausible to experimentally evaluate the stage-specific responses of yield components to environmental fluctuations during generative development across numerous genotypes. Therefore, experimental investigations into the short-term environmental impacts on yield component formation have been restricted to a limited range of environmental variables and assessed in a small number of genotypes at specific developmental stages. In this work, we experimentally proved the concept that comprehensive field phenotyping using standardized protocols in multienvironmental field experiments can effectively identify the cultivar-specific sensitivities to short-term environmental fluctuations (Sabir et al. 2023a). In our experiment, most of the tested genotypes (ie Brilliant, Kobald, Ibis, Avalon, Sponsor, Kronjuwel, Soissons, and Edward) showed consistent responses as indicated by the statistical approach found in Sabir et al. (2023a), although some inconsistencies were observed. The following factors in the current experiment could be the reasons for this inconsistency: (i) Here, only kernels on the main spike were considered for KpS, whereas the statistical approach considered a mean KpS of the whole plot with spikes from tillers; (ii) we applied treatments based on genotype-specific phenology (Figure S2). The fact that phenological length across all genotypes varied (Fig. 2) and this cannot be considered in the statistical approach makes the results not directly comparable; (iii) to maintain comparability between treatments and between genotypes, the duration of treatments was not genotype specific; (iv) potential noise and false significance in the statistical approach and the algorithm for peaks detection (Figures S1, S2). Despite some discrepancies, our experiment confirms the critical stage (YA-TP) identified through statistical analysis, which is shorter than those observed in previous studies using prolonged shading treatments, such as 28 days at 55% light reduction (Fischer et al. 2024). Additionally, we emphasize that the effects of PrAN1 on KpS were not correlated with the length of any phenological subphase (Fig. 2, Fig. 5), indicating that the differences in sensitivity are not simply a consequence of genotypic variations in phenological duration. Furthermore, we discovered the phase-specific compensability (Fig. 5), an aspect in the trade-off between KpS and TKW. Since this trade-off observed in the experiment (Fig. 6) differs from that in the field observation (Figure S6), it could be further hypothesized that there exist stage-specific, environmental variable-specific, and genotype-specific compensability. Our findings provide 2 key insights for wheat breeders: (i) the potential to target the YA-TP subphase to select insensitive genotypes with reduced abortion rates, and (ii) the opportunity to identify genotypes with enhanced compensatory ability through increased TKW, thereby improving yield stability. However, both approaches require labor-intensive phenotyping, which may be impractical for breeding programs. Interestingly, we found no correlation between the year of release and either the sensitivity of KpS or the compensatory ability of TKW (Table 1, Fig. 5, and Fig. 6), suggesting that these traits were not actively selected. This conclusion, however, is based on a small sample of 16 genotypes. For plant physiologists, this provides an exciting opportunity to explore the physiological mechanisms of insensitive genotypes using omics approaches.

## Materials and methods

### Plant materials

To study the genotype and subphase specific sensitivity to light intensity, 16 wheat (*Triticum aestivum*) genotypes were selected from a panel of 220 winter wheat genotypes used in our previous studies (Voss-Fels et al. 2019b; Lichthardt et al. 2020; Sabir et al. 2023a; Manntschke et al. 2025; Wang et al. 2025). These genotypes were grown in multiple environment field trials from 2015 to 2017, and their sensitivities to short-term light fluctuations were estimated by using a statistical approach (Sabir et al. 2023a). To identify the sensitive phenological periods, a peak detection algorithm (Figure S1, see also the section “Statistical analyses”) between thermal time and the significance of the effects of global radiation on KpS (–ln *P*_value_) across 220 genotypes was applied. For most genotypes, a pre-anthesis period (at 100 °Cd before HD) and a post-anthesis period (at 240 °Cd after HD) were identified as most sensitive to light fluctuations for KpS and these periods were chosen to test subphase specific responses in the current study. The response of the 16 selected genotypes during the identified sensitive subphases was visualized, and they were categorized into 4 contrasting sensitive groups (Table 1, Figure S2).

### Growth conditions and experimental design

Seeds were sown in potting soil (Potgrond H, Klasmann-Deilmann GmbH, Geeste, Germany) in trays under greenhouse conditions (14-h day-length, 20/15 °C, day/night) for 14 days to obtain 210 seedlings of 2 to 3-leaf stage per genotype for the experiment. After that, seedings were transferred to a growth chamber to vernalize at ∼4 °C (12-h day-length) for 63 days. Vernalized seedlings were acclimatized in a greenhouse at 15/15 °C (photoperiod 12 h) for 7 days. After that, each seedling was transferred to a plastic box (7.5 cm × 4 cm, 25 cm in depth, with plant density similar to field conditions) and grown under greenhouse conditions (April 4, 2022/16-h day-length, 20/15 °C day/night temperature). During the experiment, all plants were manually irrigated every 2 to 3 days to maintain the 85% to 95% field capacity and provided with 0.1% NPK-fertilizer Ferty Mega2 (PLANTA, Regensburg, Germany) as required. Since the light intensity is an important factor in the experiment, each genotype in the greenhouse was arranged as a microplot canopy with 54 border plants to reduce the variation in lateral light interception in the experiment. To determine progress of developmental subphases (see the section “Determination of phenological subphases” below), 2 plants per genotype were dissected every second day. In total, 30 plants per genotype were reserved for dissection until anthesis. When a genotype achieved the WA stage, plants of this genotype were moved to a growth chamber (photoperiod 18:6 h; temperature, 20/14 °C) to acclimatize to medium constant light intensity (∼26 mol m^−2^ d^−1^ photosynthetic photon flux density) for 4 to 7 days prior to light treatments. As the number of days to reach WA differed between genotypes, plants of each genotype were moved to the growth chambers on different days with a maximal difference between earliest and latest genotypes of 18 days.

Two days after reaching the GA stage, light treatments were applied across 4 distinct subphases: (i) a light-sensitive pre-anthesis subphase between YA and TP (YA-TP; PrAN1); (ii) a light-insensitive pre-anthesis subphase between HD and AN (HD-AN; PrAN2); (iii) a light-sensitive post-anthesis subphase between PGF and the beginning of grain filling (GF) (PGF-GF; PoAN1); and (iv) a light-insensitive post-anthesis subphase at the beginning of GF (PoAN2). The total duration of these treatments was approximately 7 days (with a thermal time accumulation of 19 °Cd per day) and spanned these subphases (Fig. 1).

For the light treatments, low light intensity (5 mol m^−2^ d^−1^) was applied during the YA-TP subphase, followed by high light intensity (62 mol m^−2^ d^−1^) during the HD-–AN subphase PrAN1. The order of light treatments was reversed in PrAN2. Similarly, during post-anthesis, low light was applied in the sensitive PGF-GF subphase, followed by high light during the GF subphase in PoAN1, with the reverse order in PoAN2. A control group was exposed to high light throughout the entire experimental period, representing conditions closest to those required for the realization of potential kernel number.

Out of the 126 plants available before treatment initiation, 100 of the most homogeneous plants (main shoots and all tillers) per genotype were selected for the experiments (10 plants per treatment, with 2 replicates in separate growth chambers). In the growth chamber, 10 plants per genotype per treatment were arranged in a split-plot design with light intensity randomized as main plot and genotype as sub-plot. The experiment was replicated 2 times in 2 growth chambers. After light treatments, all plants during the daytime were kept in the growth chamber under constant light intensity (62 mol m^−2^ d^−1^) till maturity (harvest). Over the entire period of spike and grain development, all plants received the same light integral in 4 light treatments except for the control. The different light levels were obtained in the growth chamber by adjusting the distance of plants from the light source (Pao et al. 2019a, 2020).

### Determination of phenological subphases

Since the treatment started at genotype-specific phenological subphases, the phenological development of each genotype was monitored during the whole experiment. To detect the WA stage (lemmas of florets 1 and 2 completely enclose stamens and other structures) and GA stage (glumes cover all but the tips of florets) (Kirby and Appleyard 1984), 2 plants of each genotype were dissected every 2 days under a stereomicroscope (Stemi 2000-c, Carl Zeiss Micro Imaging GmbH, Göttingen, Germany) after vernalization. For TP (Z49, first awns visible; Zadoks et al. 1974), HD (Z55, 50% of spikes visible; Zadoks et al. 1974), and AN (Z65, 50% of spikes with anthers; Zadoks et al. 1974), the day of onset was recorded as the point at which 50% of the plants had reached the respective stage, and the numbers of days to reach each stage for each genotype were recorded as subphase durations. Thermal time (°Cd) was used to quantify the duration of each subphase and was calculated as the accumulated averages of the daily recorded maximal and minimal temperatures, assuming a base temperature of 0 °C (Parent et al. 2019).

### Agronomic and yield traits

After harvest, main spikes of 10 plants per genotype per treatment per repetition were analyzed in detail to determine the total spikelet number per spike, number of florets per spikelet, and kernel number at each spikelet. To determine the distribution of florets and kernel number at each spikelet position, each spikelet along the rachis was opened one by one to count visible florets and kernels. Spikelet position was numbered from 1 at the base to *n* at the terminal spikelet of the main spike, and total florets and kernels on main spike were determined. These data were used to calculate total number of florets, kernels, total kernel weight per main spike, filling rate per main spike, and per spikelet (total kernels/total florets), and abortion rate per main spike, and per spikelet (1 – filling rate). Kernels from all 10 plants per genotype, treatment, and replication were combined and analyzed by a MARVIN Seed Analyser (https://www.marvitech.de/) for TKW (g), kernel width (mm) and kernel length (mm). Furthermore, total shoot dry mass per plant (g), number of spikes per plant, main spike kernels weight (g), tillers kernel weight (g), and total grain yield per plant (g) per genotype, treatment, and replication were recorded.

### Statistical analysis

To detect the genotype-specific sensitive phenological periods, a dataset consisting of –ln *P*_value_ and coefficients of 220 genotypes of KpS and global radiation was used, which can be accessed from an online data repository (Sabir et al. 2023b). At first the dataset was systematically sorted by thermal time in ascending order, and a threshold value of 1.5 to mitigate noise interference was applied. The peak detection algorithm functioned by iteratively scanning the dataset, identifying successive local maxima, and defining their boundaries based on neighboring data points.

For the growth chamber experiment, outliers in all of the traits were removed using Cook's method (Cook 1977). First, the following linear regression model was fitted:


$$Y_{ij}\;=\;\mu \;+\;T_{j}\;+\;G_{i}\;+\;\varepsilon_{ij},$$

where *Y*_ij_ is the phenotypic observation of the *i*th genotype in *j*th treatment, *μ* is the general mean of *Y*, *T* is the factorial level of *j*th treatment, *G* represents the effect of *i*th genotype, and *ε*_ij_ is the residual error.

After fitting the model, each Cook's distance (*D*_i_) was computed for each observation *Y*_ij_ using the cooks.distance() function. If an observation had a *D*_i_ at least 7 times larger than the mean *D*_i_, it was considered an outlier and removed from the dataset. This procedure removed between 0% and 1.68% of the recorded values, depending on the traits.

By using the lme() function in *nlme* package in R, traits were analyzed using a linear mixed-effects model, with genotype, treatment, and their interaction included as fixed effects, while 2 growth chambers were incorporated as an additional fixed blocking factor to control for systematic variation among chambers, with 10 repeated observations (10 individual plants per genotype per chamber) nested within chambers as the random factor. Based on this model, estimated marginal means were calculated by using the function emmeans() in the *emmeans* package in R. To compare the genotype-specific response to treatment (Fig. 4), post hoc comparisons were conducted separately within each genotype. Pairwise comparisons among treatment levels within each genotype were adjusted for multiple testing using the Sidák correction to control the family-wise error rate, with a significance threshold of *P* ≤ 0.05. To determine the effect at treatment level (Fig. 3), we performed split-plot 2-way ANOVA on plant and main spike phenotypic data using sp.plot() function in the “*agricolae*” package (de Delgado 2019), considering light treatment as main plots and genotype as sub-plot. LSD.test() was used for post hoc multiple comparisons, at the 5% significance level. The mean differences between treatments at each spikelet position was identified by *t*-test. All of the analysis was performed in R environment (R Core Team 2021).

## Supplementary Material

kiag277_Supplementary_Data

## Data Availability

All primary data are available upon request.
