# Supplementary material for: Cultivar- and phase-specific effects of light on kernel number and compensation via kernel weight in wheat
Source: Plant Physiol. 2026 Jul 23;201(4):kiag277. doi: 10.1093/plphys/kiag277 (PMC13524324; doi:10.1093/plphys/kiag277)
Supplement: Supplementary file 1 [file kiag277_supplementary_data.pdf]

***Supporting Information (Phase-specific effects of light on of kernel number and compensation via kernel weight in wheat depend on cultivars)***

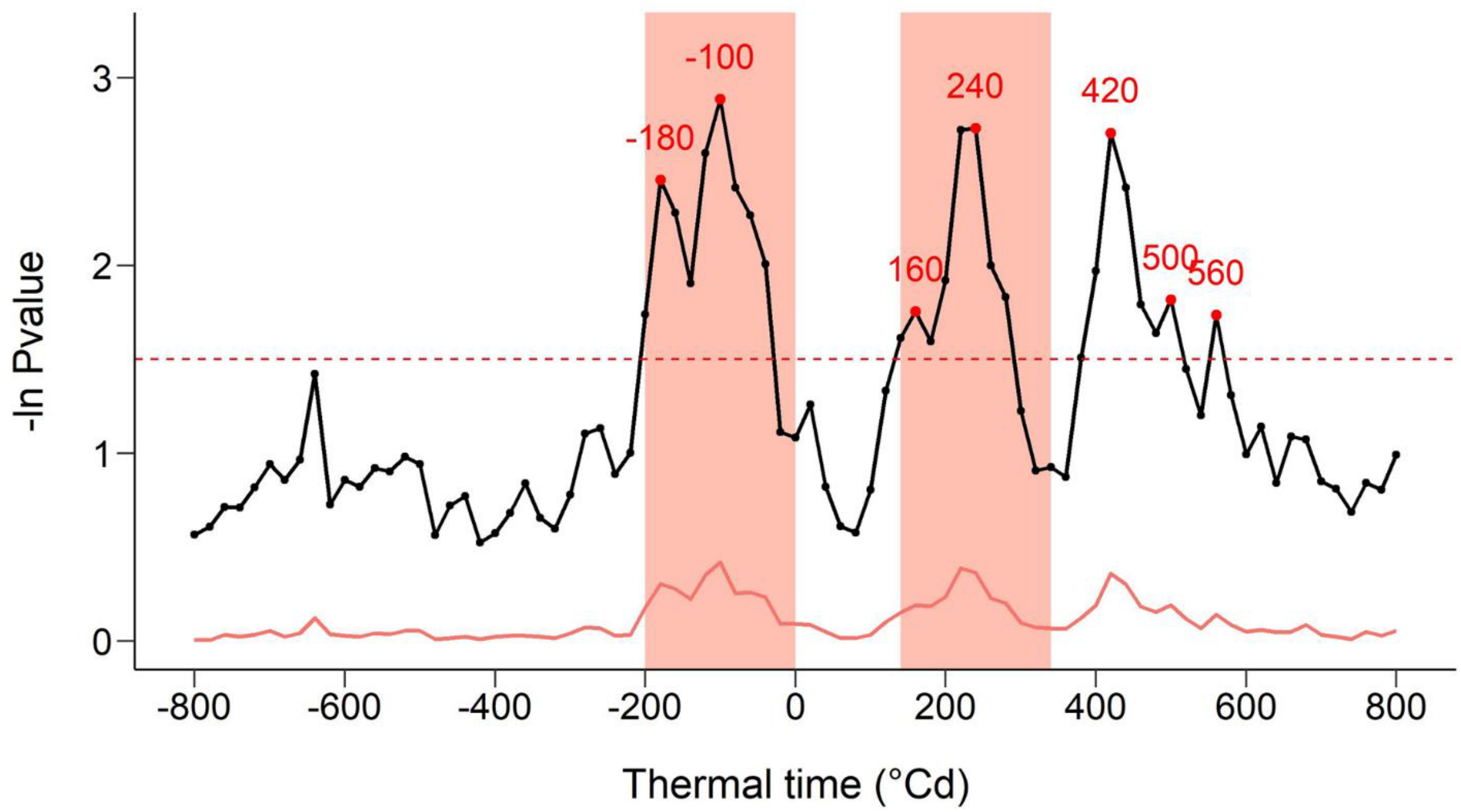


***Figure S1:*** Detection of phenological sub-phases sensitive to fluctuating light intensity across 81 time windows before and after heading (HD, thermal time = 0°Cd) on kernels per spike (KpS). Solid black line represents mean -ln *P*value of 220 genotypes at each time-window against thermal time (x-axis) of KpS development. The time-windows with a peak are marked with the thermal time (°Cd) (red color) and regarded as phenological sub-phases sensitive to light intensity. Two most sensitive phenological sub-phases were included in this study (pink background). Solid red line represents the proportion of cultivars with ≥ 3 –ln *P*value out of total 220 cultivars within each time-window. Dotted red line depicts the threshold = 1.5.

***(Starting from next page)***

***Figure S2***. Visualization of phenological sub-phases sensitive to fluctuating light intensity across 81 time windows and their impact on kernels per spike (KpS) in the 16 selected genotypes. -ln Pvalue (black solid line) and coefficients (blue dotted line) of 16 selected genotypes in 81 time-windows before and after heading (HD, thermal time = 0°Cd). Two identified sensitive sub-phases with red background are shown at 100 °Cd before heading and at 240 °Cd after heading. Red dashed line represent the threshold of significance. In this study, the duration of genotype-specific treatment for low light is highlighted with skyblue background. Purple represents the overlap of these the time-windows.

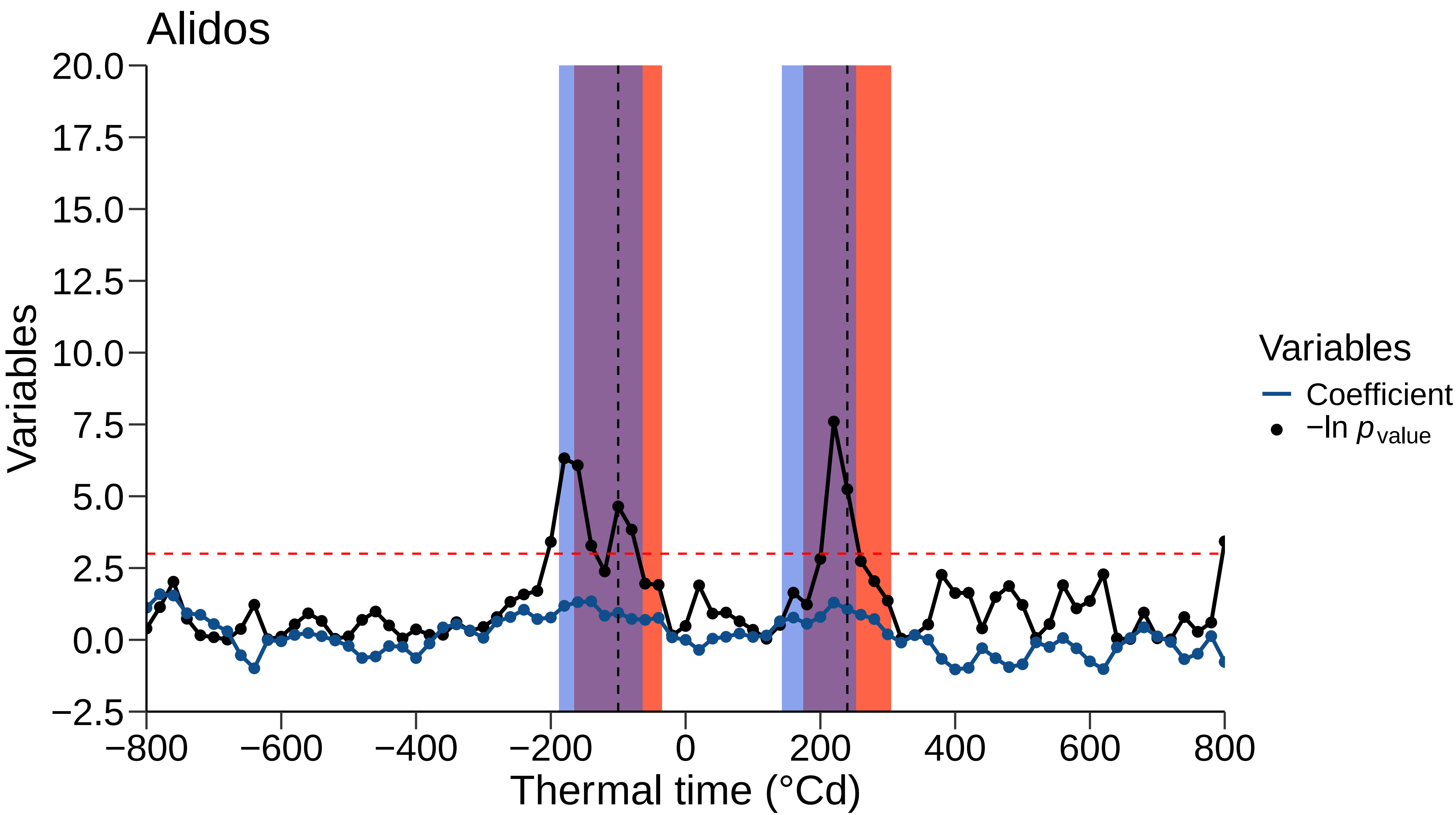
Alidos
20.0
17.5
15.0
12.5
10.0
7.5
5.0
2.5
0.0
−2.5
Variables
−800
−600
−400
−200
0
200
400
600
800
Thermal time (°Cd)
Variables
Coefficient
−ln p value

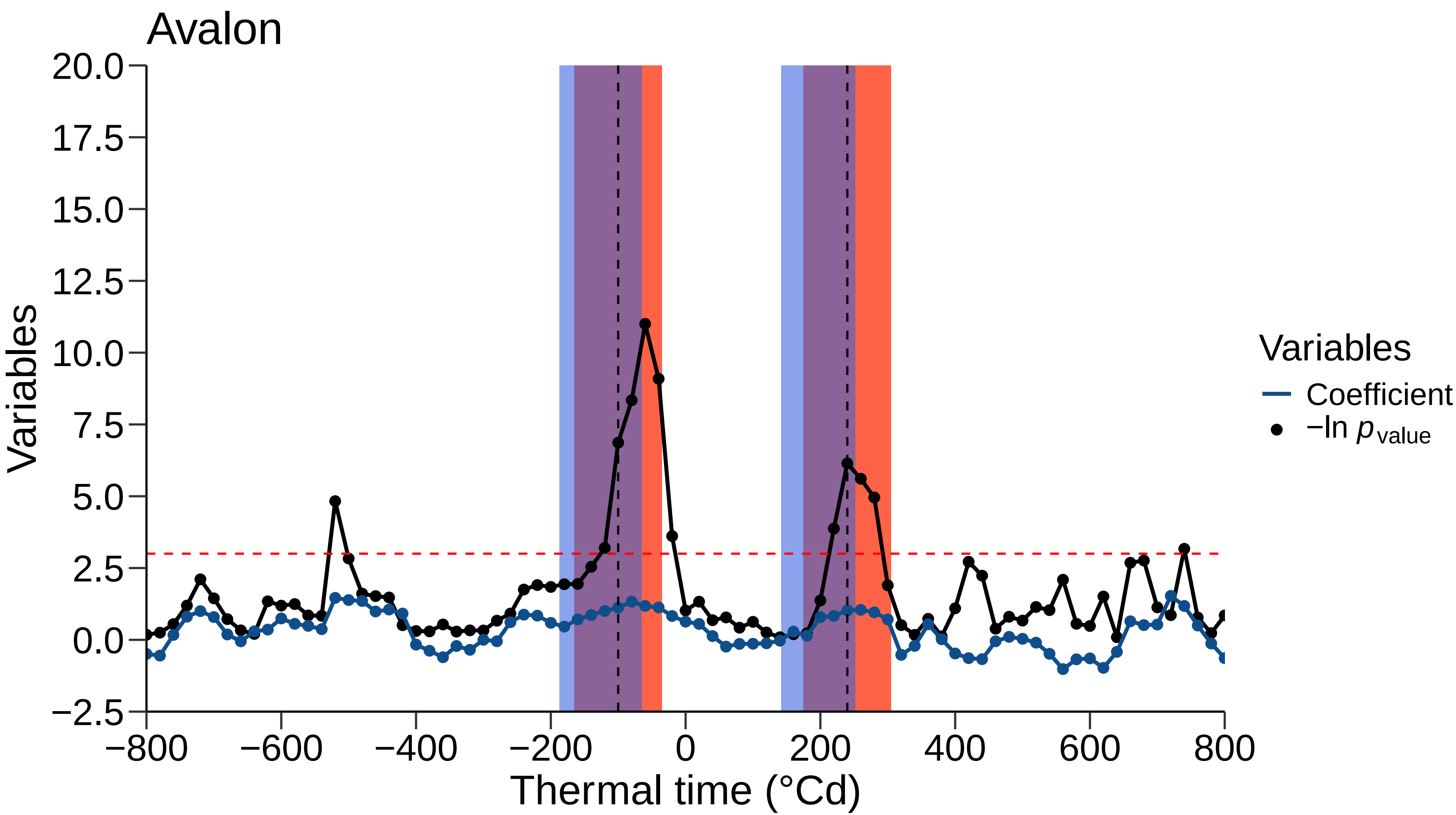

Avalon
20.0
17.5
15.0
12.5
10.0
7.5
5.0
2.5
0.0
−2.5
Variables
−800
−600
−400
−200
0
200
400
600
800
Thermal time (°Cd)
Variables
Coefficient
$-\ln p_{value}$

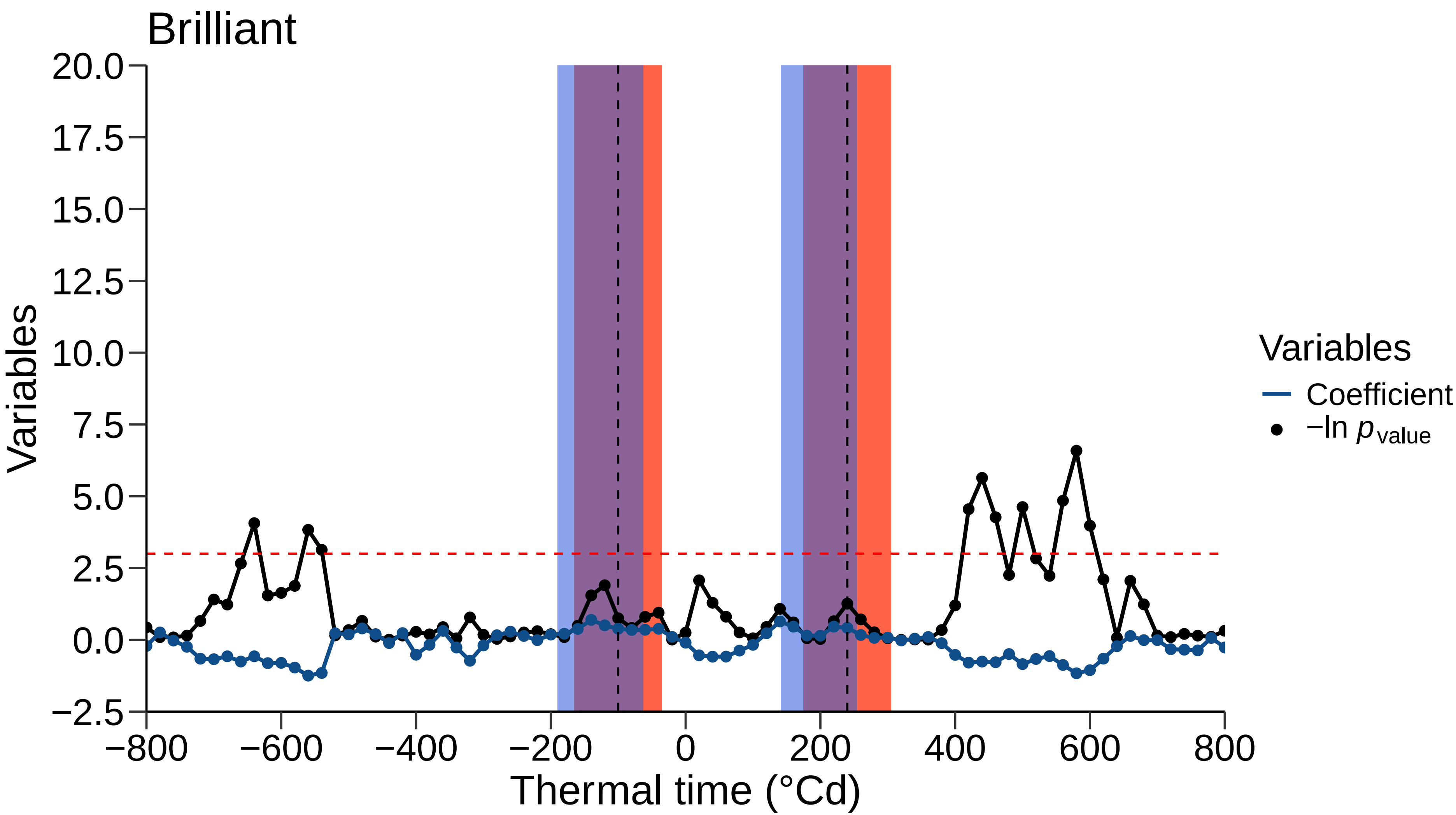

Brilliant
20.0
17.5
15.0
12.5
10.0
7.5
5.0
2.5
0.0
−2.5
Variables
−800
−600
−400
−200
0
200
400
600
800
Thermal time (°Cd)
Variables
Coefficient
−ln p value

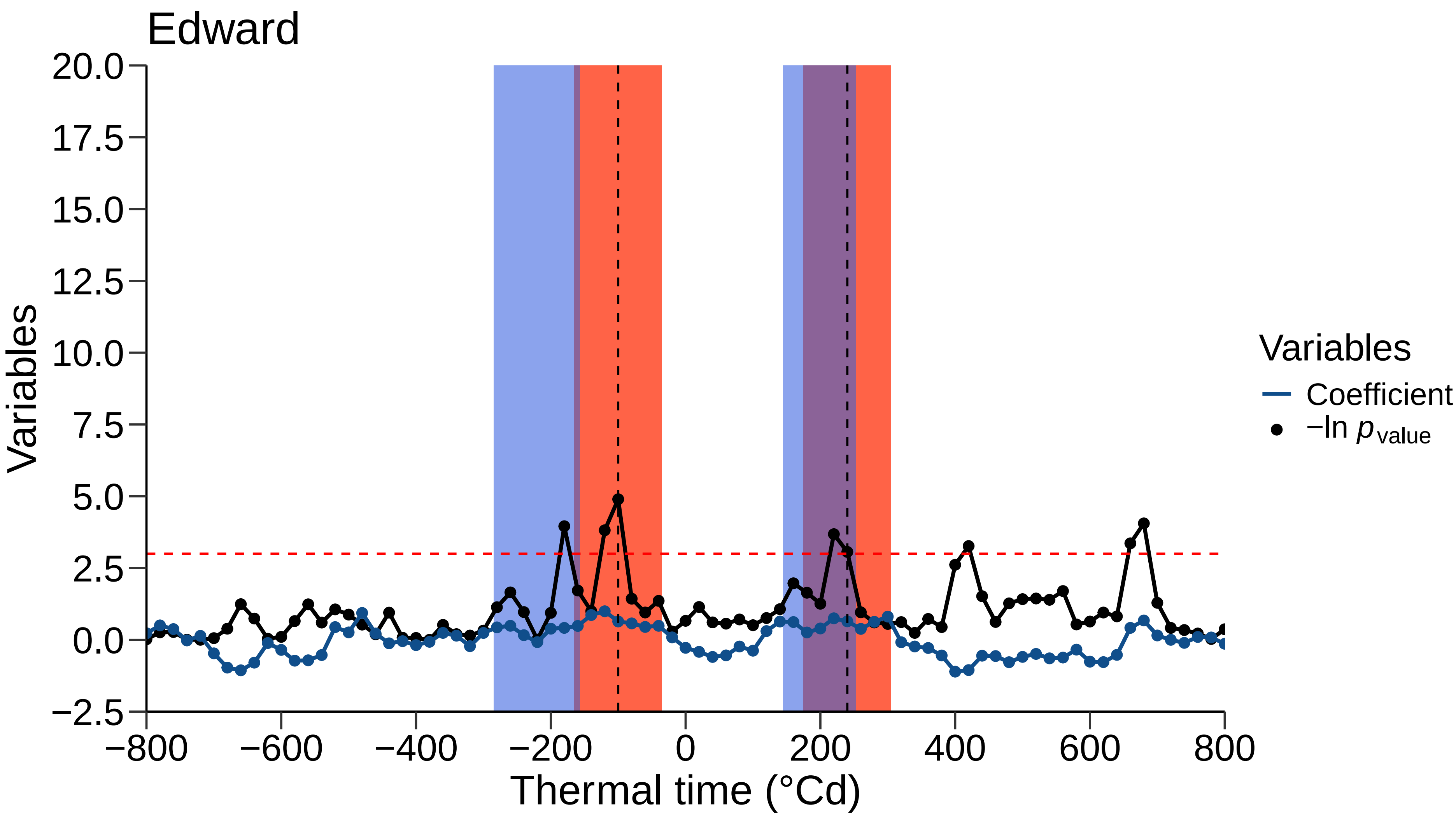
Edward
20.0
17.5
15.0
12.5
10.0
7.5
5.0
2.5
0.0
−2.5
Variables
−800
−600
−400
−200
0
200
400
600
800
Thermal time (°Cd)
Variables
Coefficient
−ln $p_{value}$

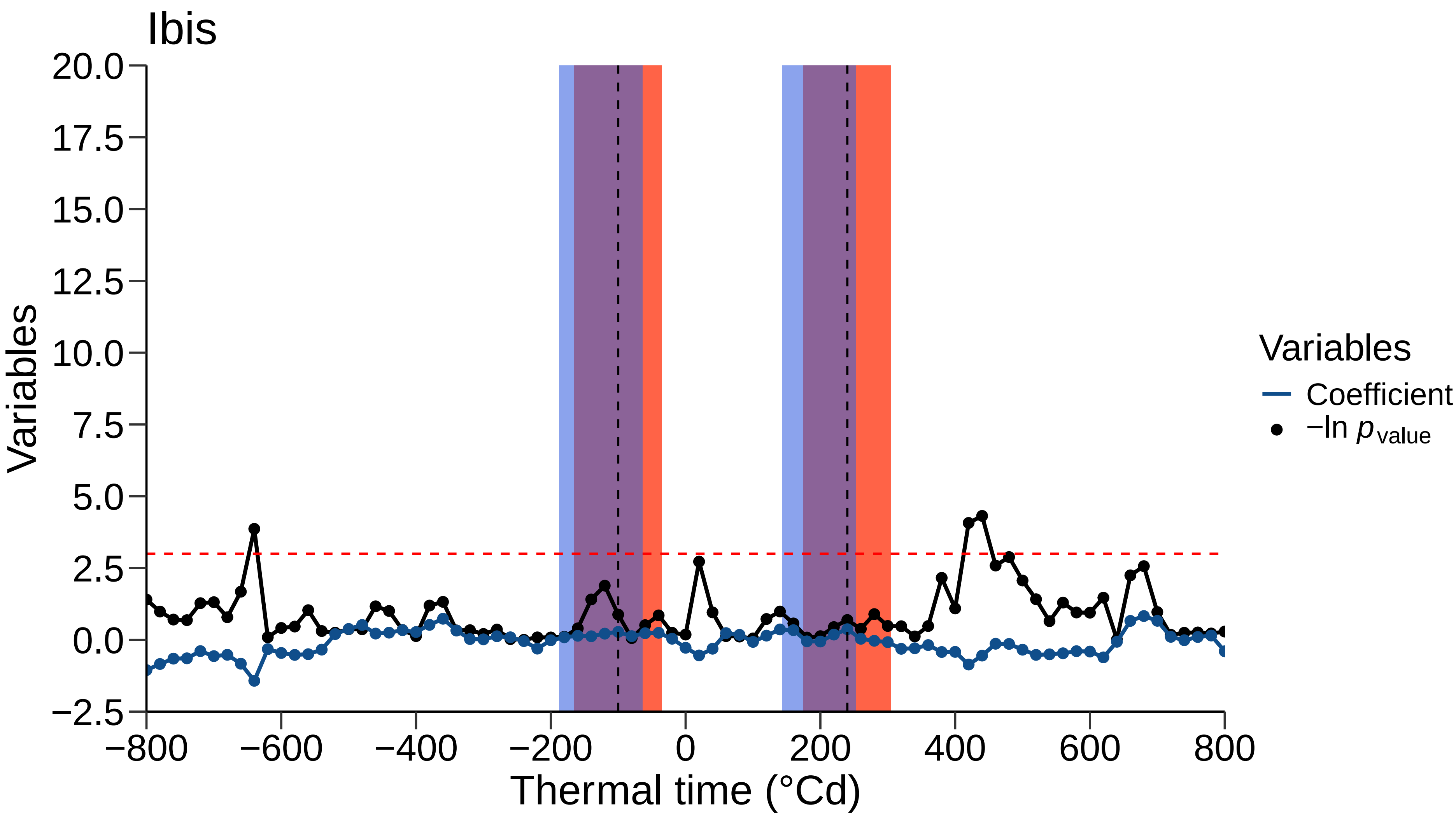

Ibis
20.0
17.5
15.0
12.5
10.0
7.5
5.0
2.5
0.0
−2.5
Variables
−800
−600
−400
−200
0
200
400
600
800
Thermal time (°Cd)
Variables
Coefficient
−ln $p_{value}$

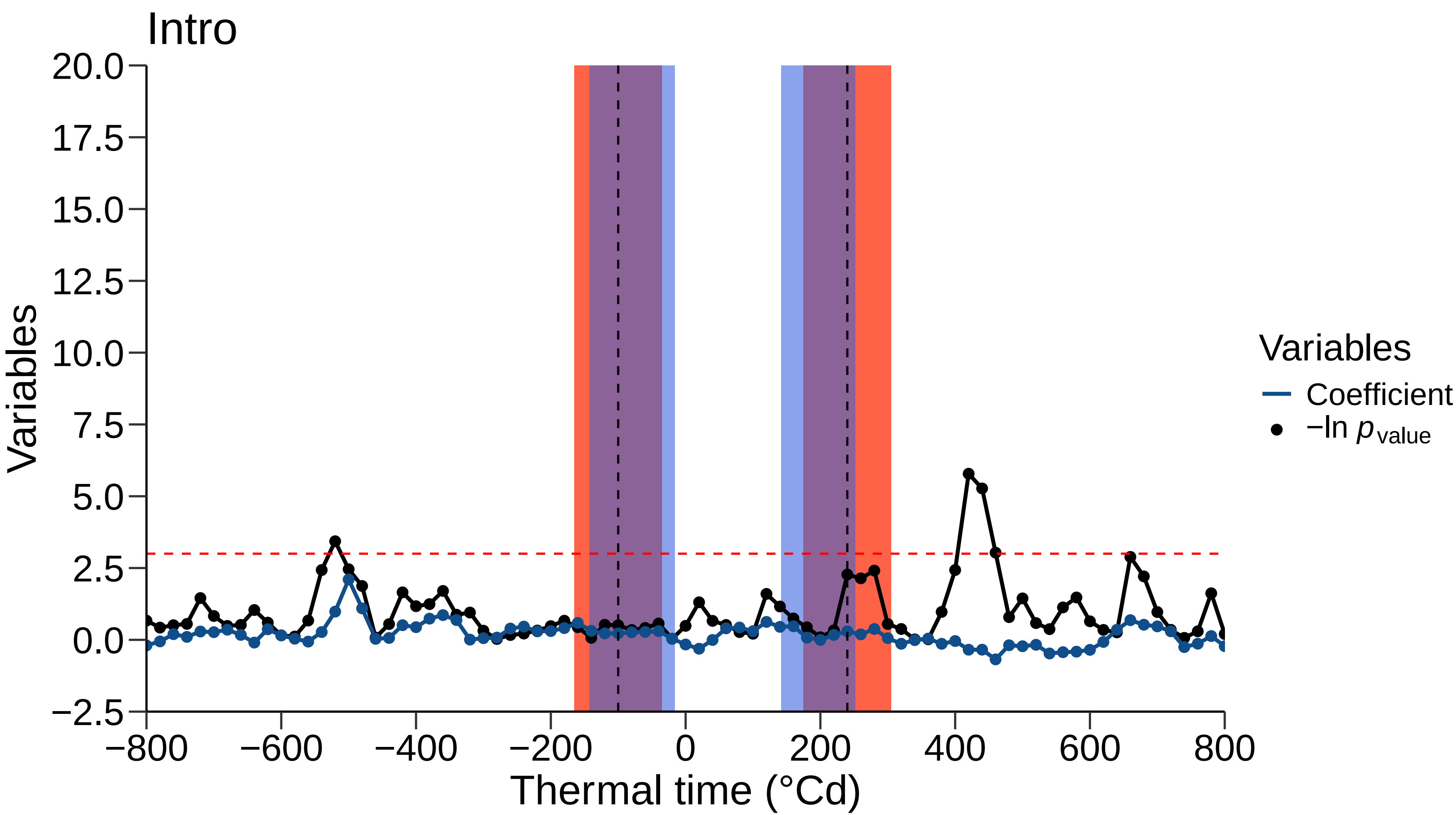

Intro
20.0
17.5
15.0
12.5
10.0
7.5
5.0
2.5
0.0
−2.5
Variables
−800
−600
−400
−200
0
200
400
600
800
Thermal time (°Cd)
Variables
Coefficient
−ln $p_{value}$

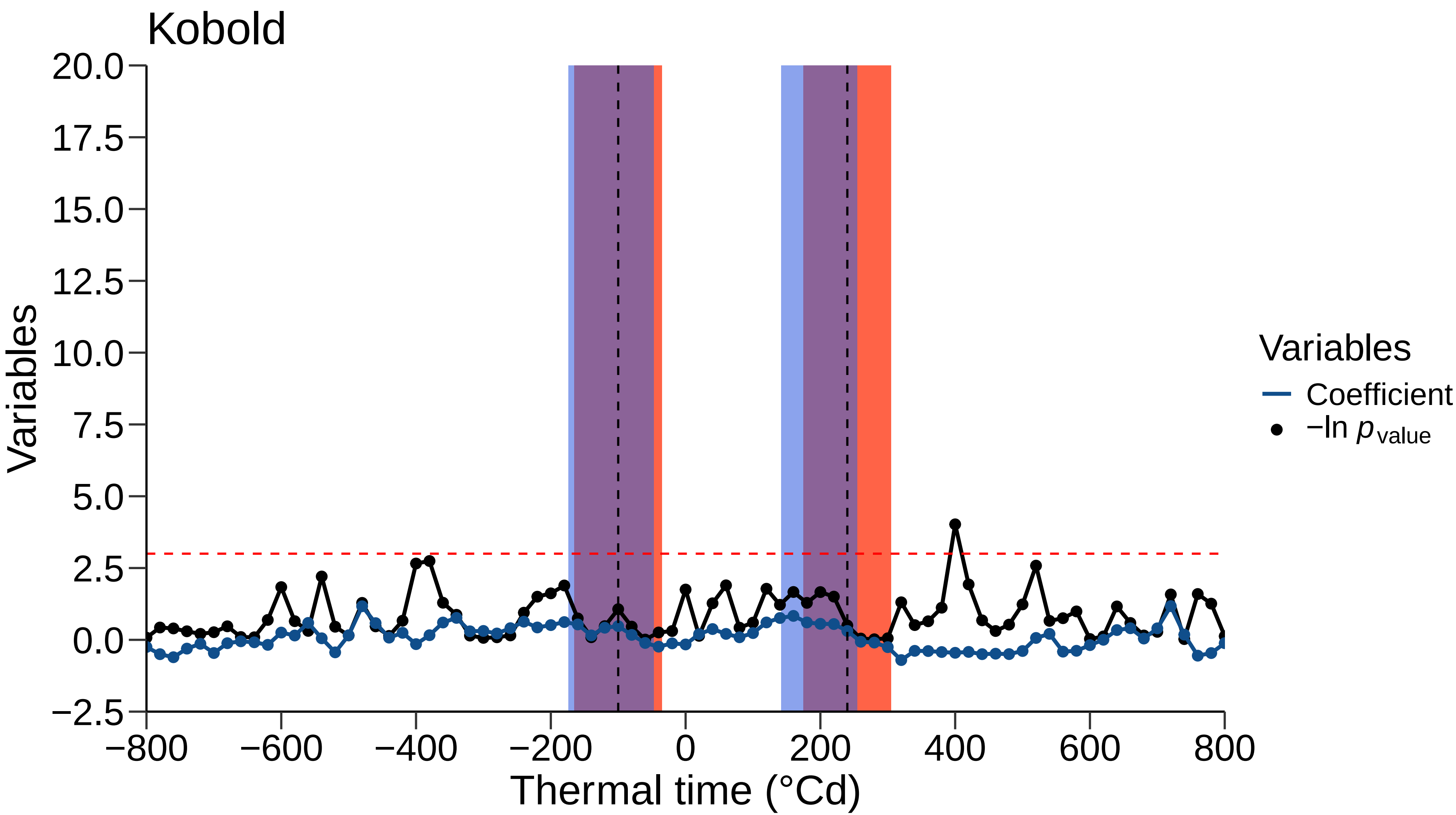

Kobold
20.0
17.5
15.0
12.5
10.0
7.5
5.0
2.5
0.0
−2.5
Variables
−800
−600
−400
−200
0
200
400
600
800
Thermal time (°Cd)
Variables
Coefficient
−ln $p_{value}$

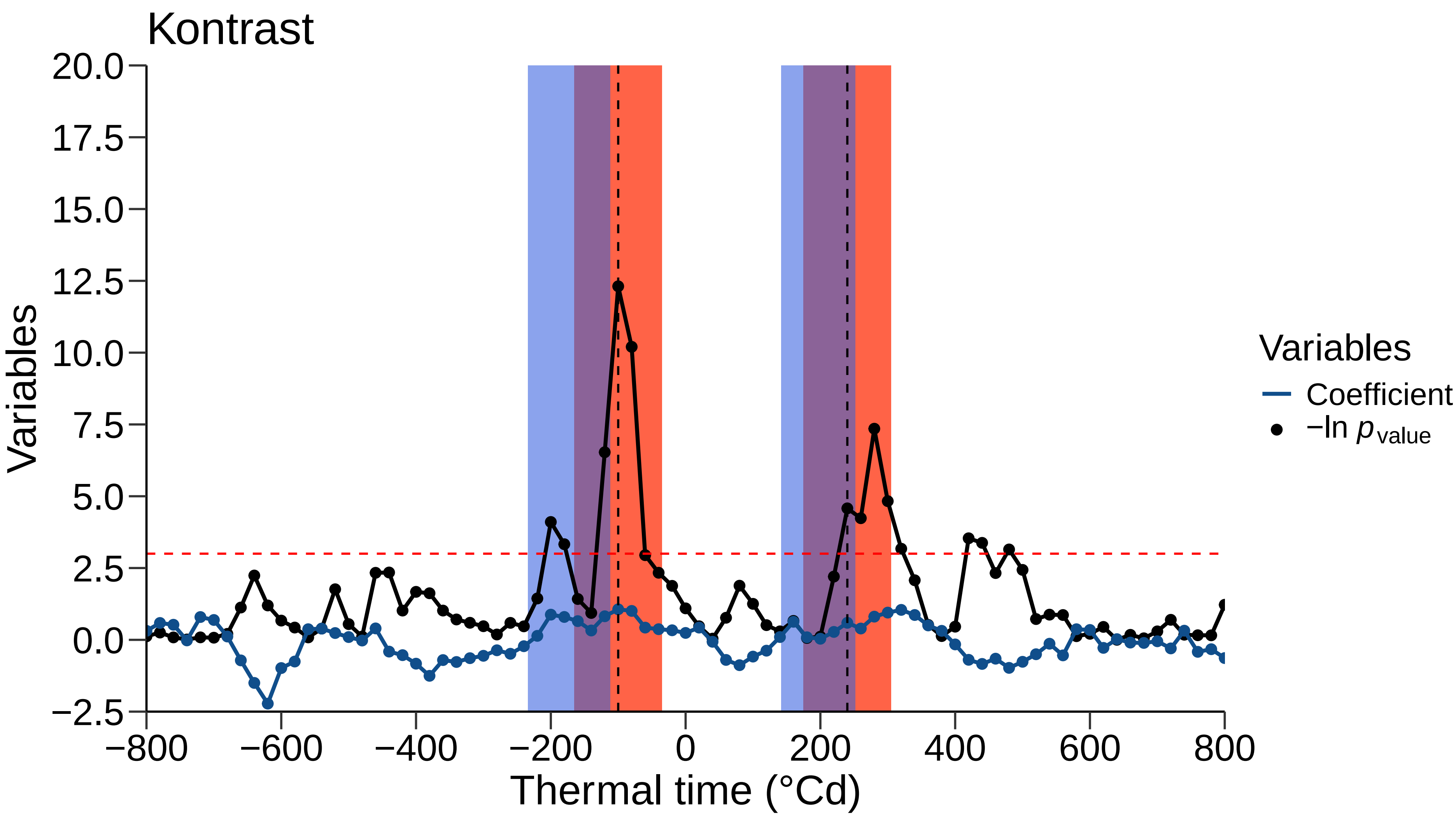

Kontrast
20.0
17.5
15.0
12.5
10.0
7.5
5.0
2.5
0.0
−2.5
Variables
−800
−600
−400
−200
0
200
400
600
800
Thermal time (°Cd)
Variables
Coefficient
−ln $p_{value}$

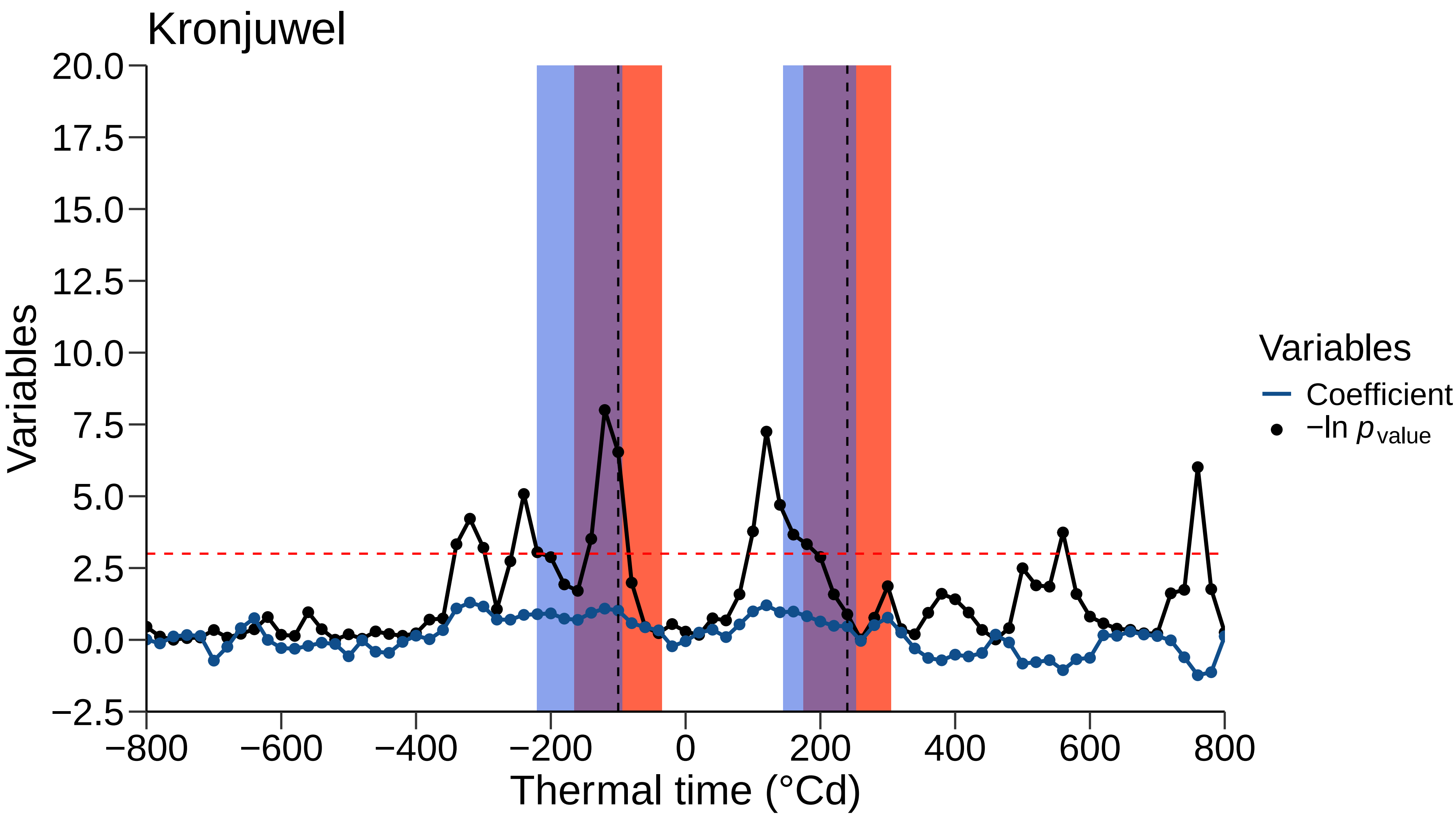

Kronjuwel
20.0
17.5
15.0
12.5
10.0
7.5
5.0
2.5
0.0
−2.5
Variables
−800
−600
−400
−200
0
200
400
600
800
Thermal time (°Cd)
Variables
Coefficient
−ln $p_{value}$

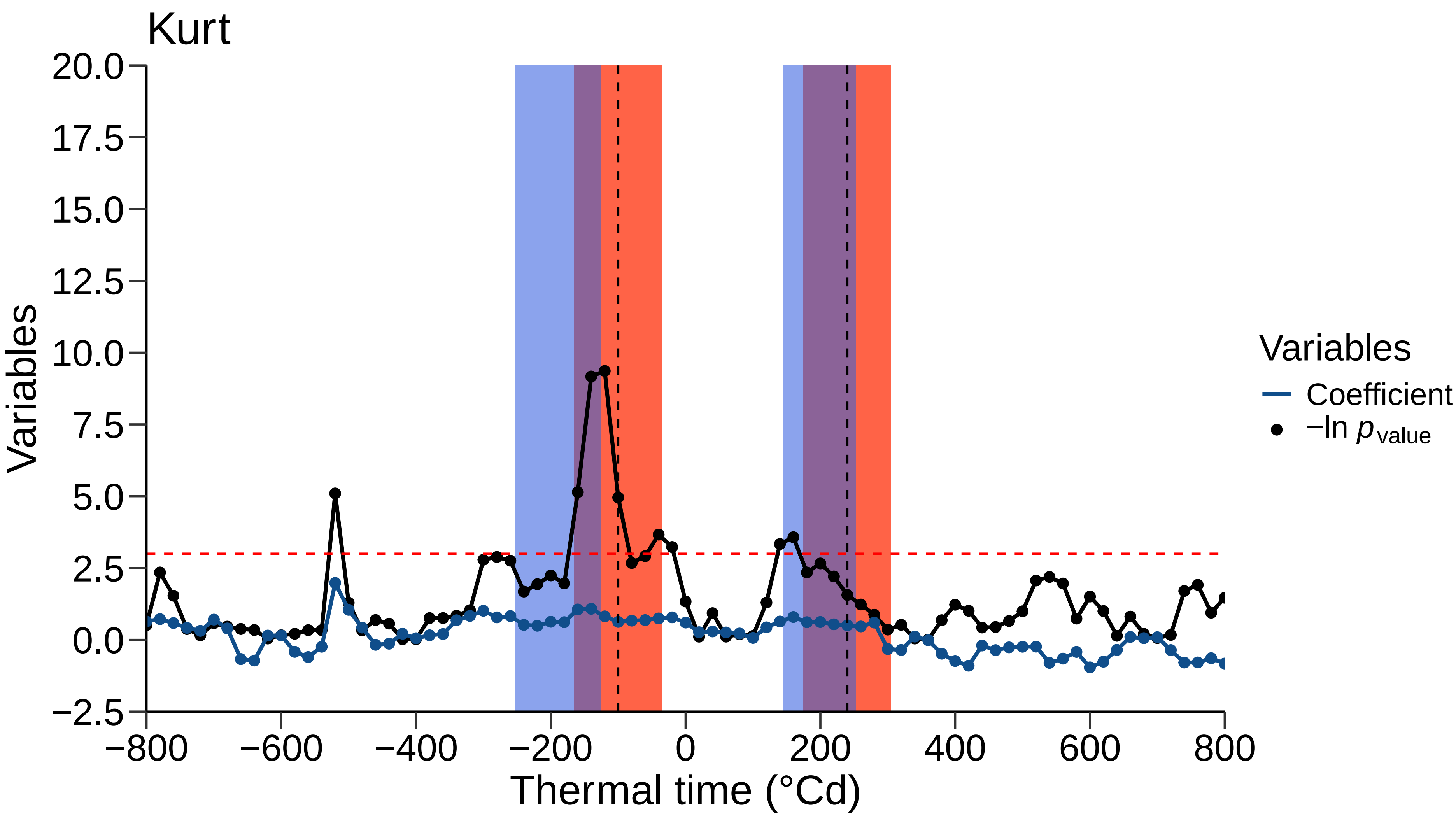
Kurt
20.0
17.5
15.0
12.5
10.0
7.5
5.0
2.5
0.0
−2.5
Variables
−800
−600
−400
−200
0
200
400
600
800
Thermal time (°Cd)
Variables
Coefficient
−ln p value

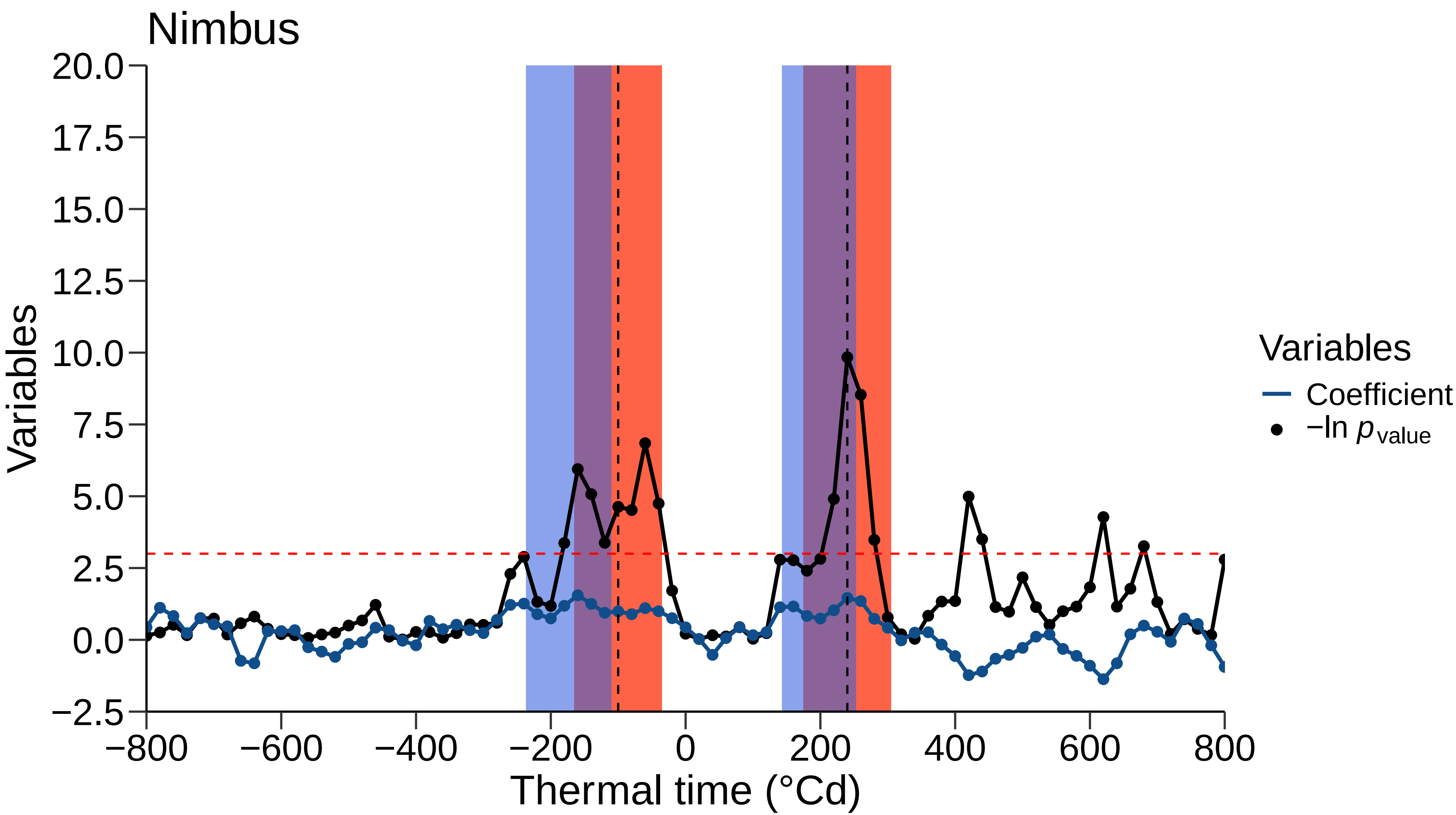

Nimbus
20.0
17.5
15.0
12.5
10.0
7.5
5.0
2.5
0.0
−2.5
Variables
−800
−600
−400
−200
0
200
400
600
800
Thermal time (°Cd)
Variables
Coefficient
−ln $p_{value}$

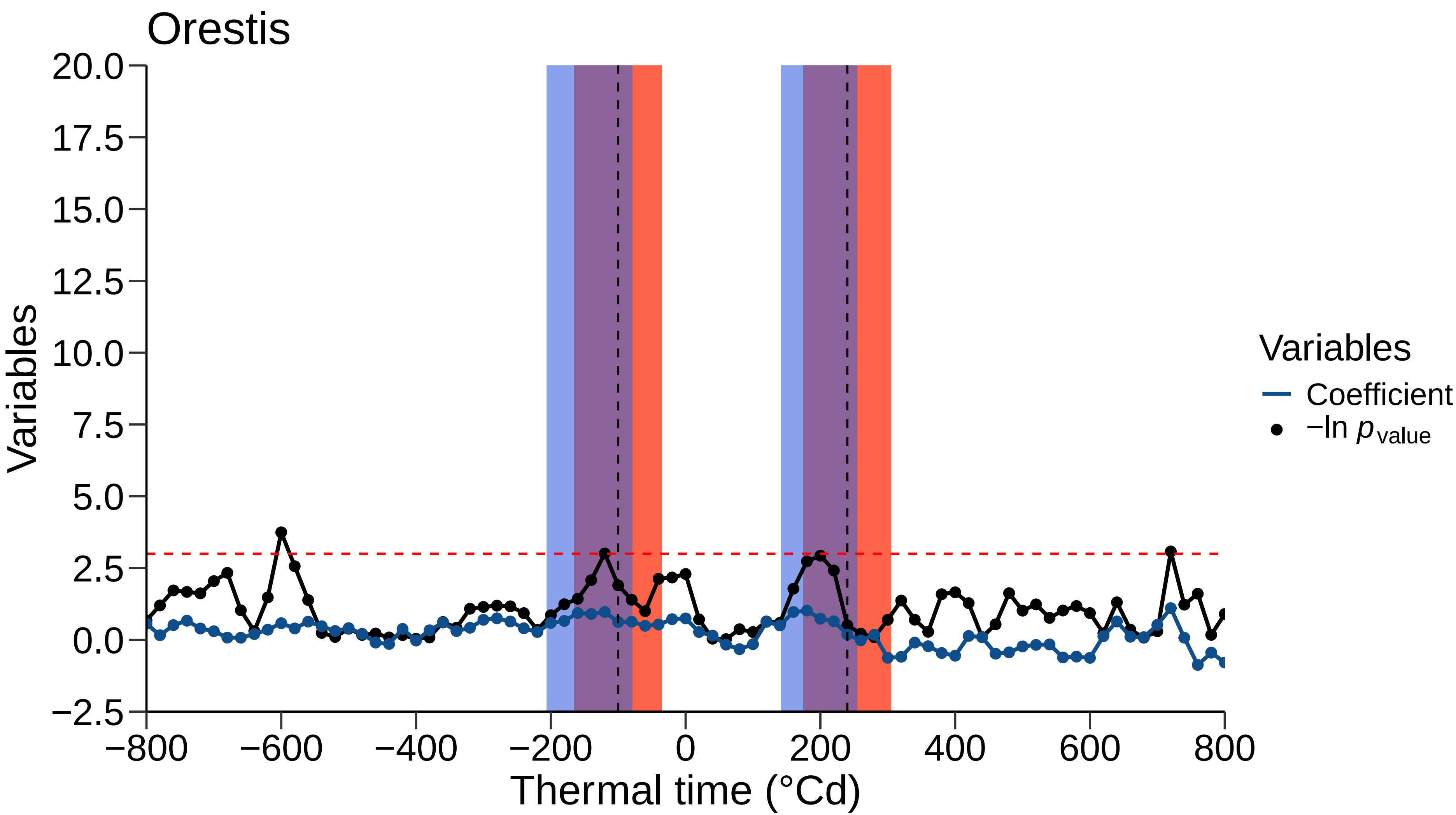

Orestis
20.0
17.5
15.0
12.5
10.0
7.5
5.0
2.5
0.0
−2.5
Variables
−800
−600
−400
−200
0
200
400
600
800
Thermal time (°Cd)
Variables
Coefficient
−ln $p_{value}$

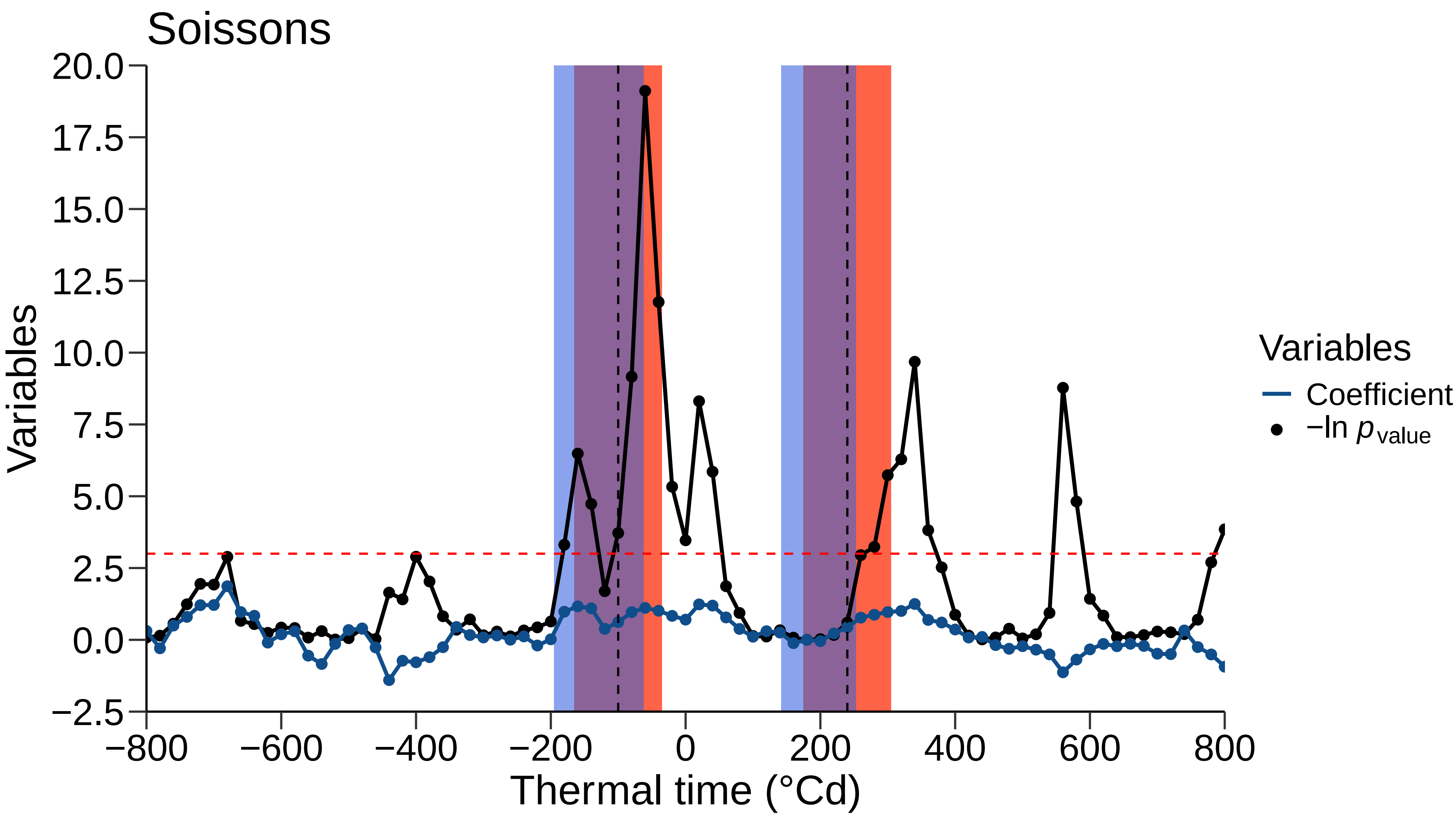
Soissons
Variables
20.0
17.5
15.0
12.5
10.0
7.5
5.0
2.5
0.0
−2.5
−800
−600
−400
−200
0
200
400
600
800
Thermal time (°Cd)
Variables
Coefficient
−ln p value

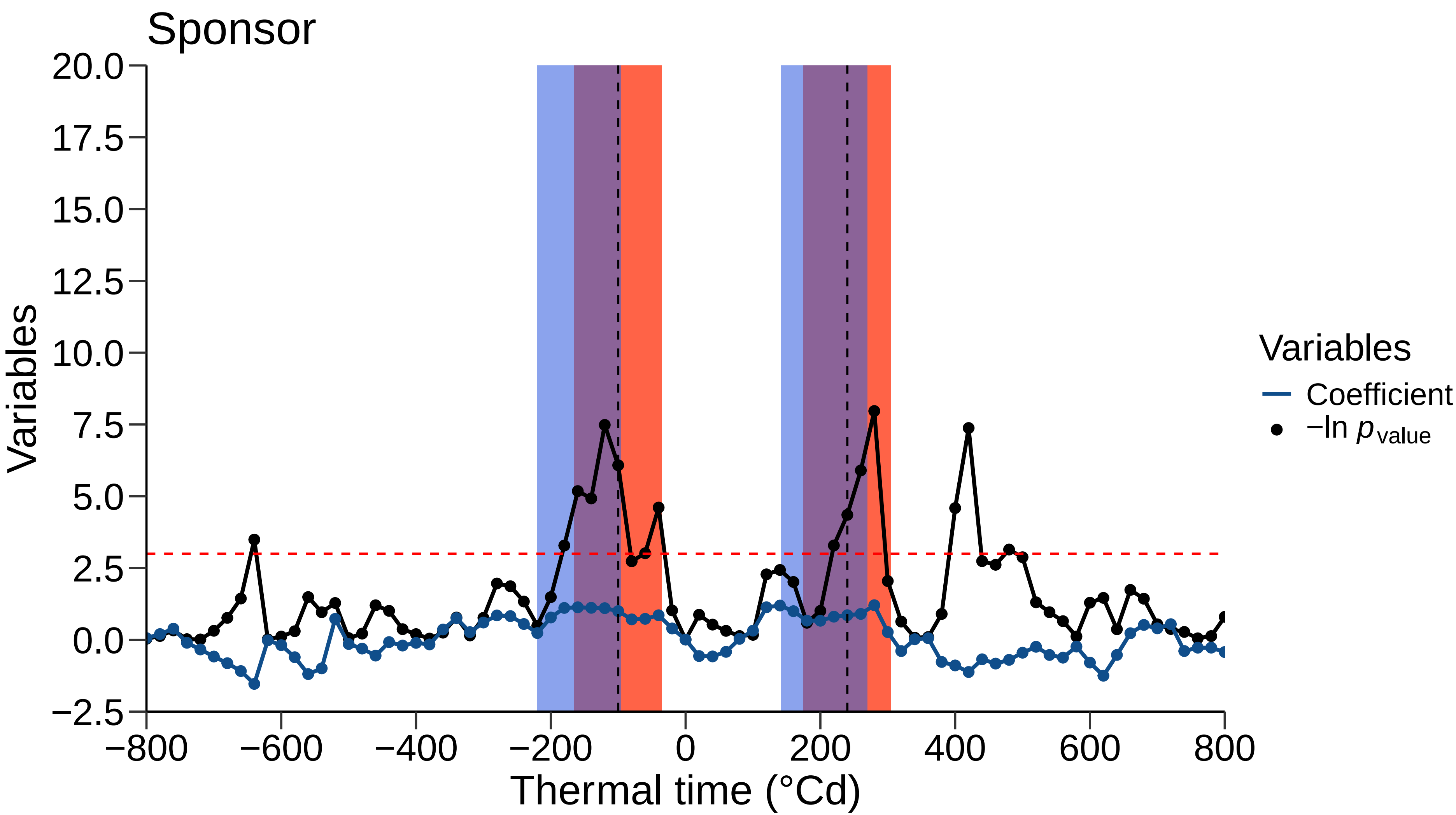

Sponsor
20.0
17.5
15.0
12.5
10.0
7.5
5.0
2.5
0.0
−2.5
Variables
−800
−600
−400
−200
0
200
400
600
800
Thermal time (°Cd)
Variables
Coefficient
−ln $p_{value}$

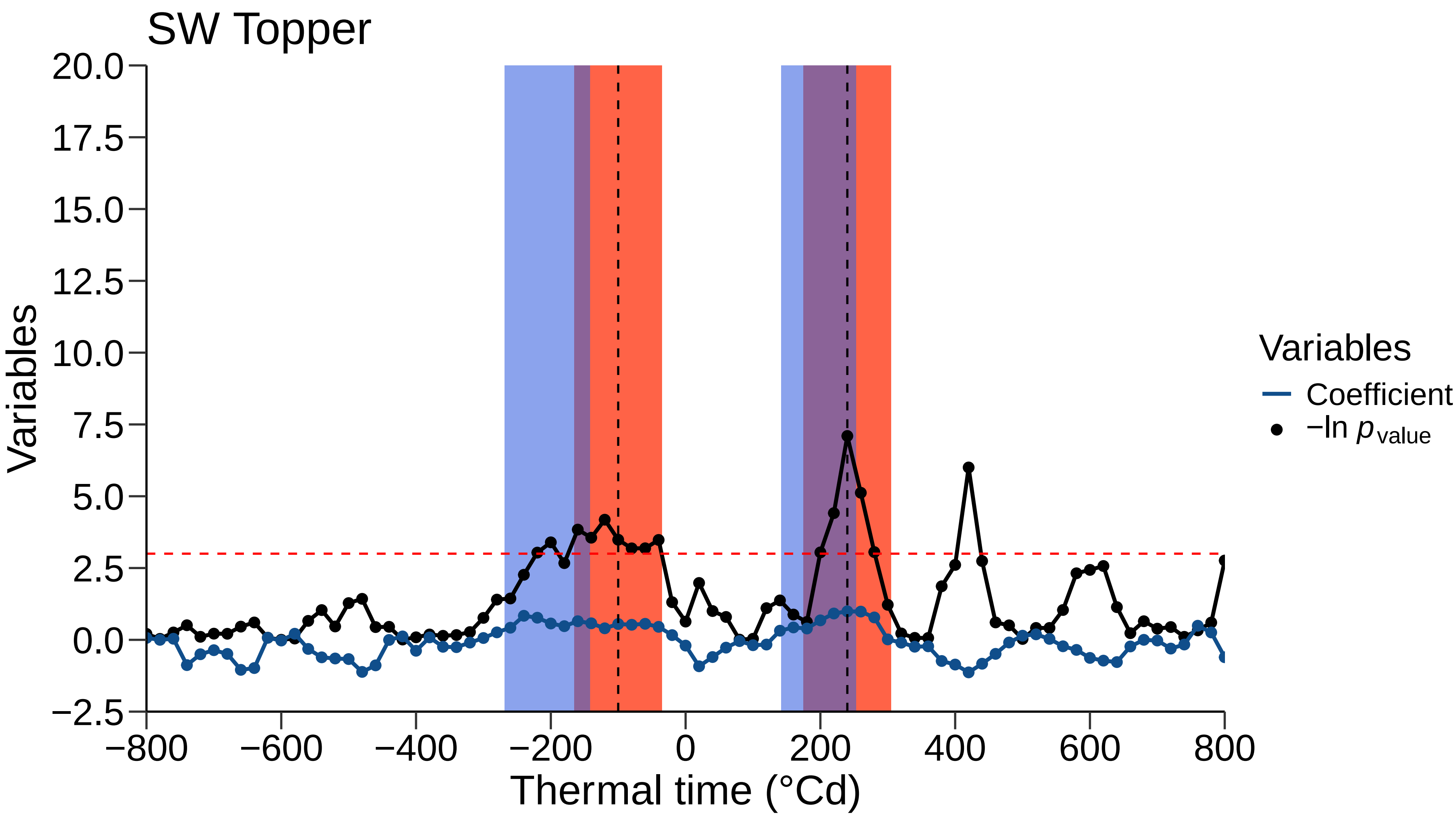
SW Topper
Variables
20.0
17.5
15.0
12.5
10.0
7.5
5.0
2.5
0.0
−2.5
−800
−600
−400
−200
0
200
400
600
800
Thermal time (°Cd)
Variables
Coefficient
−ln $p_{value}$

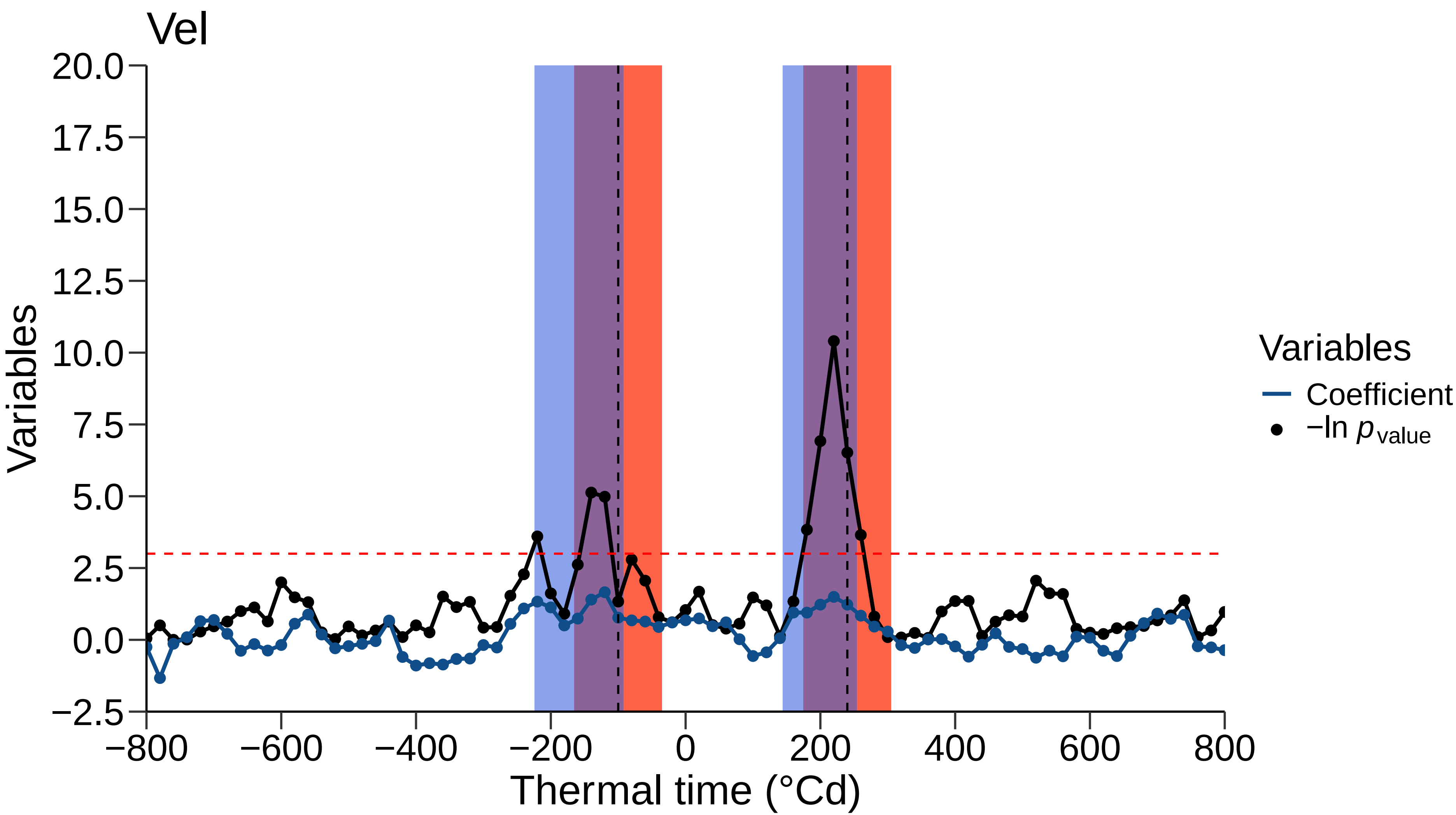

Vel
20.0
17.5
15.0
12.5
10.0
7.5
5.0
2.5
0.0
−2.5
Variables
−800
−600
−400
−200
0
200
400
600
800
Thermal time (°Cd)
Variables
Coefficient
−ln $p_{value}$

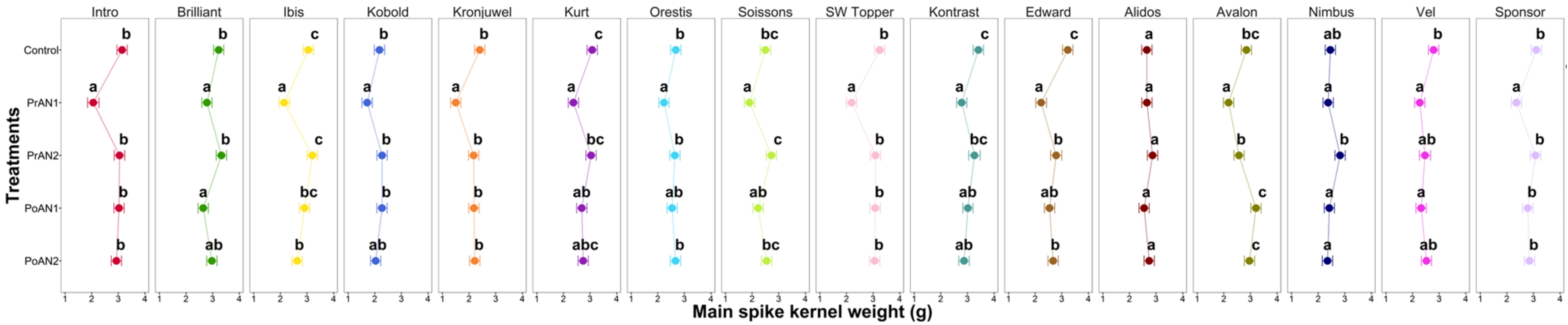


***Fig S3.*** The interaction effects of genotype and light on main spike kernel weight per plant (g). Each point represents the estimated marginal mean derived from the linear mixed effects model, with 20 individual plants nested within two replicates/chambers. Error bars indicate 95% confidence intervals. Different letters denote statistically significant differences among treatments according to Sidak adjusted pairwise comparisons ($p \leq 0.05$). Control, with high light throughout the experimental period; PrAN1, where low light was applied during the sensitive YA-TP period followed by high light during the HD-AN sub-phase, and vice versa in PrAN2; PoAN1, where low light was applied during the sensitive PGF to early GF sub-phase followed by high light during GF, and vice versa in PoAN2.

***(Starting rom next page)***

***Figure S4*.** Mapping of total florets, and kernels number (x-axis) of 14 genotypes at each spikelet position (y-axis, from spikelet 1, at the base of the spike, to *n*, the terminal spikelet of the spike) for two treatments control (left panel), PrAN1 (middle panel) and PrAN2 (right panel). Segments in each symbol stand for the standard error of the means. The mean differences between treatments at each spikelet position was identified by t-test ($n = 20$). Asterisks and ns represent that the differences in mean between treatments and control are significant ($p < 0.05$) or insignificant, respectively.

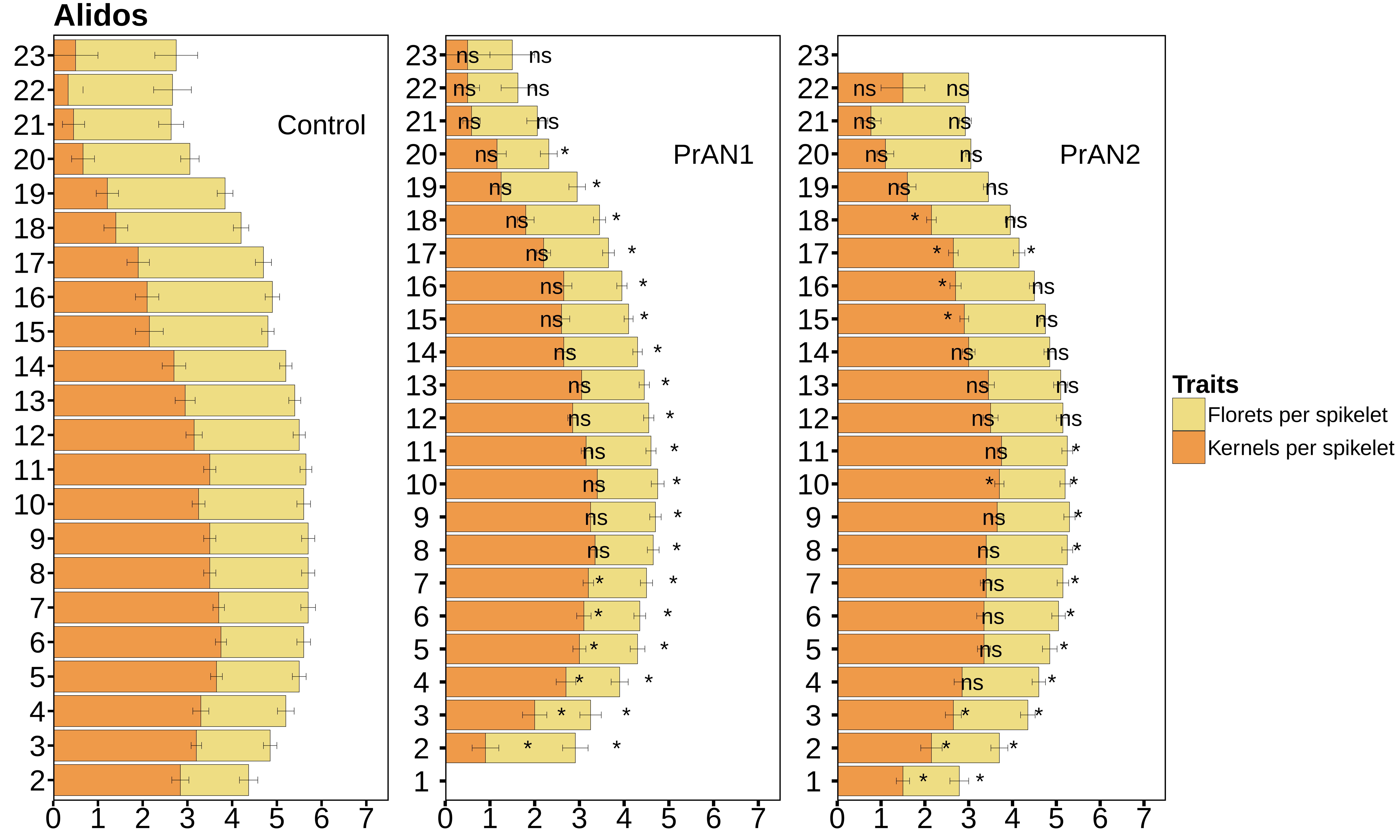
Alidos
Control
PrAN1
PrAN2
Traits
Florets per spikelet
Kernels per spikelet

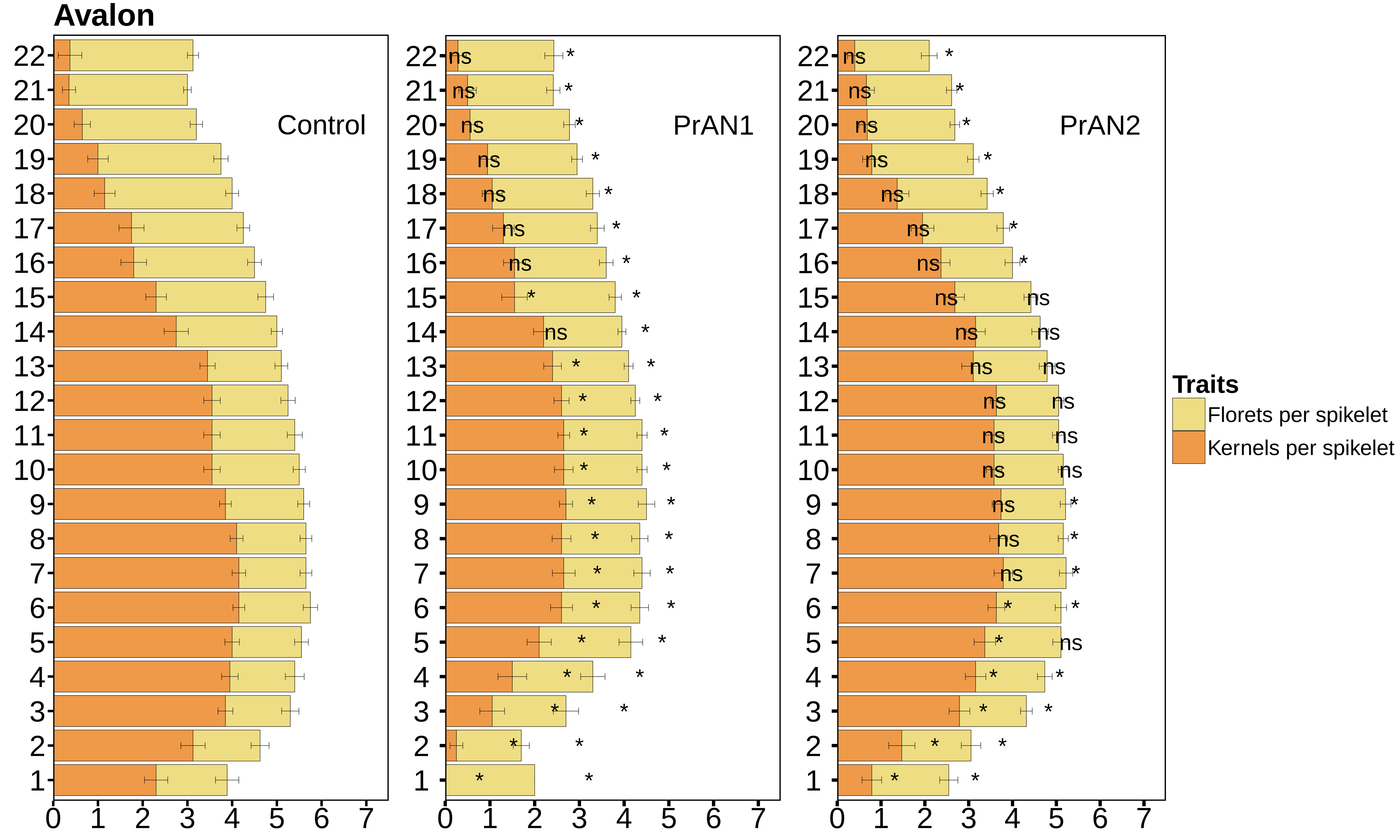

Avalon
Control
PrAN1
PrAN2
Traits
Florets per spikelet
Kernels per spikelet

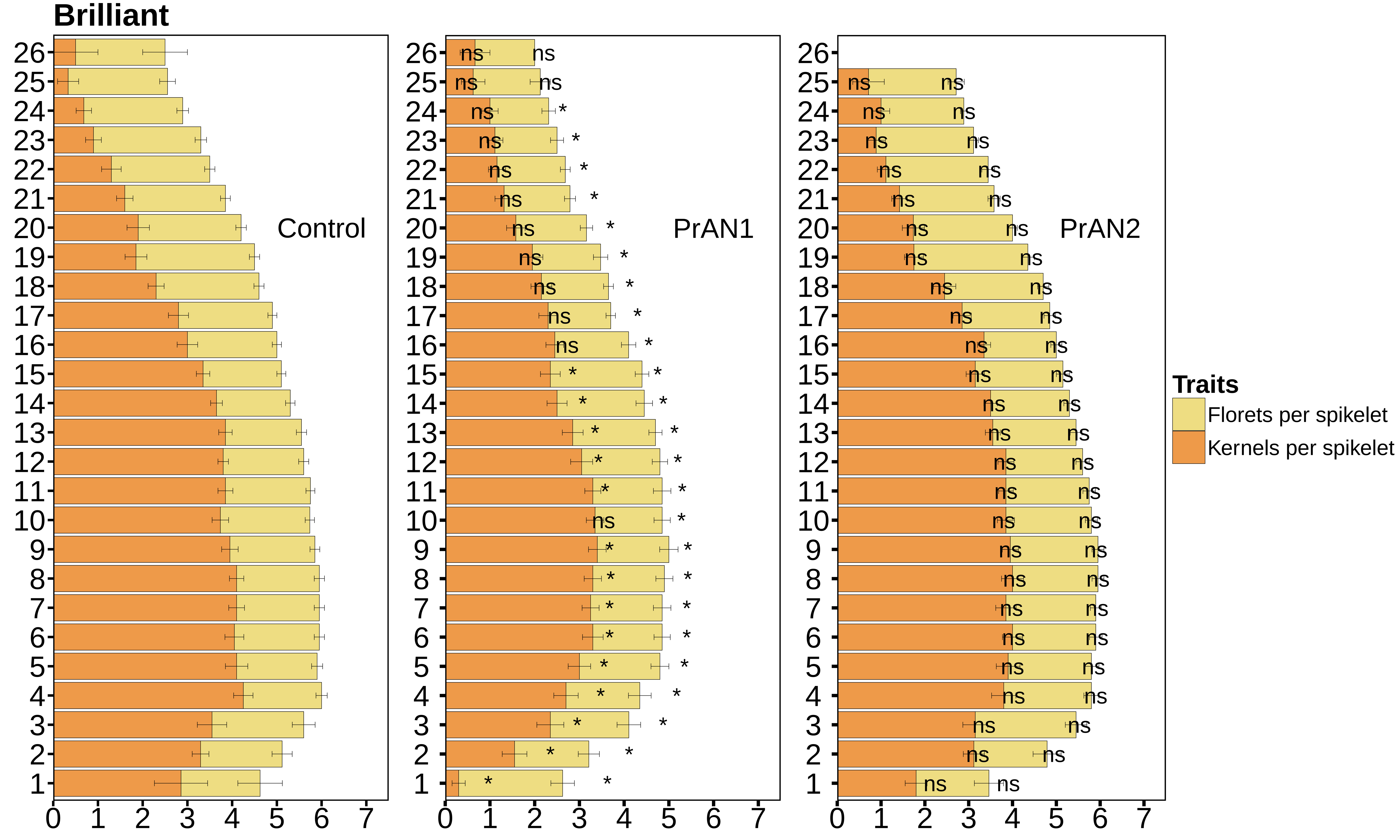
Brilliant
Control
PrAN1
PrAN2
Traits
Florets per spikelet
Kernels per spikelet
0 1 2 3 4 5 6 7
1 2 3 4 5 6 7 8 9 10 11 12 13 14 15 16 17 18 19 20 21 22 23 24 25 26

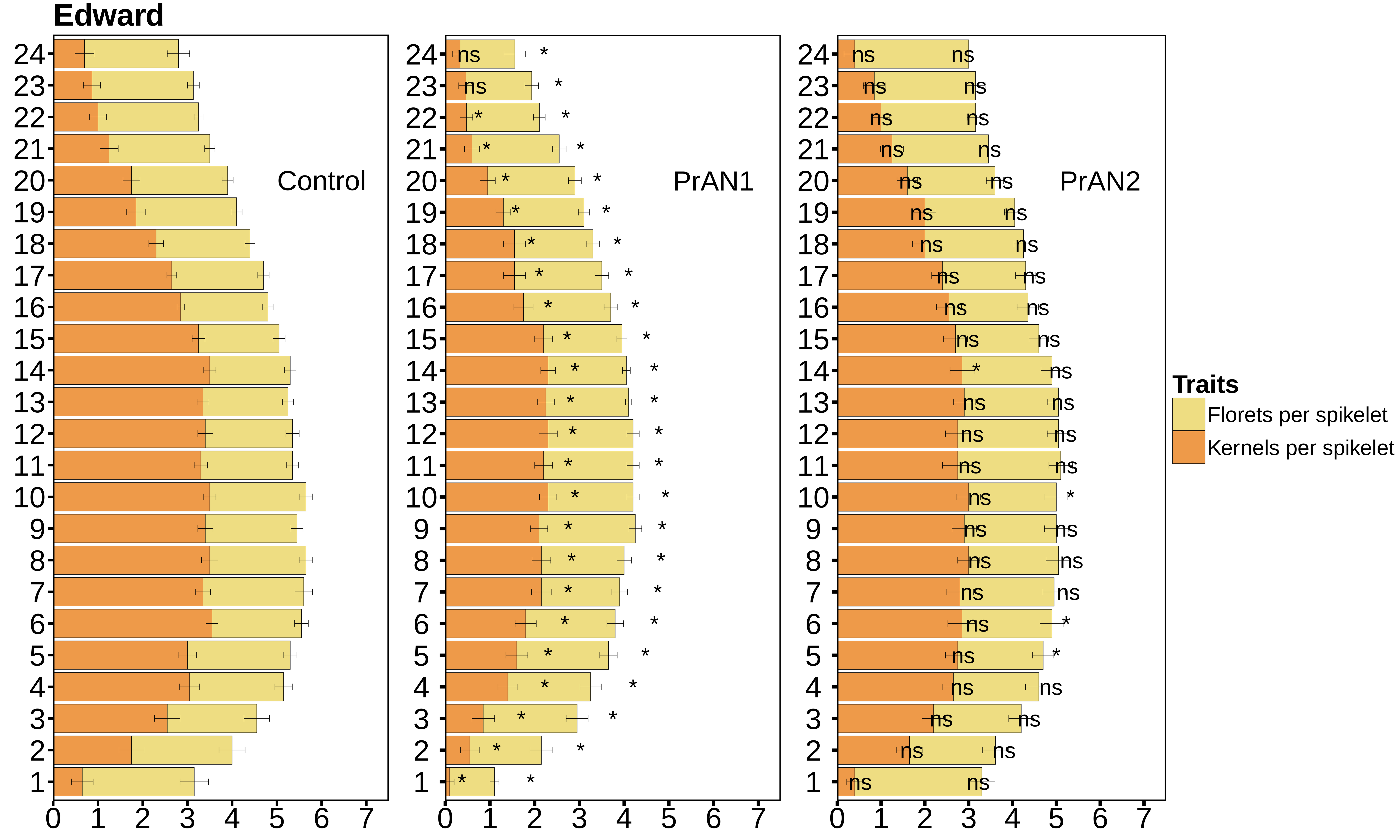

Edward
Control
PrAN1
PrAN2
Traits
Florets per spikelet
Kernels per spikelet

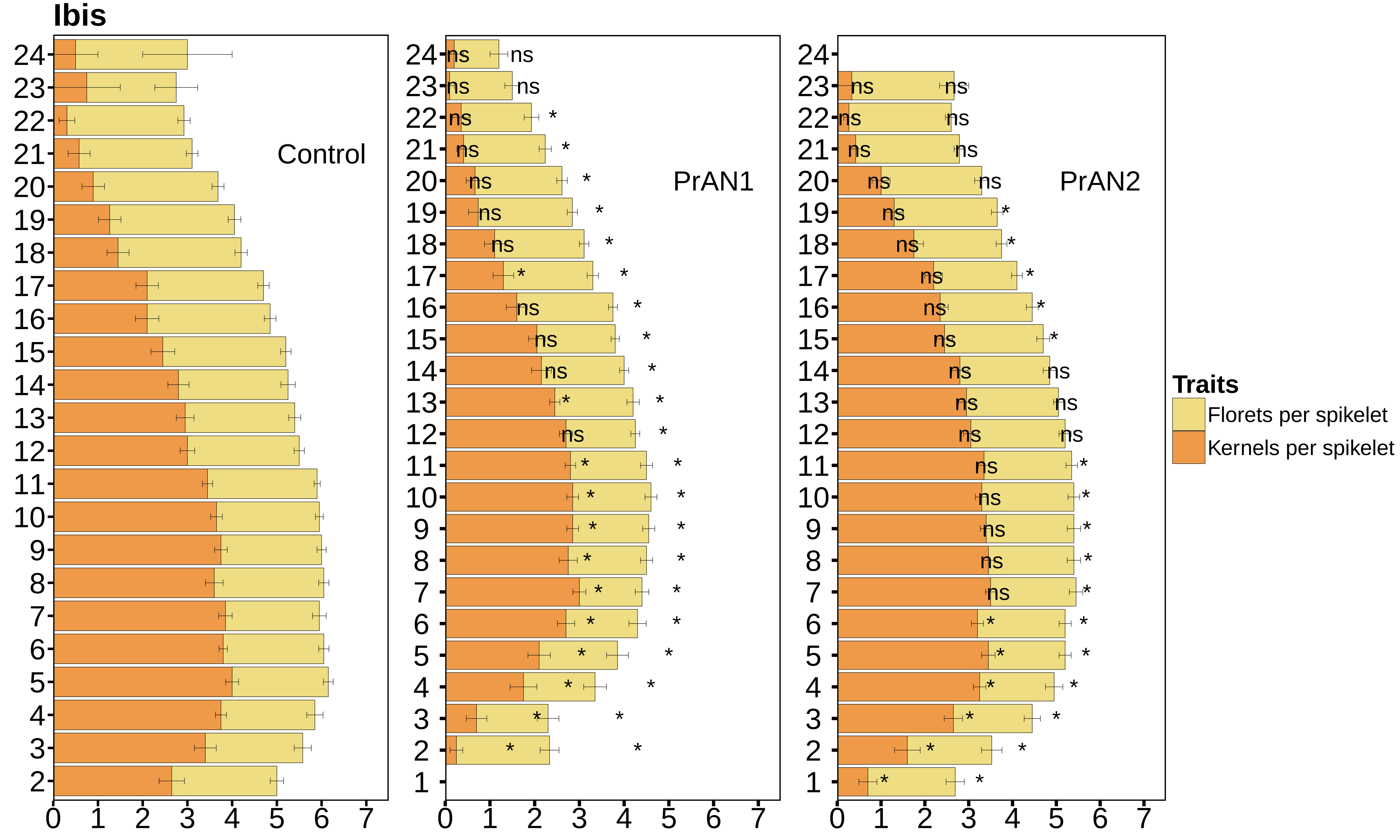

Ibis
Control
PrAN1
PrAN2
Traits
Florets per spikelet
Kernels per spikelet

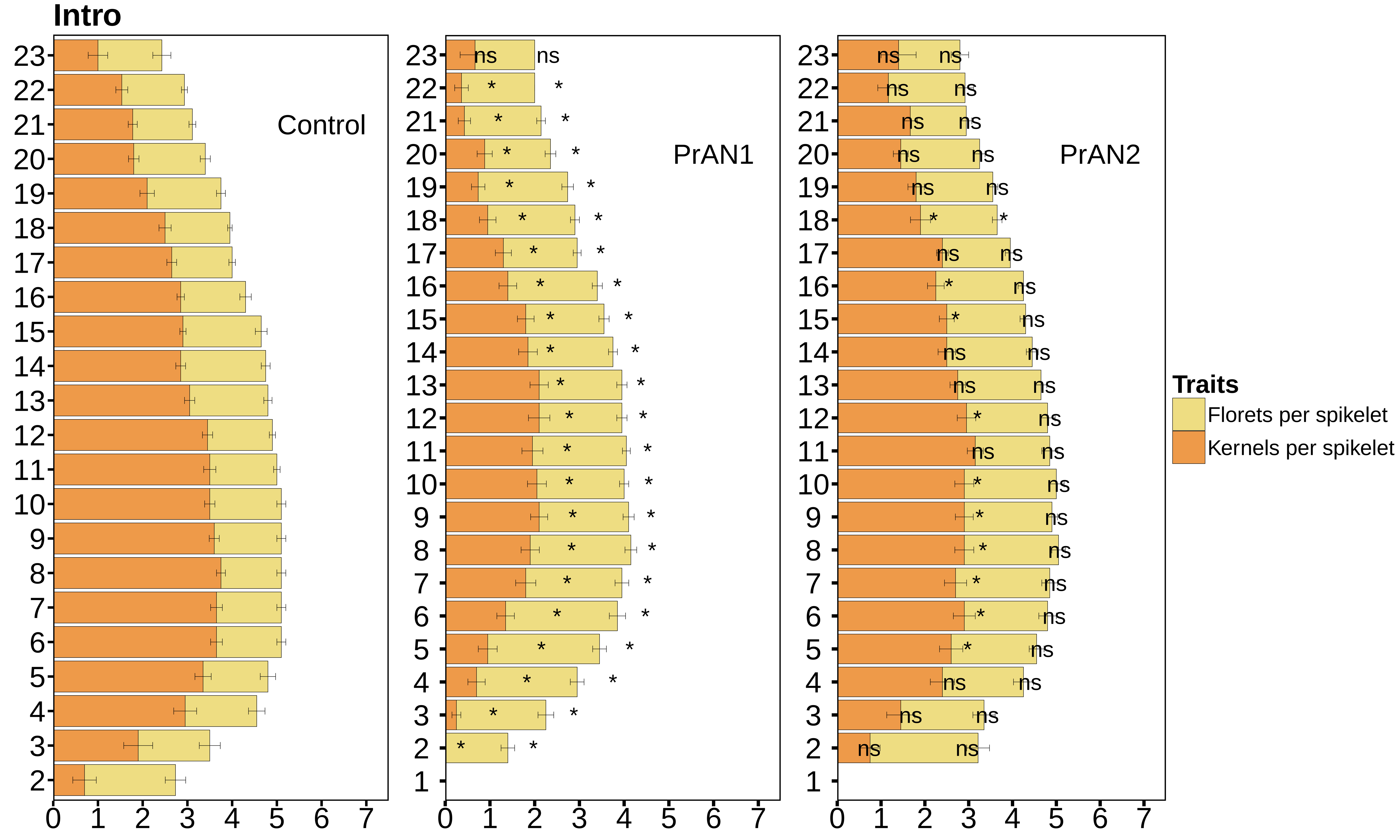

Intro
Control
PrAN1
PrAN2
Traits
Florets per spikelet
Kernels per spikelet

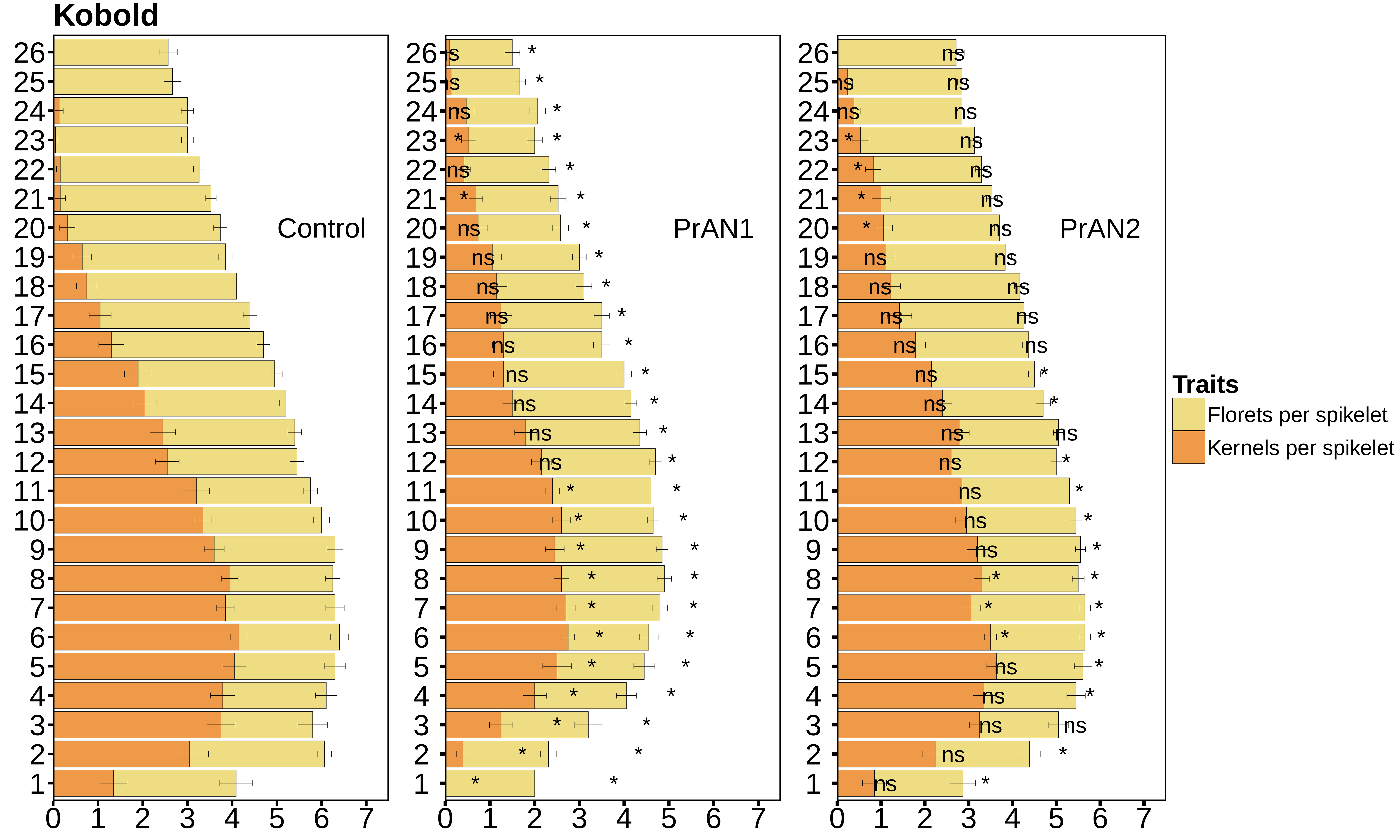

Kobold
Control
PrAN1
PrAN2
Traits
Florets per spikelet
Kernels per spikelet

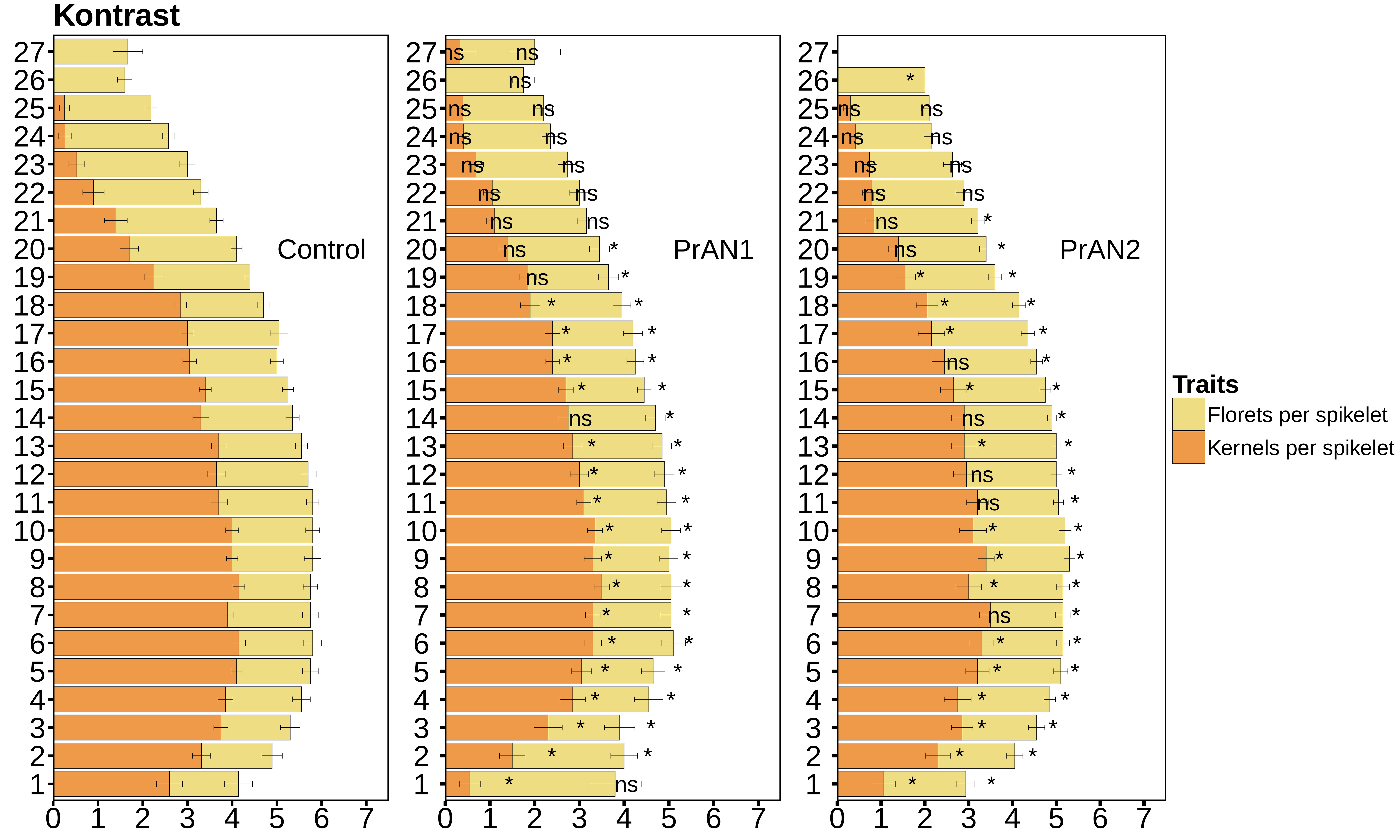
Kontrast
Control
PrAN1
PrAN2
Traits
Florets per spikelet
Kernels per spikelet

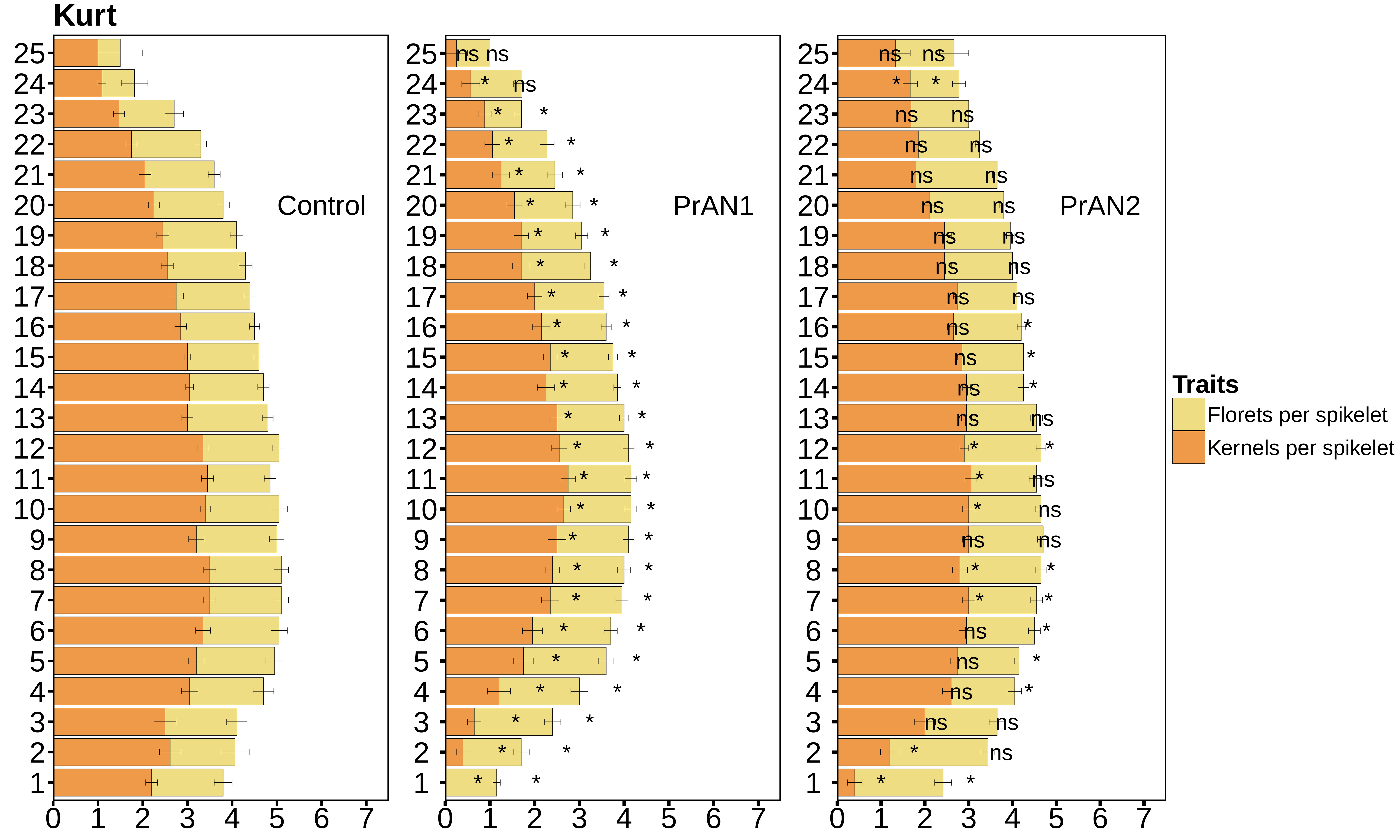
Kurt
Control
PrAN1
PrAN2
Traits
Florets per spikelet
Kernels per spikelet
0 1 2 3 4 5 6 7
1 2 3 4 5 6 7 8 9 10 11 12 13 14 15 16 17 18 19 20 21 22 23 24 25

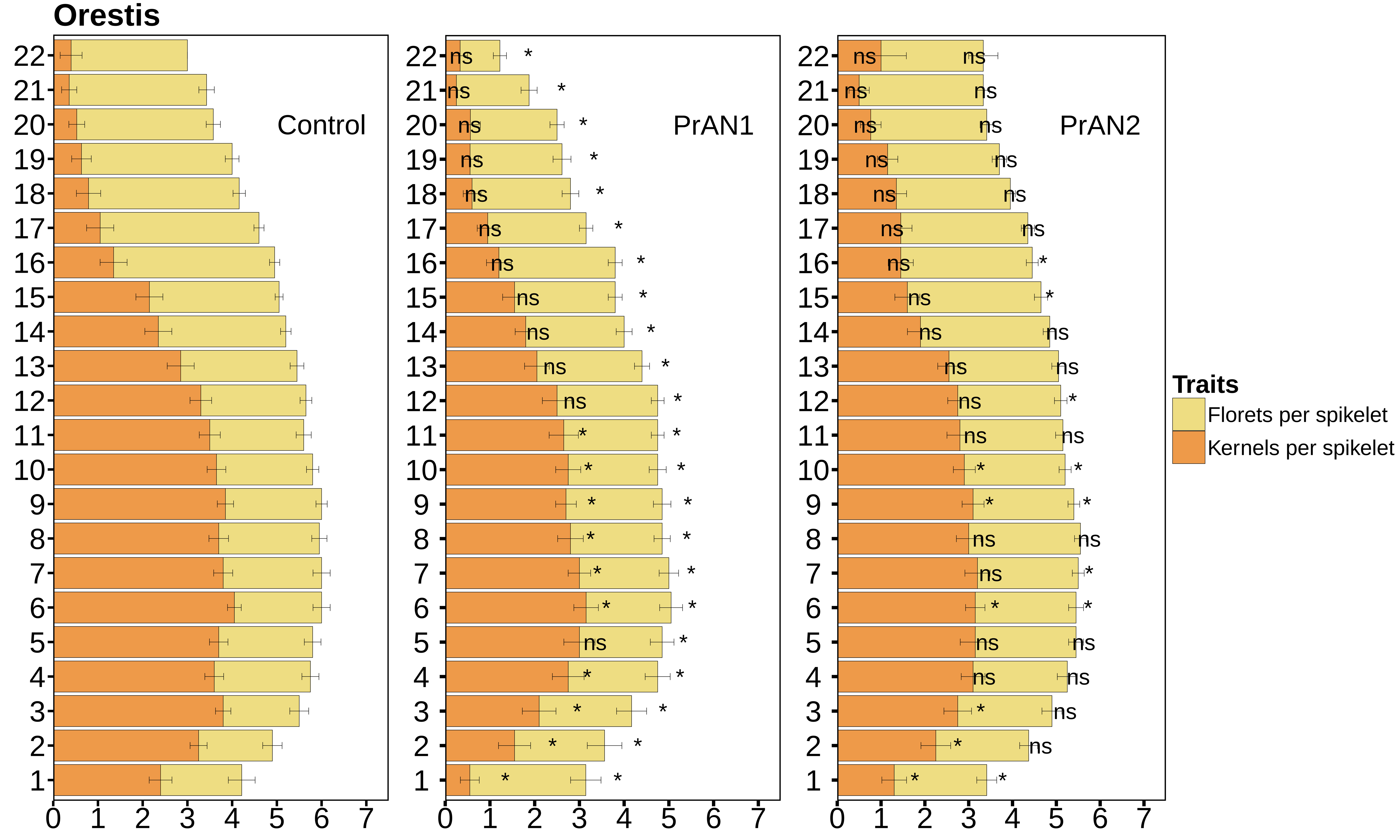

Orestis
Control
PrAN1
PrAN2
Traits
Florets per spikelet
Kernels per spikelet

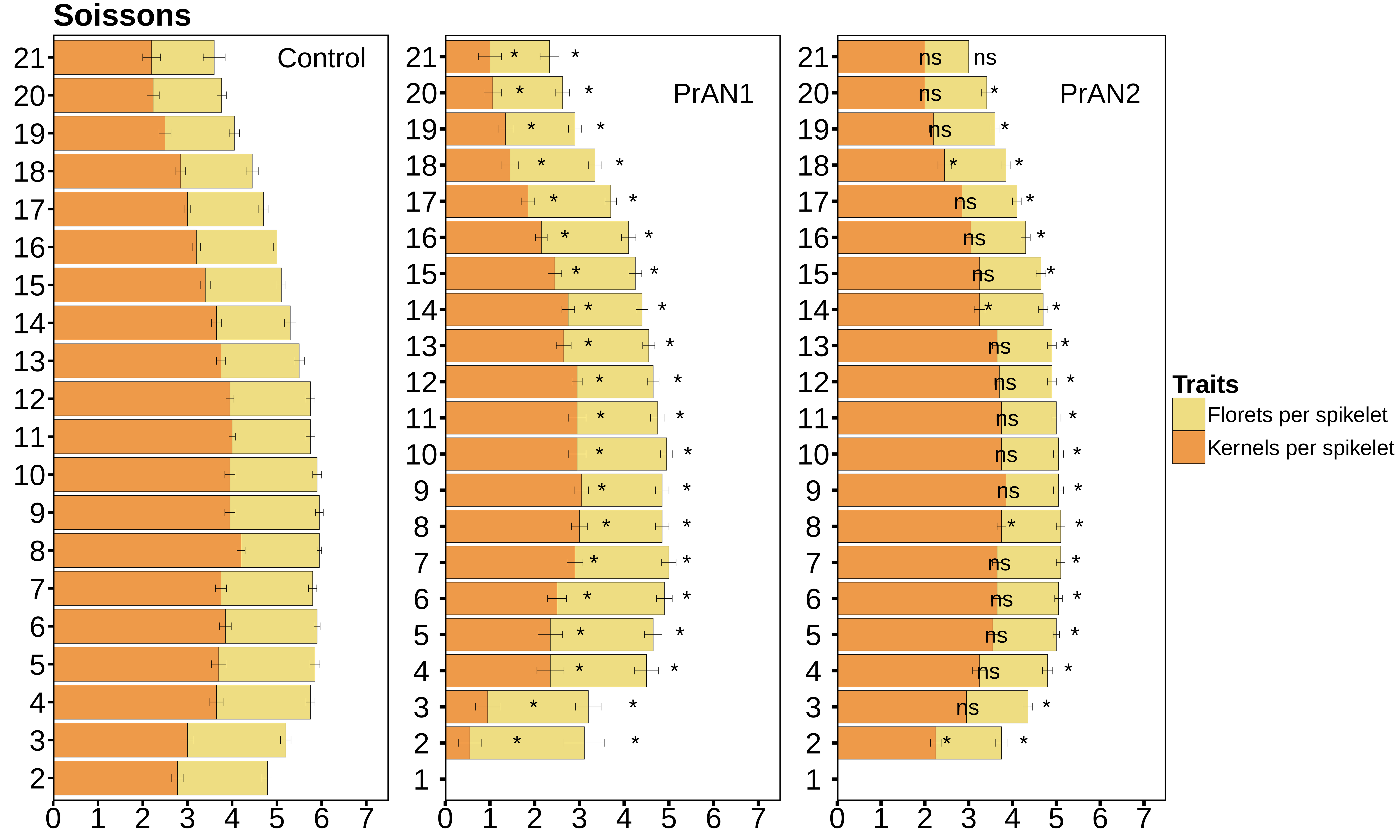

Soissons
Control
PrAN1
PrAN2
Traits
Florets per spikelet
Kernels per spikelet

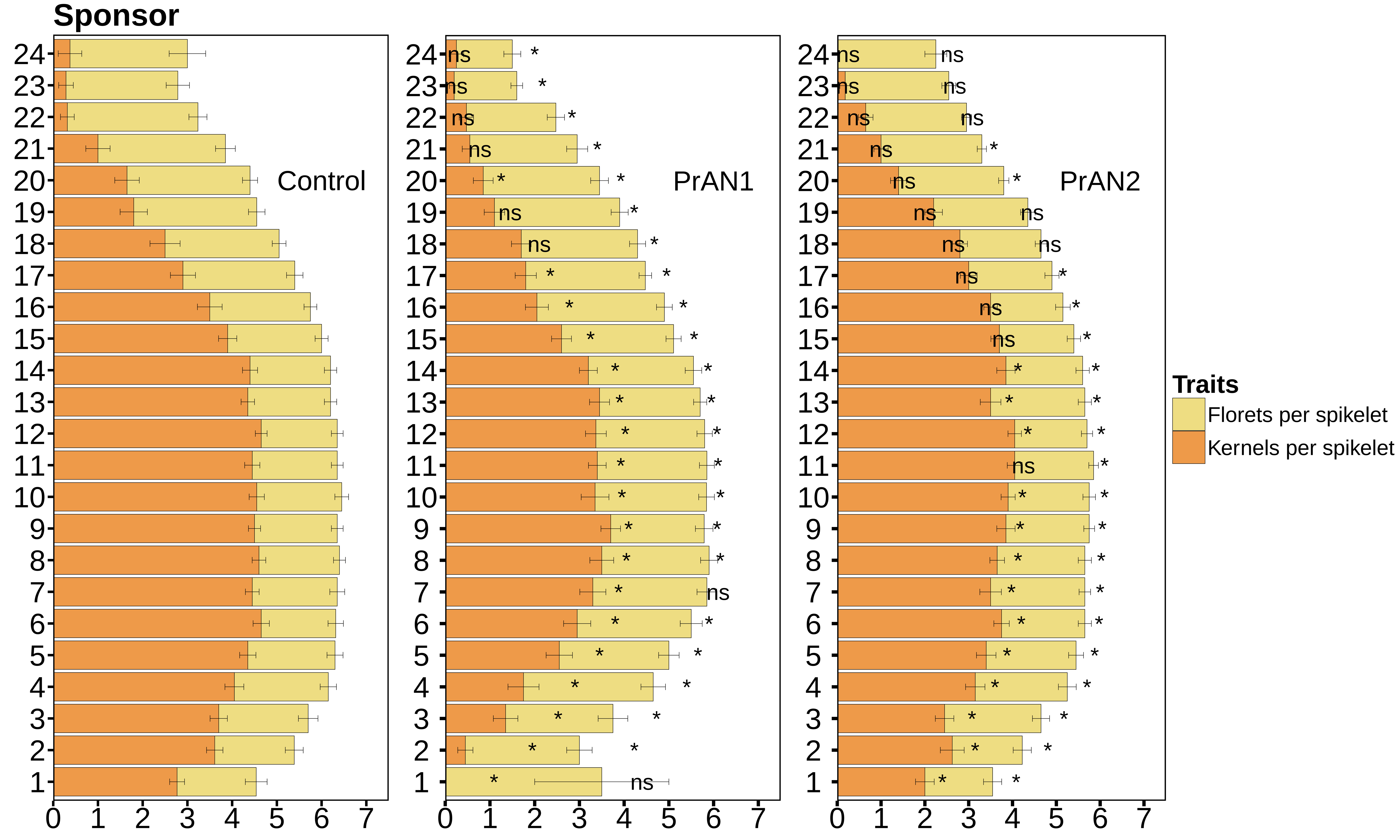

Sponsor
Control
PrAN1
PrAN2
Traits
Florets per spikelet
Kernels per spikelet

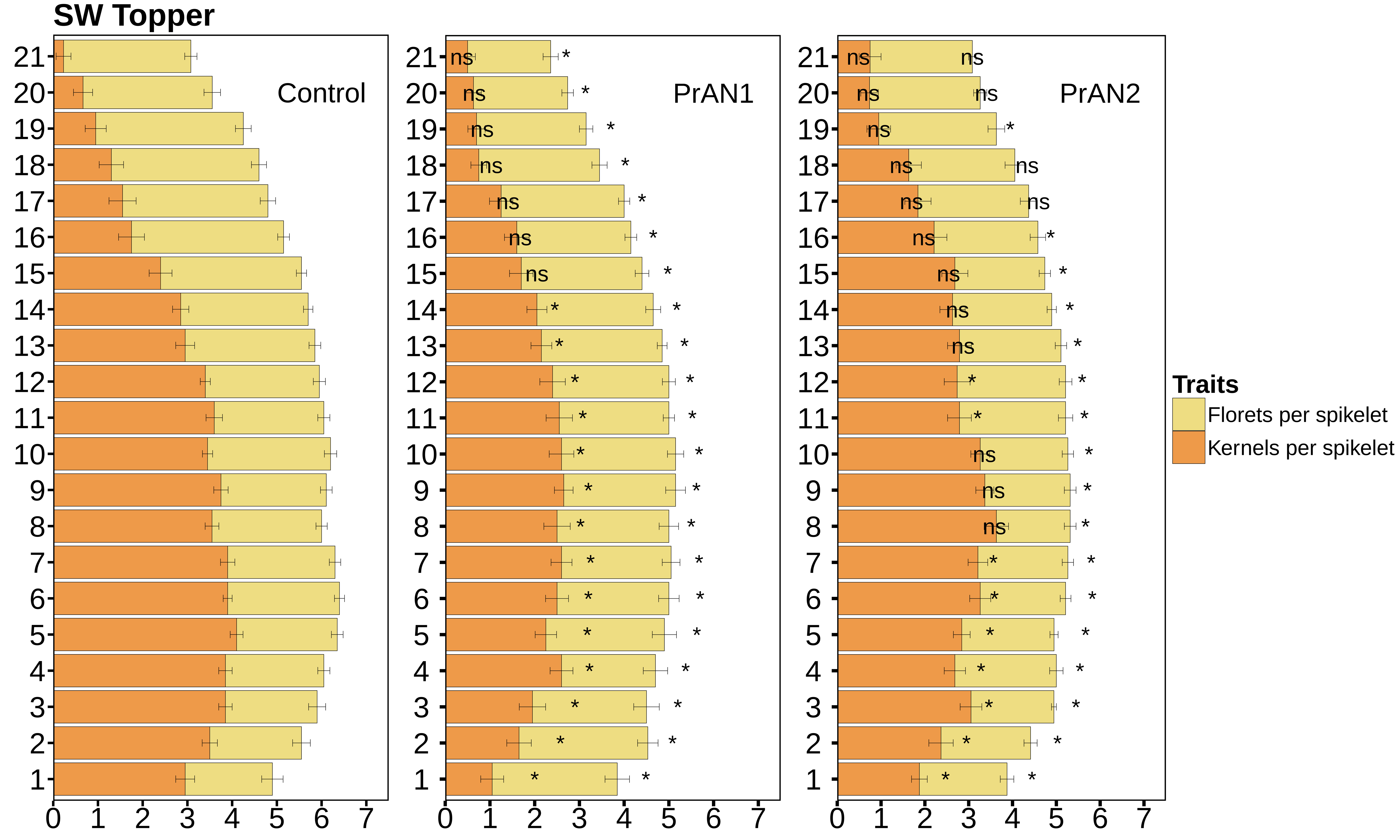

SW Topper
Control
PrAN1
PrAN2
Traits
Florets per spikelet
Kernels per spikelet

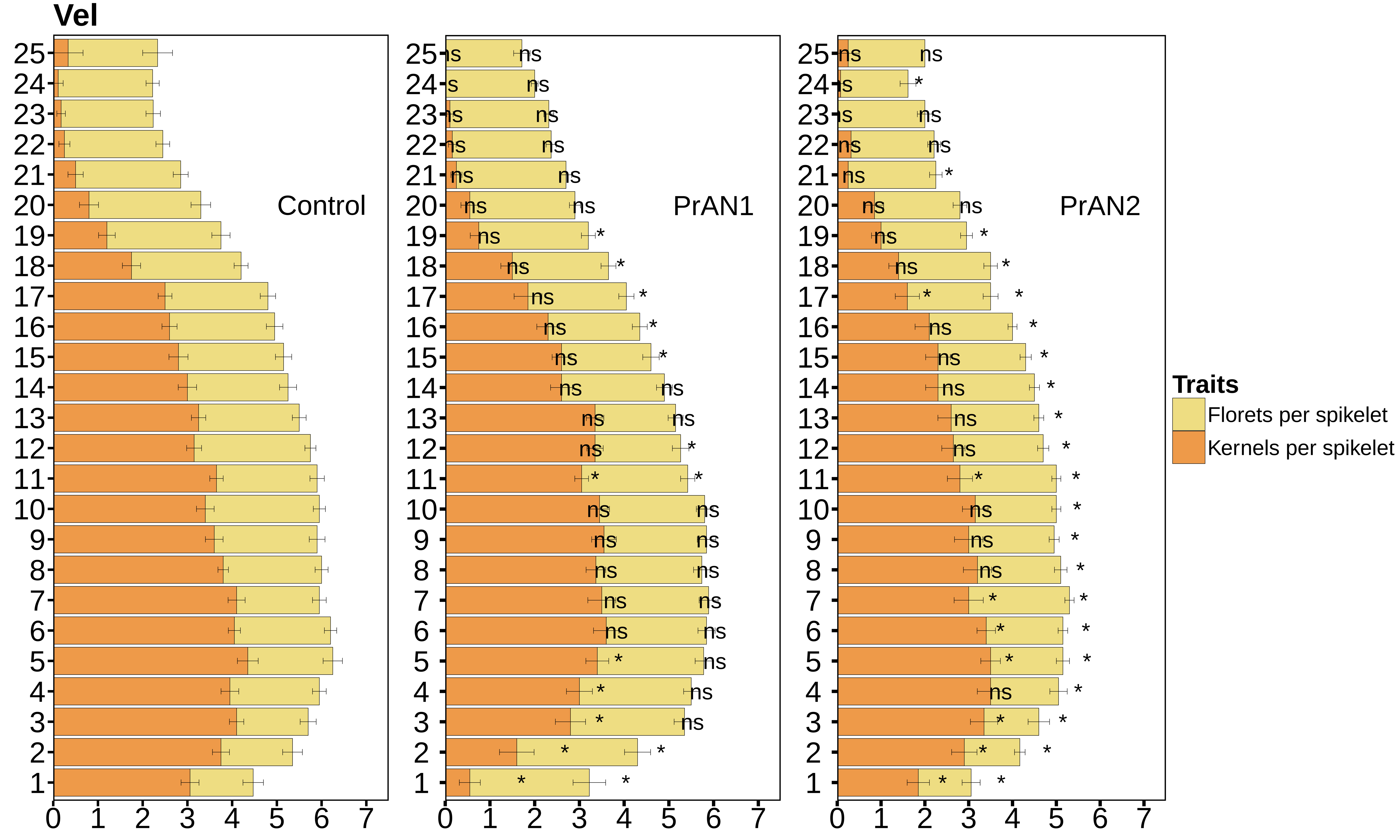

Vel
Control
PrAN1
PrAN2
Traits
Florets per spikelet
Kernels per spikelet

***(Starting from next page)***

***Figure S5*****.** Mapping of total florets, and kernels number (x-axis) of 16 genotypes at each spikelet position (y-axis, from spikelet 1, at the base of the spike, to *n*, the terminal spikelet of the spike) for two treatments control (left panel), PoAN1 (middle panel) and PoAN2 (right panel). Segments in each symbol stand for the standard error of the means. The mean differences between treatments at each spikelet position was identified by t-test ($n$ = 20). Asterisks and ns represent that the differences in mean between treatments and control are significant ($p < 0.05$) or insignificant, respectively.

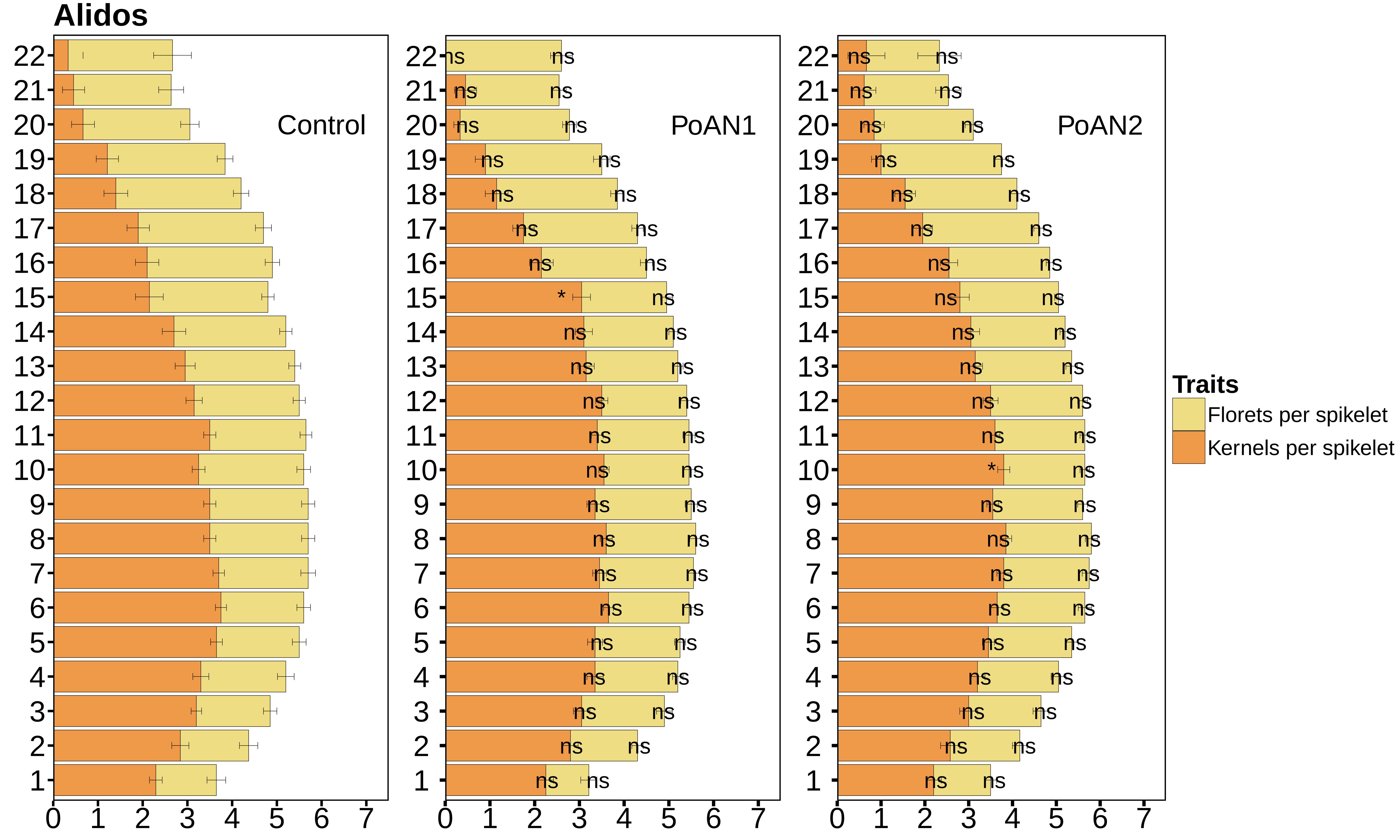

Alidos
Control
PoAN1
PoAN2
Traits
Florets per spikelet
Kernels per spikelet

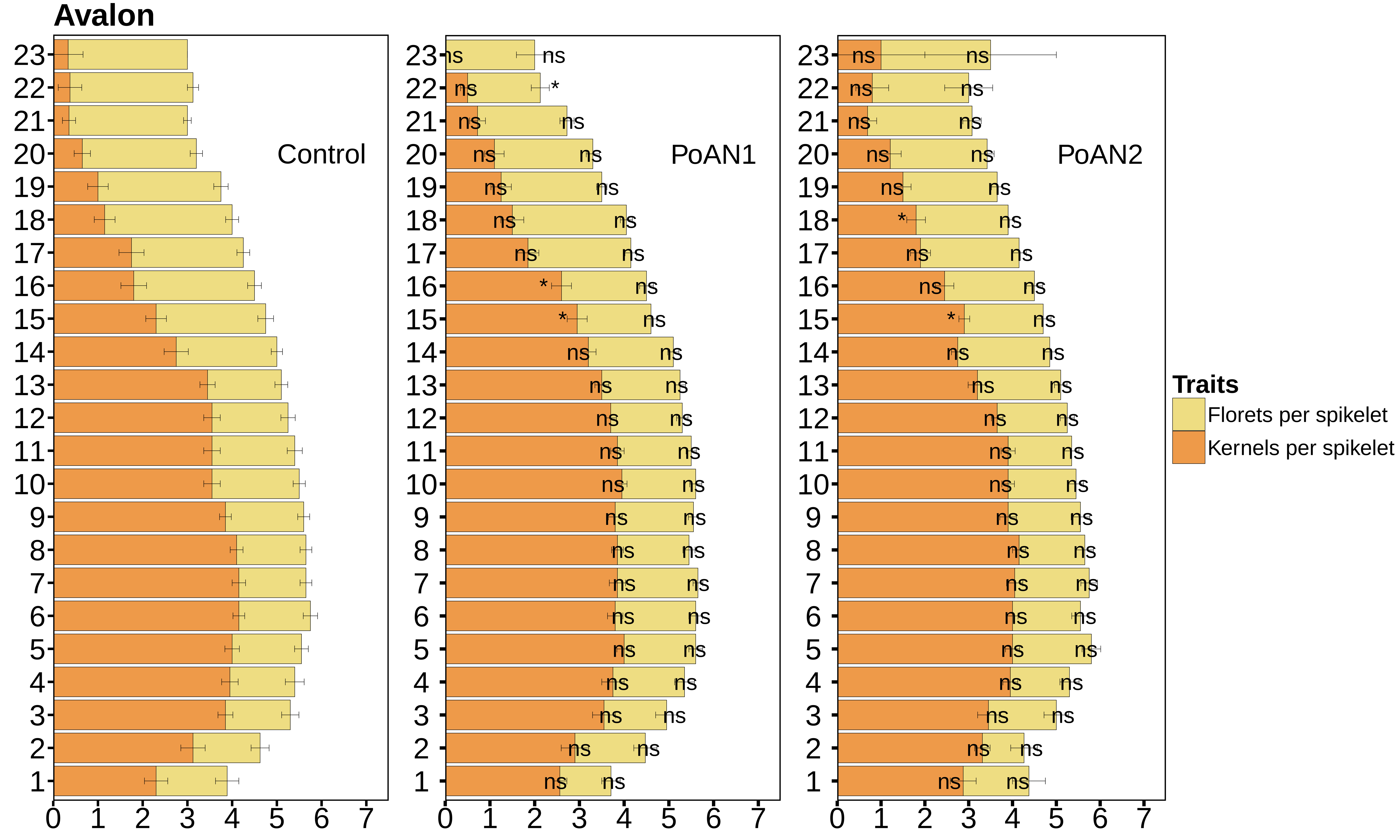
Avalon
Control
PoAN1
PoAN2
Traits
Florets per spikelet
Kernels per spikelet
23
22
21
20
19
18
17
16
15
14
13
12
11
10
9
8
7
6
5
4
3
2
1
0 1 2 3 4 5 6 7
ns
*

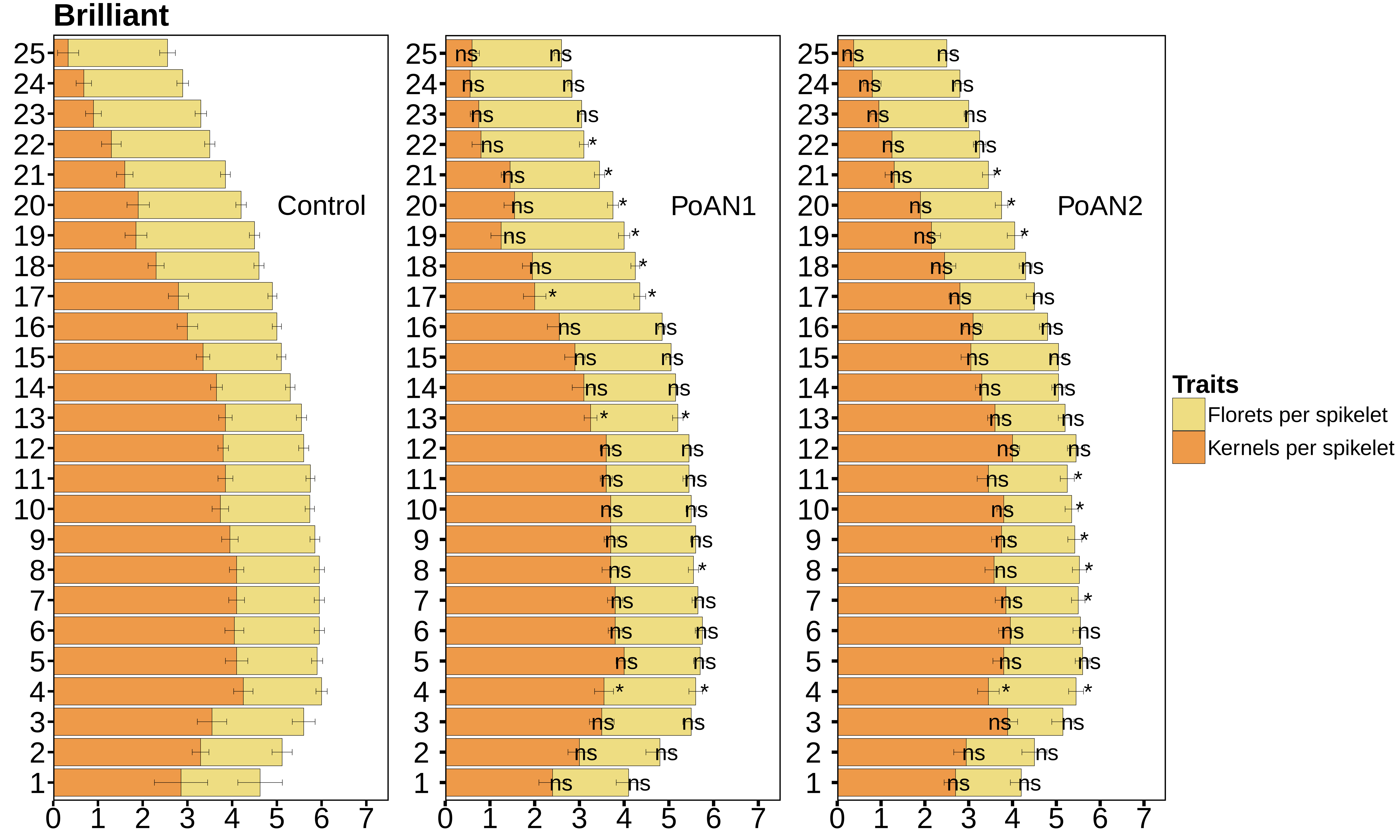

Brilliant
Control
PoAN1
PoAN2
Traits
Florets per spikelet
Kernels per spikelet

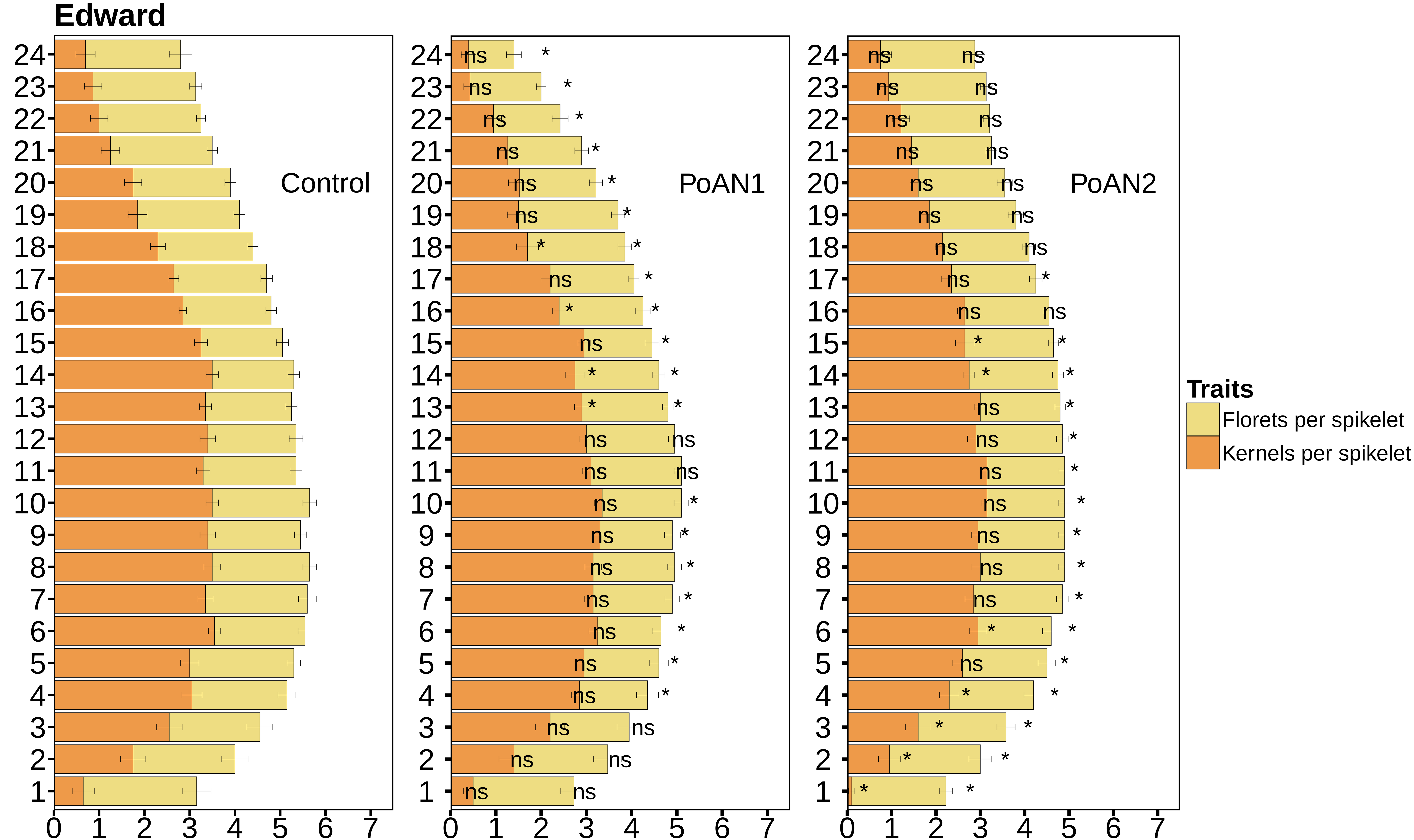
Edward
Control
PoAN1
PoAN2
Traits
Florets per spikelet
Kernels per spikelet

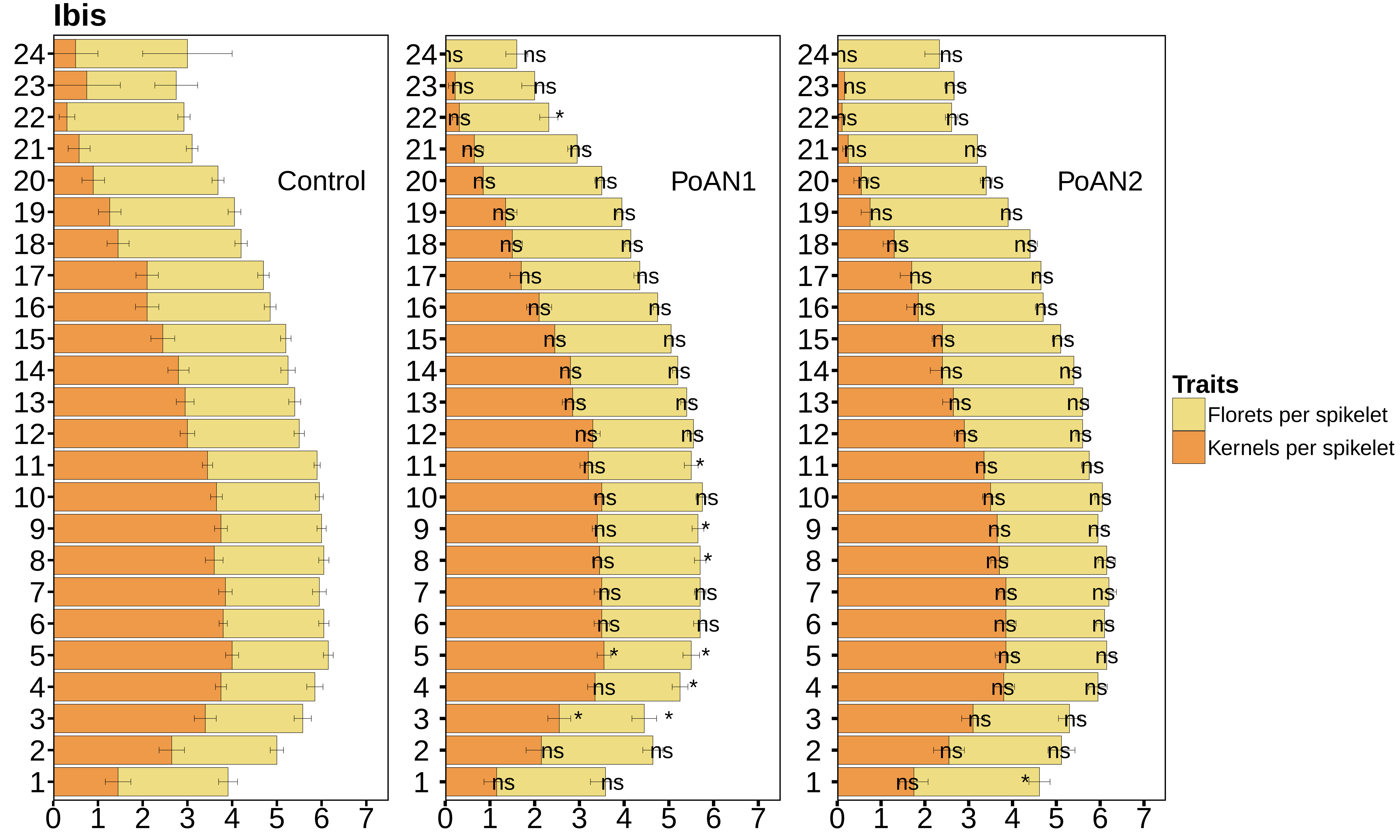

Ibis
Control
PoAN1
PoAN2
Traits
Florets per spikelet
Kernels per spikelet

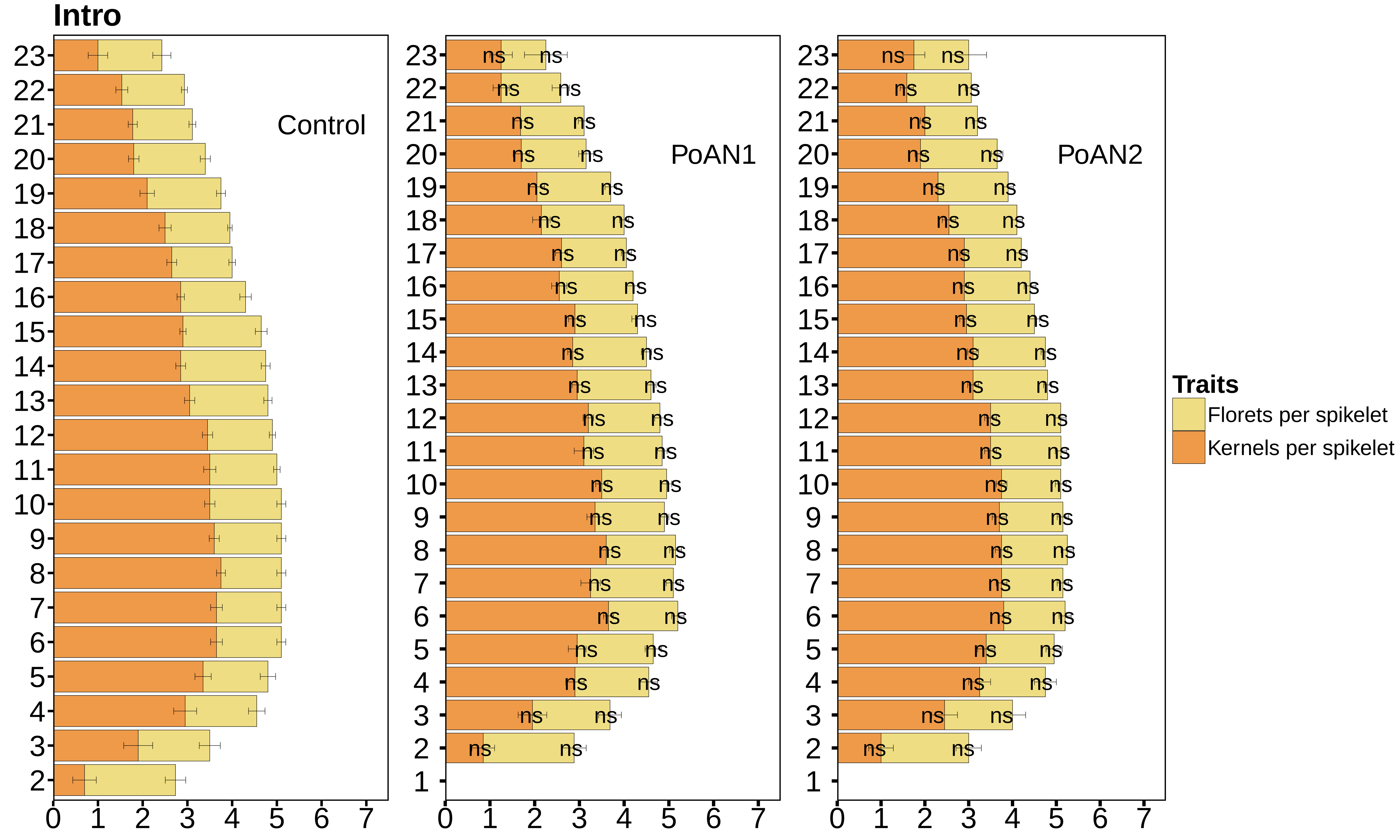

Intro
Control
PoAN1
PoAN2
ns
Traits
Florets per spikelet
Kernels per spikelet
23
22
21
20
19
18
17
16
15
14
13
12
11
10
9
8
7
6
5
4
3
2
1
0
1
2
3
4
5
6
7

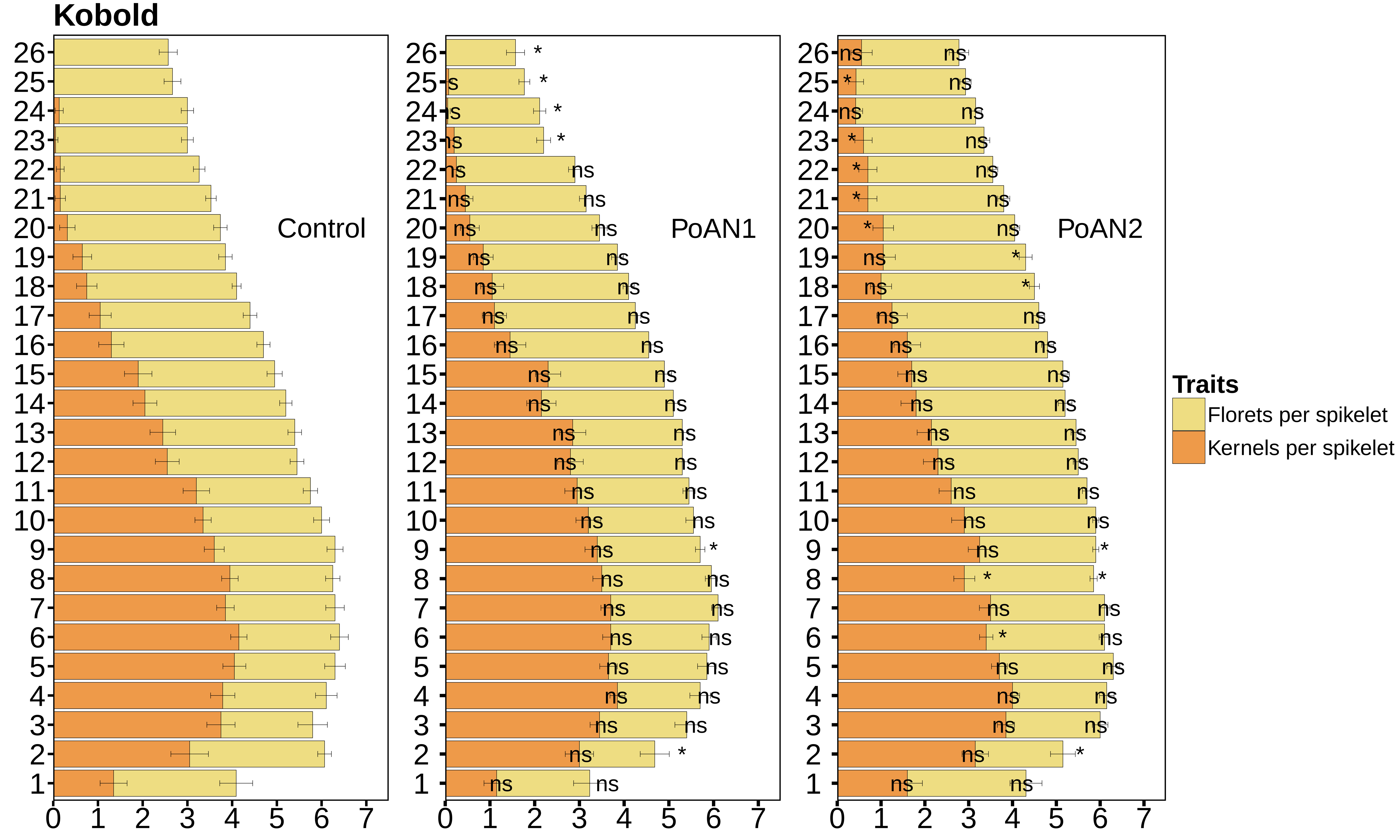
Kobold
Control
PoAN1
PoAN2
Traits
Florets per spikelet
Kernels per spikelet
26 25 24 23 22 21 20 19 18 17 16 15 14 13 12 11 10 9 8 7 6 5 4 3 2 1
0 1 2 3 4 5 6 7

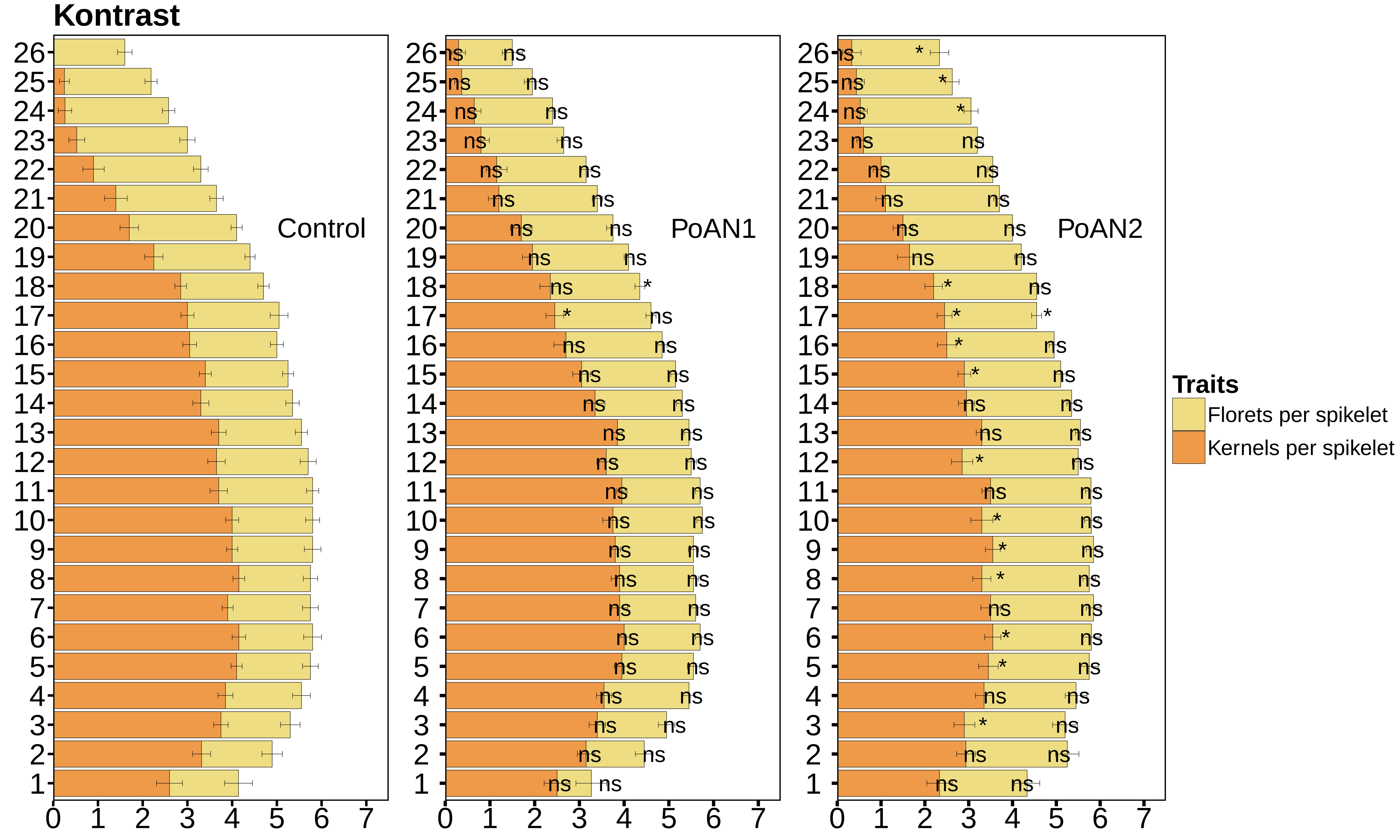
Kontrast
Control
PoAN1
PoAN2
Traits
Florets per spikelet
Kernels per spikelet

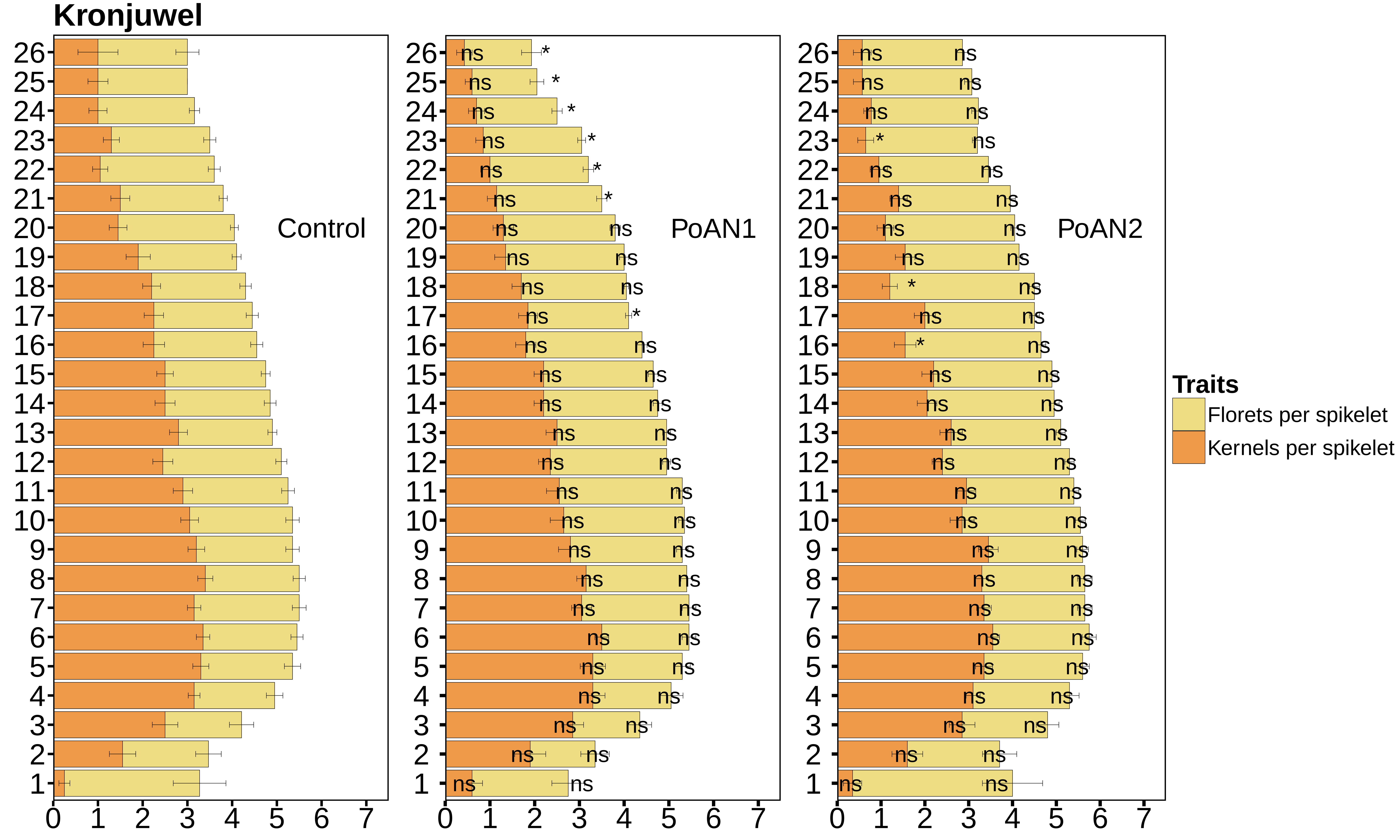

Kronjuwel
Control
PoAN1
PoAN2
Traits
Florets per spikelet
Kernels per spikelet

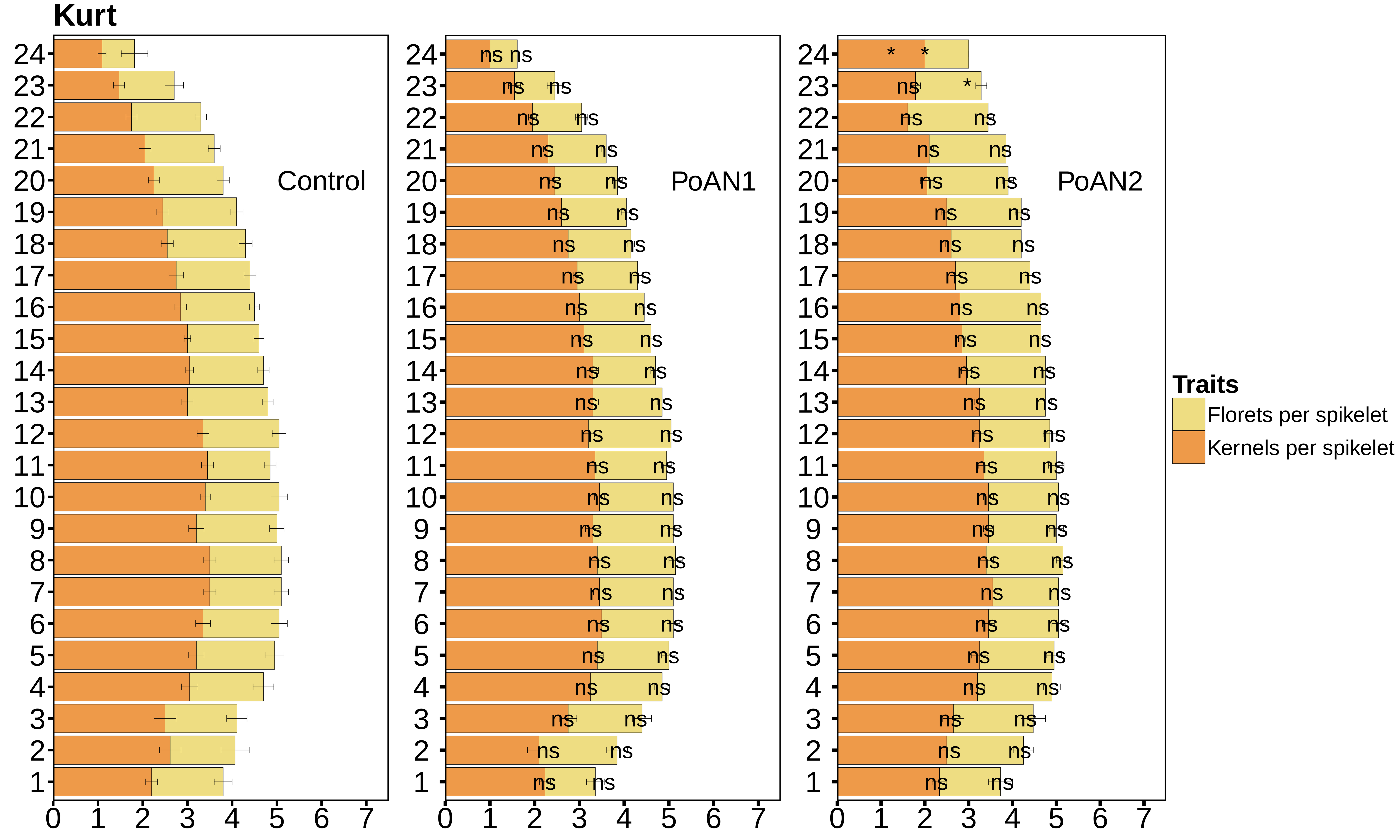

Kurt
Control
PoAN1
PoAN2
Traits
Florets per spikelet
Kernels per spikelet

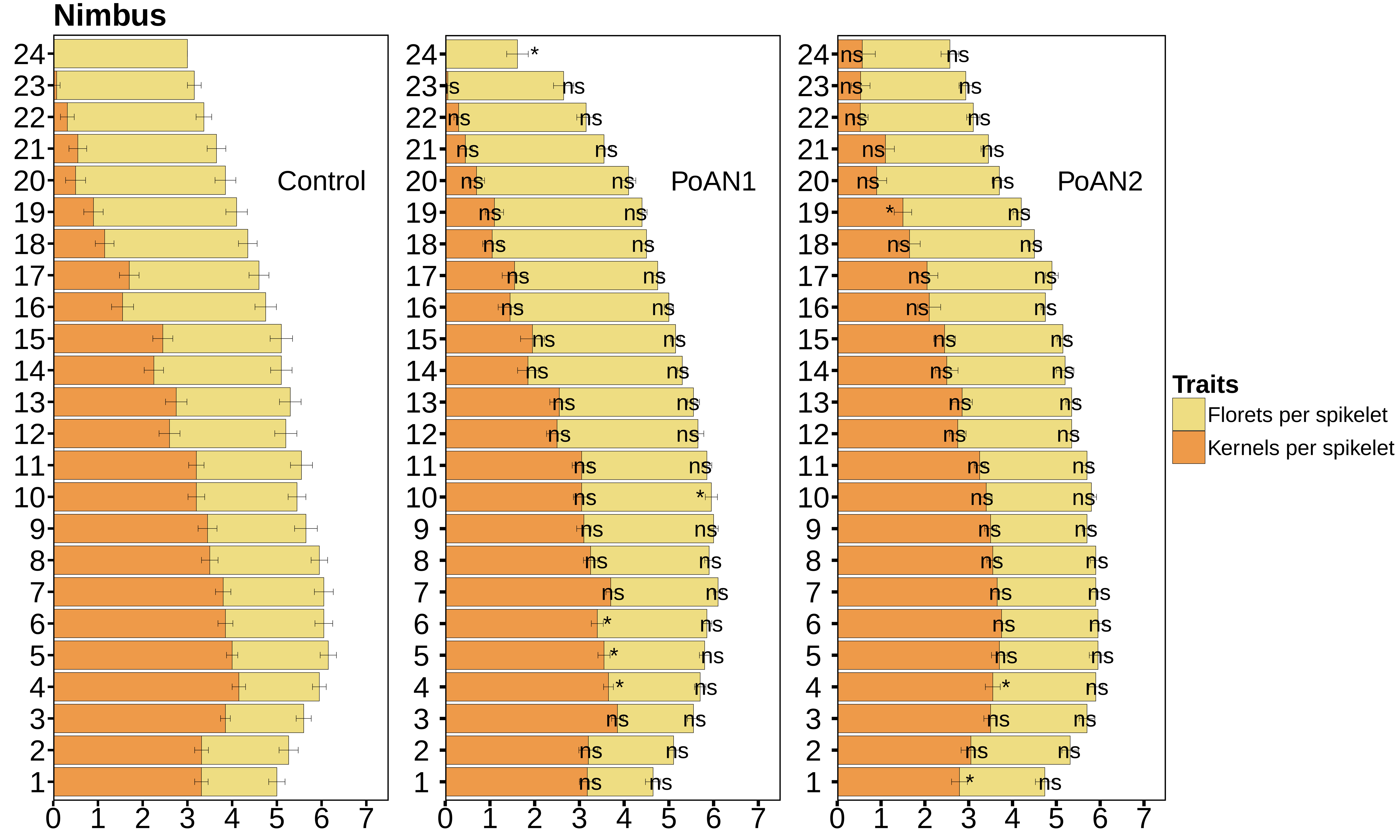
Nimbus
Control
PoAN1
PoAN2
Traits
Florets per spikelet
Kernels per spikelet

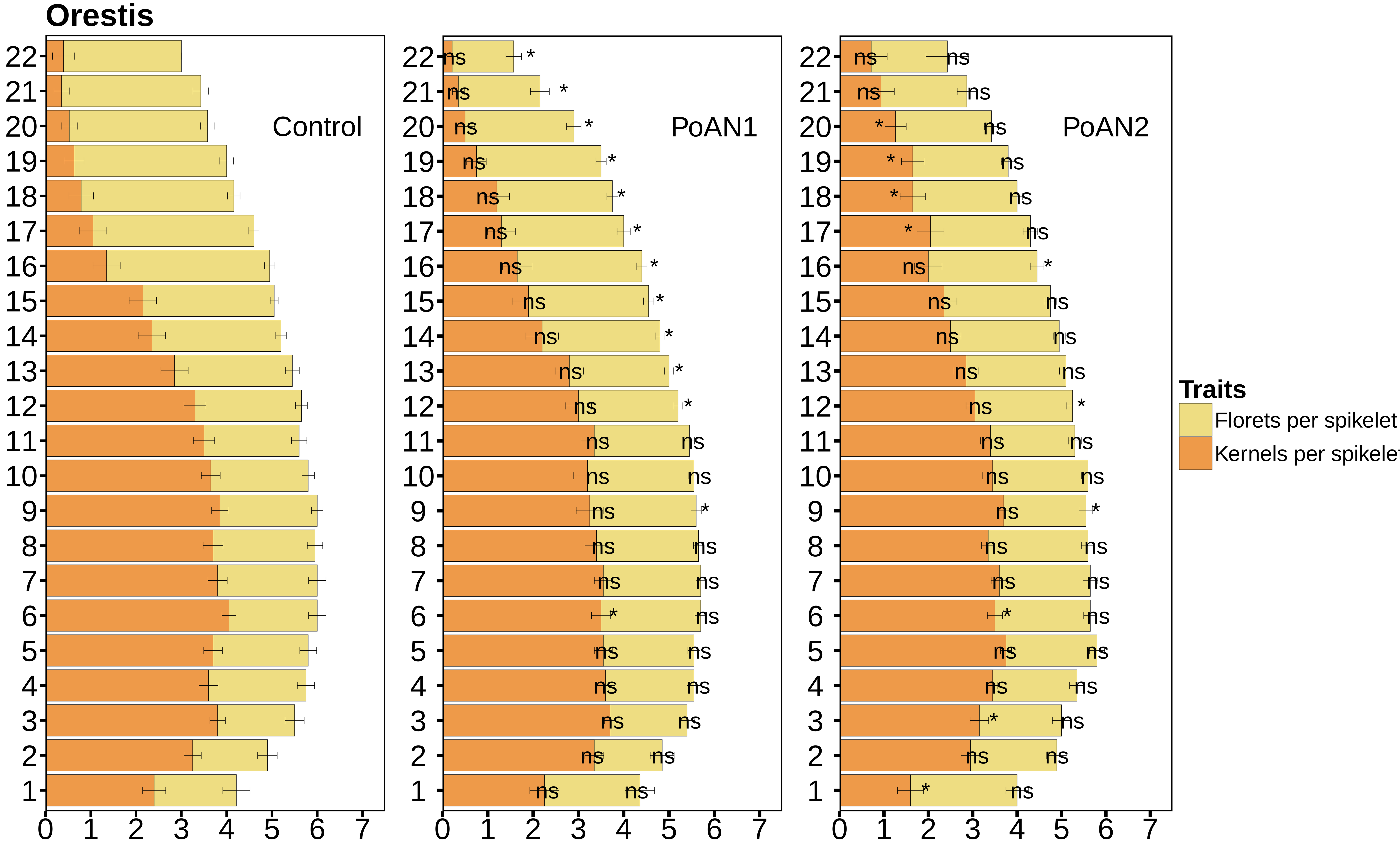

Orestis
Control
PoAN1
PoAN2
22
21
20
19
18
17
16
15
14
13
12
11
10
9
8
7
6
5
4
3
2
1
0 1 2 3 4 5 6 7
Traits
Florets per spikelet
Kernels per spikelet
ns
*

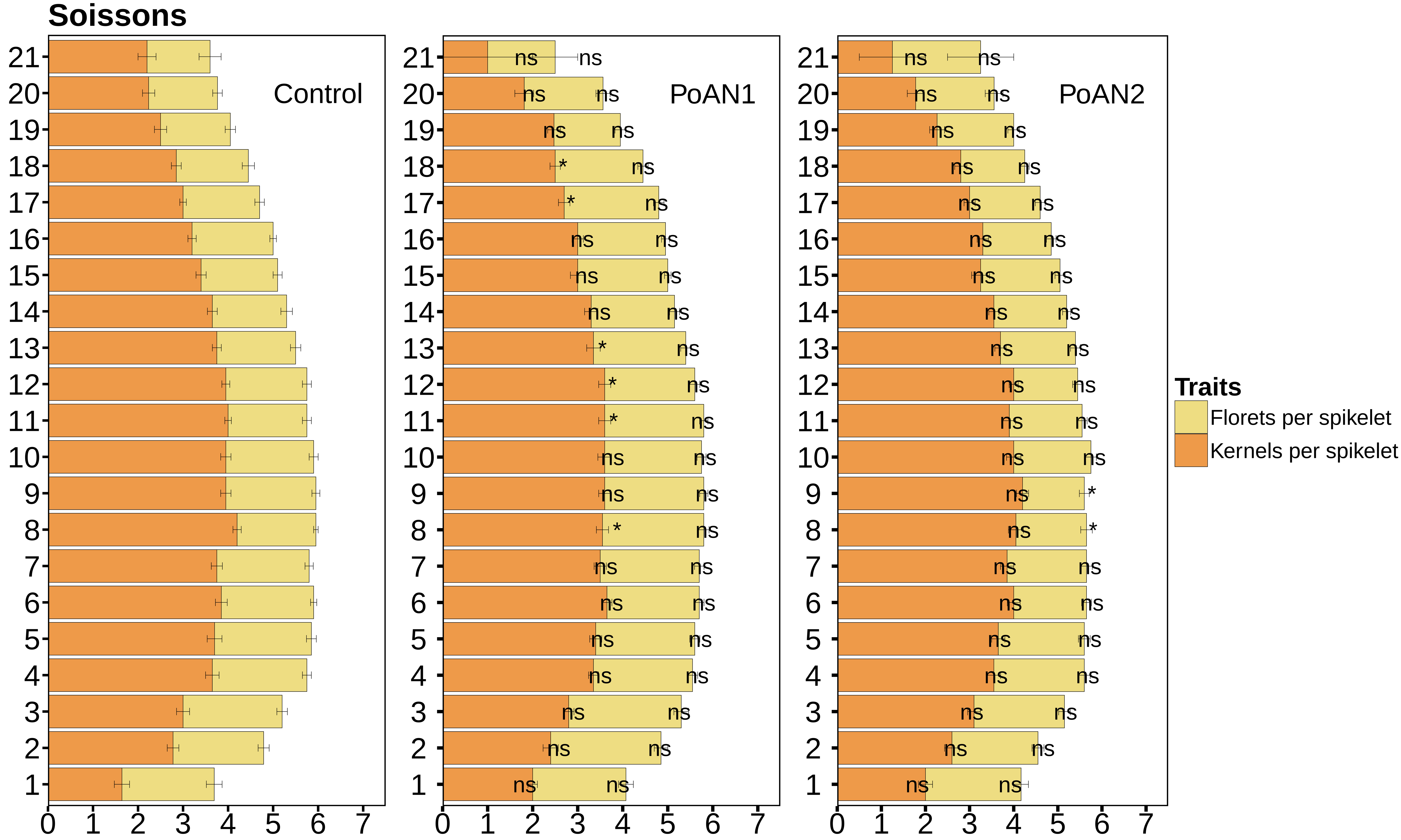
Soissons
Control
PoAN1
PoAN2
Traits
Florets per spikelet
Kernels per spikelet
21 20 19 18 17 16 15 14 13 12 11 10 9 8 7 6 5 4 3 2 1
0 1 2 3 4 5 6 7

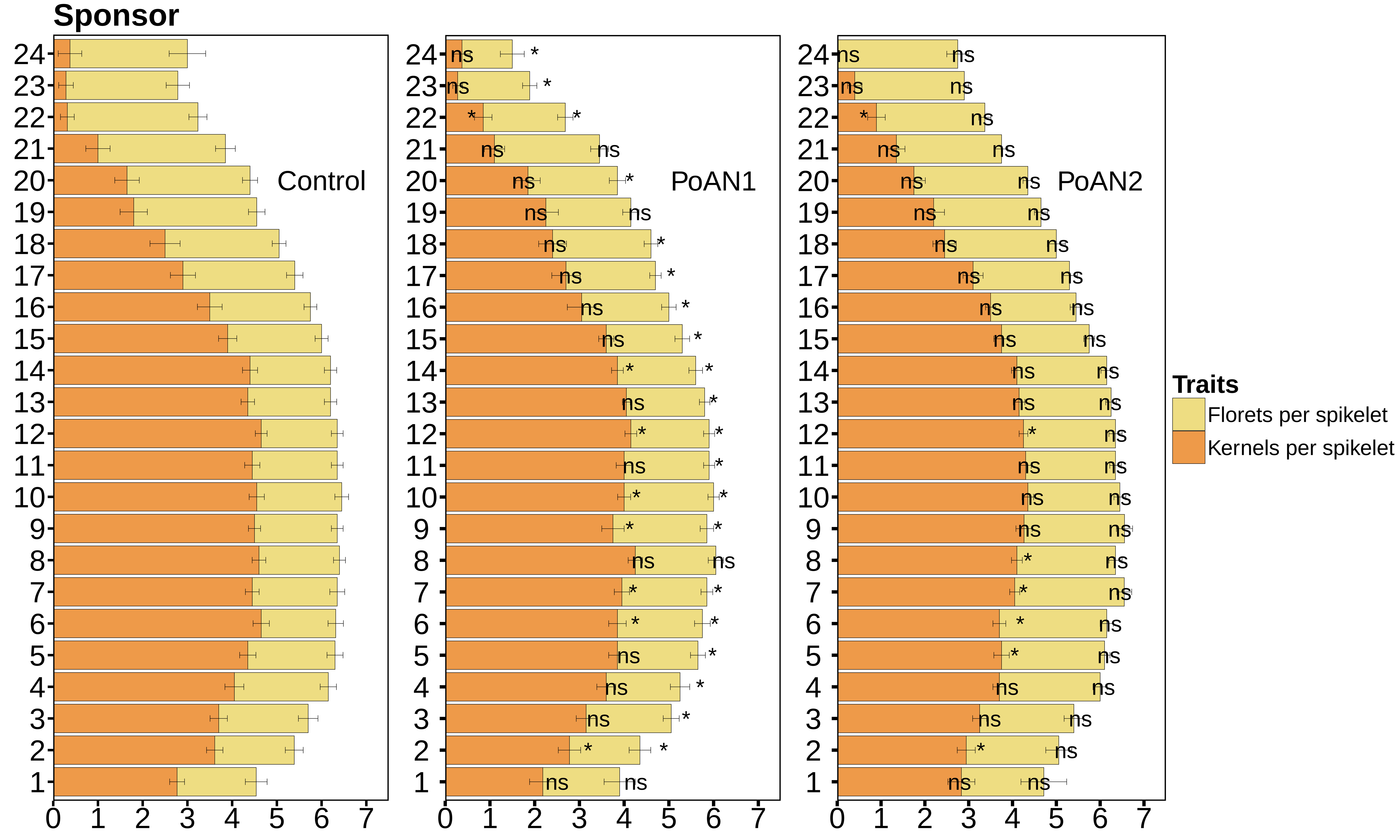
Sponsor
Control
PoAN1
PoAN2
Traits
Florets per spikelet
Kernels per spikelet
24
23
22
21
20
19
18
17
16
15
14
13
12
11
10
9
8
7
6
5
4
3
2
1
0 1 2 3 4 5 6 7

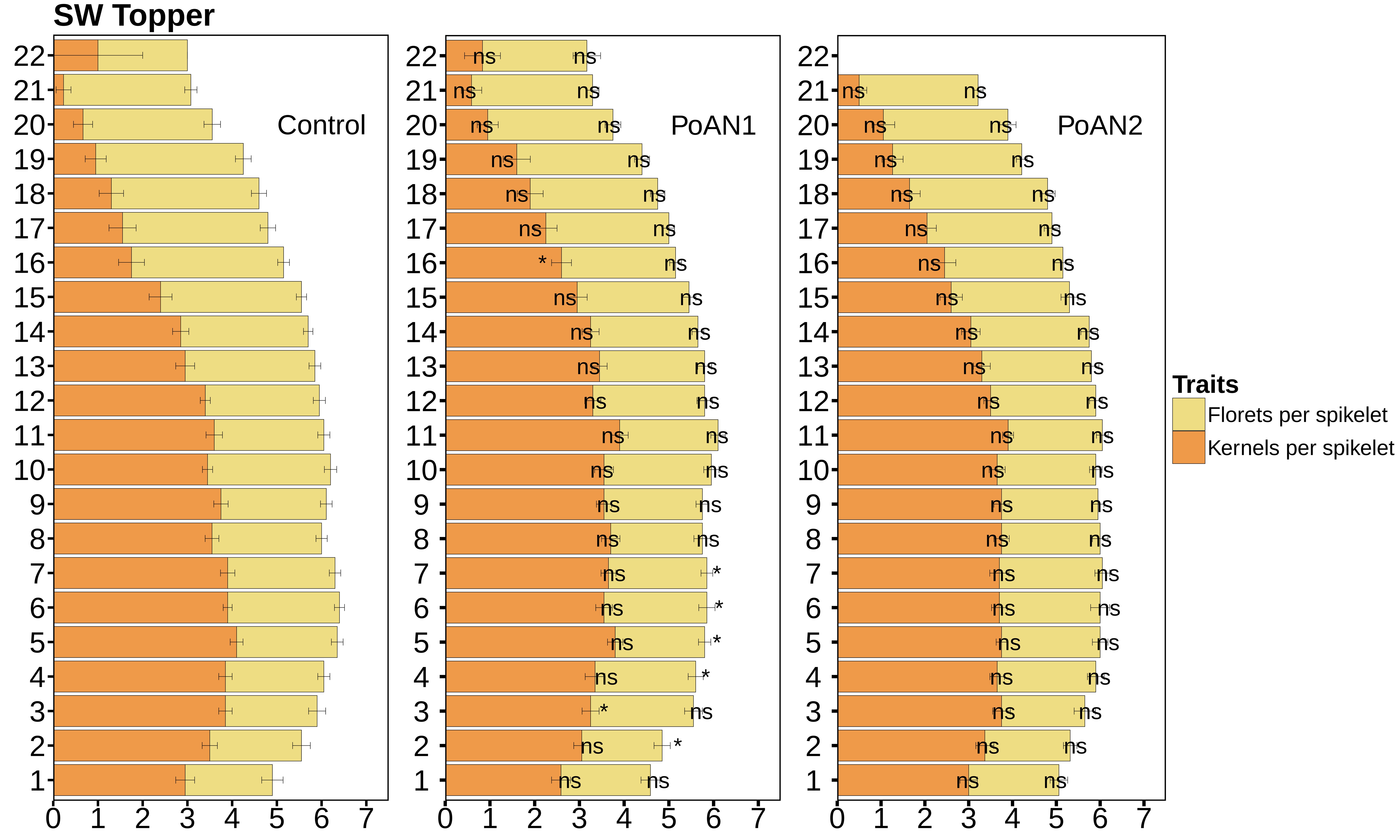
SW Topper
Control
PoAN1
PoAN2
Traits
Florets per spikelet
Kernels per spikelet

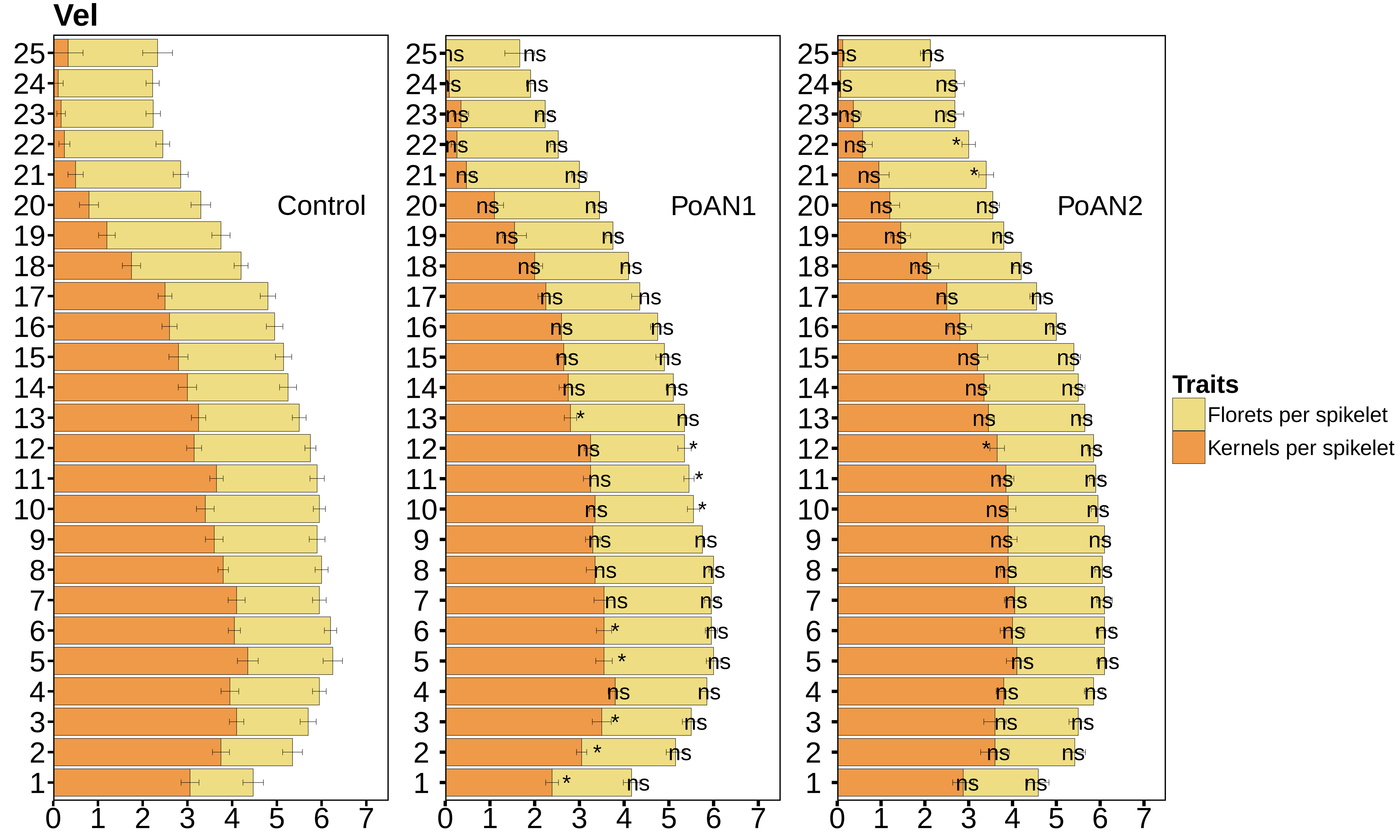
Vel
Control
PoAN1
PoAN2
25 24 23 22 21 20 19 18 17 16 15 14 13 12 11 10 9 8 7 6 5 4 3 2 1
0 1 2 3 4 5 6 7
Traits
Florets per spikelet
Kernels per spikelet

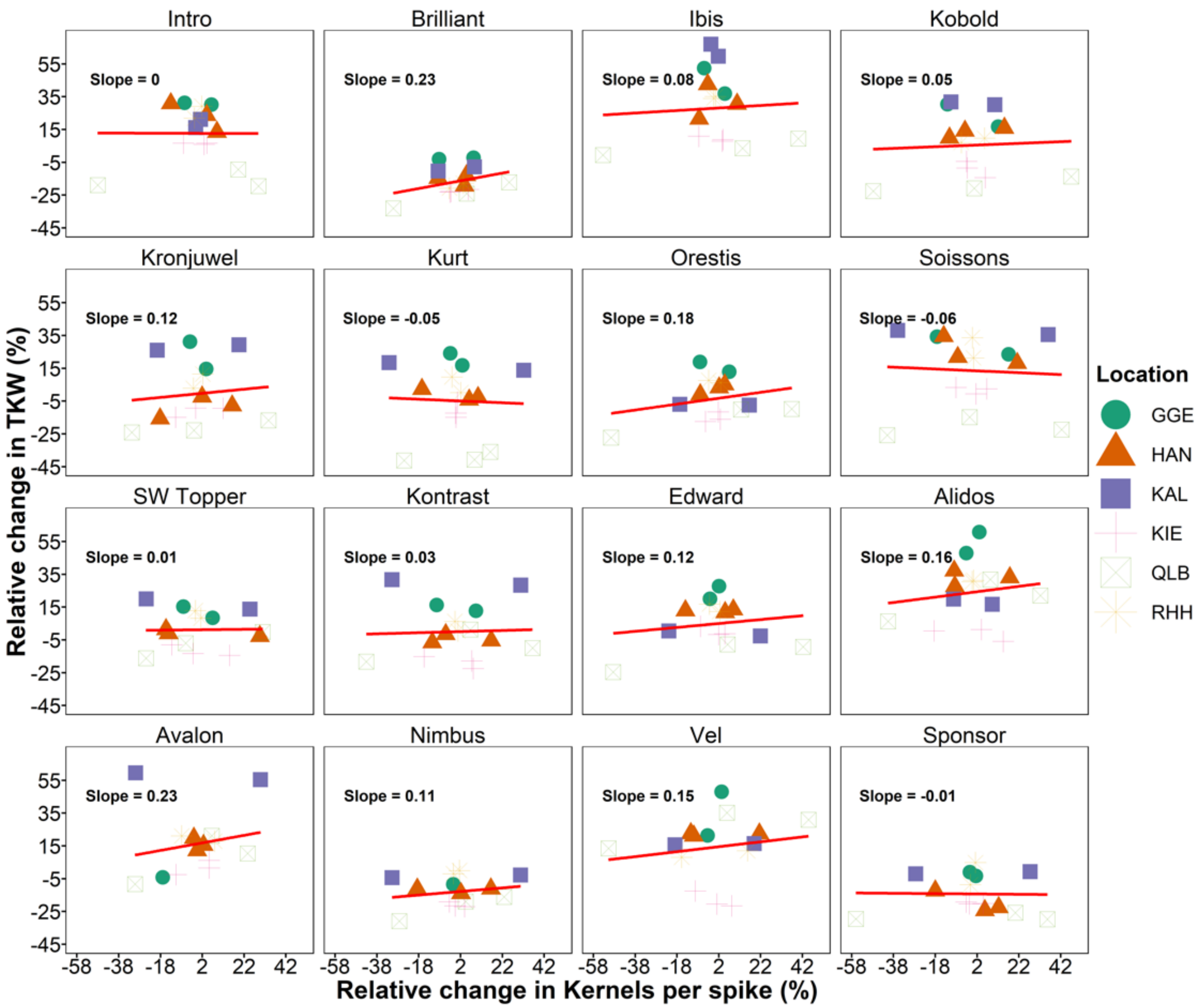


***Figure S6.*** Relationship between kernels per spike (relative change to mean per location, %, x-axis) and thousand kernel weight per plant (relative change to mean per location, %, y-axis) in a genotype (16 genotypes in total) to determine the compensatory effects. The colored points represent six locations and three years. The dataset and all othe details can be found at Sabir *et el*., 2023.

## Refrence


**Sabir K, Rose T, Wittkop B *et al.*** 2023. *Stage-specific genotype-by-environment interactions determine yield components in wheat [Data set]*. Zenodo https://doi.org/10.5281/zenodo.8248543: Zenodo.
